# Effect of host factors and COVID-19 infection on the humoral immune repertoire in treated HIV

**DOI:** 10.1172/jci.insight.166848

**Published:** 2023-03-08

**Authors:** Samuel R. Schnittman, Wonyeong Jung, Kathleen V. Fitch, Markella V. Zanni, Sara McCallum, Jessica Shih-Lu Lee, Sally Shin, Brandon J. Davis, Evelynne S. Fulda, Marissa R. Diggs, Francoise Giguel, Romina Chinchay, Anandi N. Sheth, Carl J. Fichtenbaum, Carlos Malvestutto, Judith A. Aberg, Judith Currier, Douglas A. Lauffenburger, Pamela S. Douglas, Heather J. Ribaudo, Galit Alter, Steven K. Grinspoon

**Affiliations:** 1 Division of Infectious Diseases, Department of Medicine, and; 2 Metabolism Unit, Massachusetts General Hospital, Boston, Massachusetts, USA.; 3 Ragon Institute of MGH, MIT, and Harvard, Cambridge, Massachusetts, USA.; 4 AIDS Clinical Trials Group Lab 01, Massachusetts General Hospital, Boston, Massachusetts, USA.; 5 Houston AIDS Research Team, University of Texas Health Science Center Houston, Houston, Texas, USA.; 6 Division of Infectious Diseases, Department of Medicine, Emory University School of Medicine, Atlanta, Georgia, USA.; 7 Division of Infectious Diseases, Department of Medicine, University of Cincinnati, Cincinnati, Ohio, USA.; 8 Division of Infectious Diseases, Department of Medicine, The Ohio State University Wexner Medical Center, Columbus, Ohio, USA.; 9 Division of Infectious Diseases, Department of Medicine, Icahn School of Medicine at Mount Sinai, New York, New York, USA.; 10 Division of Infectious Diseases, Department of Medicine, UCLA, Los Angeles, California, USA.; 11 Department of Biological Engineering, Massachusetts Institute of Technology, Cambridge, Massachusetts, USA.; 12 Duke Clinical Research Institute, Duke University, Durham, North Carolina, USA.; 13 Harvard T.H. Chan School of Public Health, Boston, Massachusetts, USA

**Keywords:** AIDS/HIV, COVID-19, Adaptive immunity, Immunoglobulins, Obesity

## Abstract

People with HIV (PWH) appear to be at higher risk for suboptimal pathogen responses and for worse COVID-19 outcomes, but the effects of host factors and COVID-19 on the humoral repertoire remain unclear. We assessed the antibody isotype/subclass and Fc-receptor binding Luminex arrays of non–SARS-CoV-2 and SARS-CoV-2 humoral responses among antiretroviral therapy–treated (ART-treated) PWH. Among the entire cohort, COVID-19 infection was associated with higher cytomegalovirus (CMV) responses (vs. the COVID^–^ cohort ), potentially signifying increased susceptibility or a consequence of persistent inflammation. Among the COVID^+^ participants, (a) higher BMI was associated with a striking amplification of SARS-CoV-2 responses, suggesting exaggerated inflammatory responses, and (b) lower nadir CD4 was associated with higher SARS-CoV-2 IgM and FcγRIIB binding capacity, indicating poorly functioning extrafollicular and inhibitory responses. Among the COVID-19^–^ participants, female sex, older age, and lower nadir CD4 were associated with unique repertoire shifts. In this first comprehensive assessment of the humoral repertoire in a global cohort of PWH, we identify distinct SARS-CoV-2–specific humoral immune profiles among PWH with obesity or lower nadir CD4^+^ T cell count, underlining plausible mechanisms associated with worse COVID-19–related outcomes in this setting. Host factors associated with the humoral repertoire in the COVID-19^–^ cohort enhance our understanding of these important shifts among PWH.

## Graphical abstract

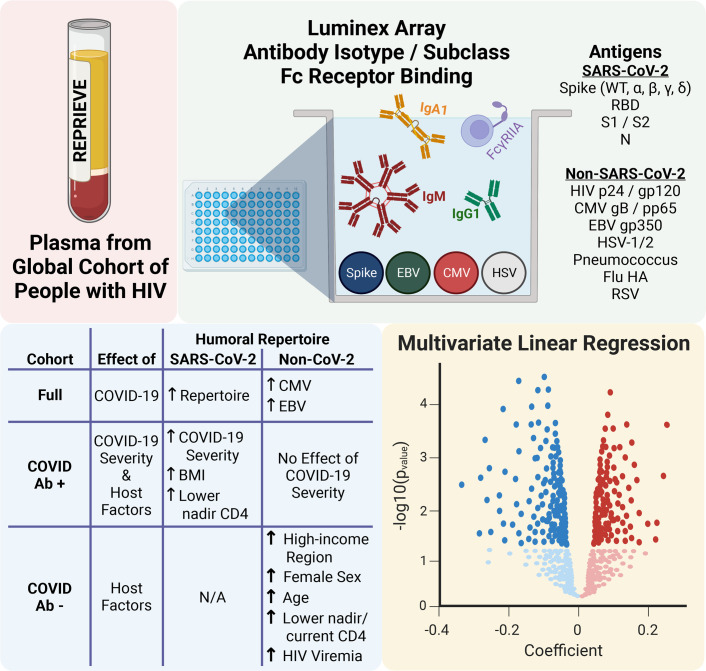

## Introduction

As HIV preferentially infects and depletes CD4^+^ T cells — leading to innate and adaptive immune dysfunction — it also impacts the humoral immune repertoire. HIV is associated with increased immune activation and lymphoid hyperplasia, leading to polyclonal hypergammaglobulinemia, B cell exhaustion, and impaired T follicular helper cell function (1, 2). HIV is also associated with lymphoid fibrosis and germinal center architectural distortion, which may cause long-lasting damage despite antiretroviral therapy (ART) (3–6). While ART significantly restores immune function, improves survival, and protects against opportunistic infections and other AIDS- and non–AIDS-related conditions in people with HIV (PWH), immunologic defects persist and predict morbidity and mortality (7). Functionally, PWH tend to have suboptimal vaccine responses and less robust or durable responses to some pathogens, including SARS-CoV-2 (3, 4, 8–13). This is particularly true in those with lower CD4^+^ T cell counts with incomplete immune recovery (11–15). Age, sex, and regional differences have also been associated with these humoral responses, as they intersect with immunosenescence, sex hormones, host genetics, and lymphoid fibrosis (3, 8, 16–18). Nonetheless, a more comprehensive understanding of the broader humoral repertoire in HIV is needed (19, 20).

With persistent immune defects despite ART, PWH appear to be at higher risk for more severe outcomes associated with COVID-19 (21–25). Preliminary findings also suggest that lower CD4 and HIV viremia are associated with worse outcomes after COVID-19 in PWH (22, 23, 25, 26). However, the underlying mechanisms and effects of HIV-related factors on the SARS-CoV-2 humoral immune repertoire is unknown (12, 27, 28). Moreover, while age, male sex, and obesity have been associated with severe illness in the general population and are posited to have similar effects in PWH, the impact of these host factors on the SARS-CoV-2 humoral repertoire is similarly unknown in HIV (23, 26, 29–31). Finally, several studies have linked cytomegalovirus-specific (CMV-specific) and EBV-specific responses or serostatus to an increased risk of COVID-19 infection, severity, and long COVID symptoms or postacute sequelae of COVID-19 (PASC) in the general population (32–34). Given not only the higher rates of CMV seropositivity, but also the possibility of increased rates of long COVID or PASC in PWH compared with the general population, the association between COVID-19 and the non–SARS-CoV-2 humoral immune repertoire in treated HIV merits further investigation (35–37).

To address these gaps, we leveraged the ongoing Randomized Trial to Prevent Vascular Events in HIV (REPRIEVE, NCT02344290) to assess the associations among host factors, COVID-19, and the SARS-CoV-2– and non–SARS-CoV-2–related humoral repertoires (38). From this large global cohort of ART-treated PWH, we sampled available blood to provide a comprehensive humoral immune profile and to better evaluate the effector capacity of each antigen specificity, assessing antibody isotypes, subclasses, and antibody-specific Fc γ receptor (FcγR) binding ability to SARS-CoV-2, HIV, common respiratory pathogens, and herpesviruses whose effects may be amplified in PWH. We determined COVID-19 status by antibody testing and analyzed critical host factors and HIV-related indices in relationship to shifts in the SARS-CoV-2 and non–SARS-CoV-2 repertoires. This work advances our knowledge of the potential mechanisms underlying clinical outcomes related to COVID-19 among PWH. Moreover, this analysis adds to our understanding of how such clinical factors relate to the broader humoral repertoire in treated HIV.

## Results

### Recruitment from REPRIEVE.

REPRIEVE (NCT02344290) is a global cardiovascular prevention trial that recruited more than 7,500 participants from 12 countries. Full inclusion and exclusion criteria and baseline participant characteristics have been previously reported (38, 39). Briefly, PWH 40–75 years of age on stable ART with a current CD4^+^ T cell count > 100 cells/mm^3^ and low-to-moderate traditional cardiovascular disease risk were randomized to pitavastatin versus placebo with longitudinal follow-up for cardiovascular events. Starting April 2020, targeted data related to COVID-19 diagnoses, symptoms, and adverse events were collected in participants every 4 months. Available samples collected May 5, 2020, through February 22, 2021, were included. Participants who received vaccination against SARS-CoV-2 were excluded.

### Participant characteristics.

**Figure 1:**
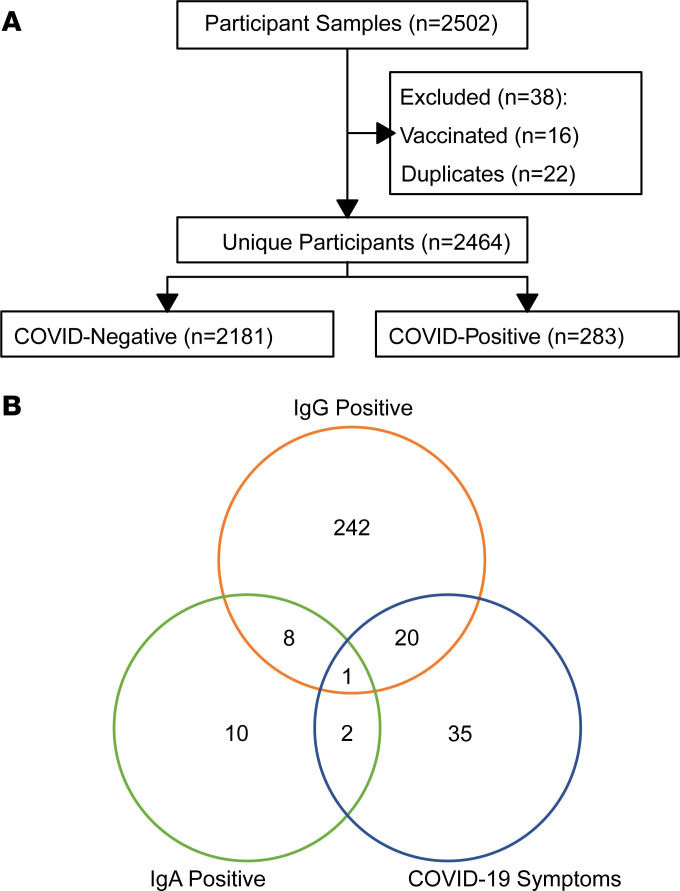
Study design. (**A**) Consort diagram. (**B**) Venn diagram describing breakdown of RBD IgG/IgA-positive participants based on RBD IgG/IgA status and COVID-19–like symptoms.

In total, 2,502 plasma samples were available from annual study visits during the sampling period. Multiple samples were available for 22 participants, of which the latest sample was used. Sixteen samples were excluded from participants who had received any SARS-CoV-2 vaccination prior to sampling. The final cohort included 2,464 REPRIEVE participants, representing 33% of the underlying REPRIEVE enrollment and 83% of participants who had a study visit during the sampling period (Table 1 and Figure 1A). Median age was 53 years, and 35% were women. Half were from a high-income global burden of disease (GBD) region (United States or Canada), and half were from Latin America/Caribbean (20%), Asia (15%), or sub-Saharan Africa (14%), with a mix of racial groups, including 68% non-White participants. While the median current CD4^+^ T cell count was 649 (quartile 1 [Q1] to Q3, 483–849) , 50% had a nadir CD4 < 200 cells/mm^3^. All were receiving ART, 53% had been on ART for more than 10 years, and 46% were on an integrase strand transfer inhibitor–based (INSTI-based) regimen. Almost all (97%) participants were virally suppressed, defined as below the assay’s limit of quantification or < 400 copies/mL (given global assay variability). Participant characteristics were similar to those in the overall REPRIEVE cohort (39).

### COVID-19 classification.

Given the initial lack of clarity and the evolving and variable nature of COVID-19 case ascertainment globally, we defined COVID-19 cases based on antibody positivity. Antibody positivity was prespecified based on a SARS-CoV-2 receptor-binding domain (RBD) IgG and/or IgA more than 5 SD above plate-specific negative controls on ELISA (see Methods) (40). The full cohort (*n* = 2,464) was then subdivided into COVID-19^+^ (283, 11.5%) and COVID-19^–^ (*n* = 2,181, 88.5%) cohorts. Of the COVID-19^+^ participants, 271 had a positive RBD IgG, 21 had a positive RBD IgA, and 9 were positive for both (Figure 1B).

Targeted COVID-19 symptom and severity assessments from a standardized COVID-19 questionnaire were performed every 4 months with each study visit. Participants were asked to report adverse events, including clinical diagnosis of COVID-19 and/or a positive SARS-CoV-2 rapid antigen detection test or PCR test. Adverse events were graded for severity on the ordinal scale of mild, moderate, severe, potentially life-threatening, or resulting in death, per the Division of AIDS (DAIDS) Adverse Events Grading Tables (https://rsc.niaid.nih.gov/sites/default/files/daidsgradingcorrectedv21.pdf). The overwhelming majority (92%) of the COVID-19^+^ cohort was asymptomatic or did not report symptoms. Mild, moderate, and severe disease were reported in 3.2%, 4.6%, and 0.4% of the COVID-19^+^ cohort, respectively. Those who were RBD IgG^+^ and/or IgA^+^ but did not report a COVID-19 diagnosis were included in the category “asymptomatic or not reported.” A small portion (*n* = 35/2,464; 1.4%) of SARS-CoV-2 RBD IgG^–^/IgA^–^ participants reported mild, moderate, or severe adverse events associated with prior COVID-19, though we could not confirm these diagnoses. In those who reported a clinical diagnosis of COVID-19, median time from diagnosis to sampling was 13 weeks (Q1–Q3, 10–23 weeks).

### Statin use.

Given the ongoing nature of the REPRIEVE trial, our analysis remains blinded to participant randomization to pitavastatin or placebo. Nonetheless, our independent unblinded statistician confirmed that the proportion of COVID-19^+^ or COVID-19^–^ participants by randomized group was within a prespecified threshold of ± 5%, reducing concern for significant confounding.

### Univariate associations with the humoral immune repertoire.

We first analyzed host and HIV-specific factors in univariate analysis across antibody isotypes (IgA, IgM, and IgG), subclasses (IgG1, IgG3, IgG4, IgA1), and antibody-specific FcγR (FcγRIIA, FcγRIIB, FcγRIIIA) binding capacity for the following non–SARS-CoV-2–related antigens: influenza hemagglutinin (HA), respiratory syncytial virus (RSV), pneumococcus, HIV gp120 clade B/C consensus, HIV p24 clade B HXBc2, herpes simplex virus 1 (HSV-1), HSV-2, EBV glycoprotein 350 (EBV gp350), CMV glycoprotein B (CMV gB), and CMV phosphoprotein 65 (CMV pp65). The same antibody isotypes, subclasses, and FcγR binding abilities were assessed for the following SARS-CoV-2–related antigens: spike, spike protein subunits S1 and S2, RBD, nucleocapsid (N), and spike α, -β, -δ, and -γ variants (see Methods).

**Figure 2:**
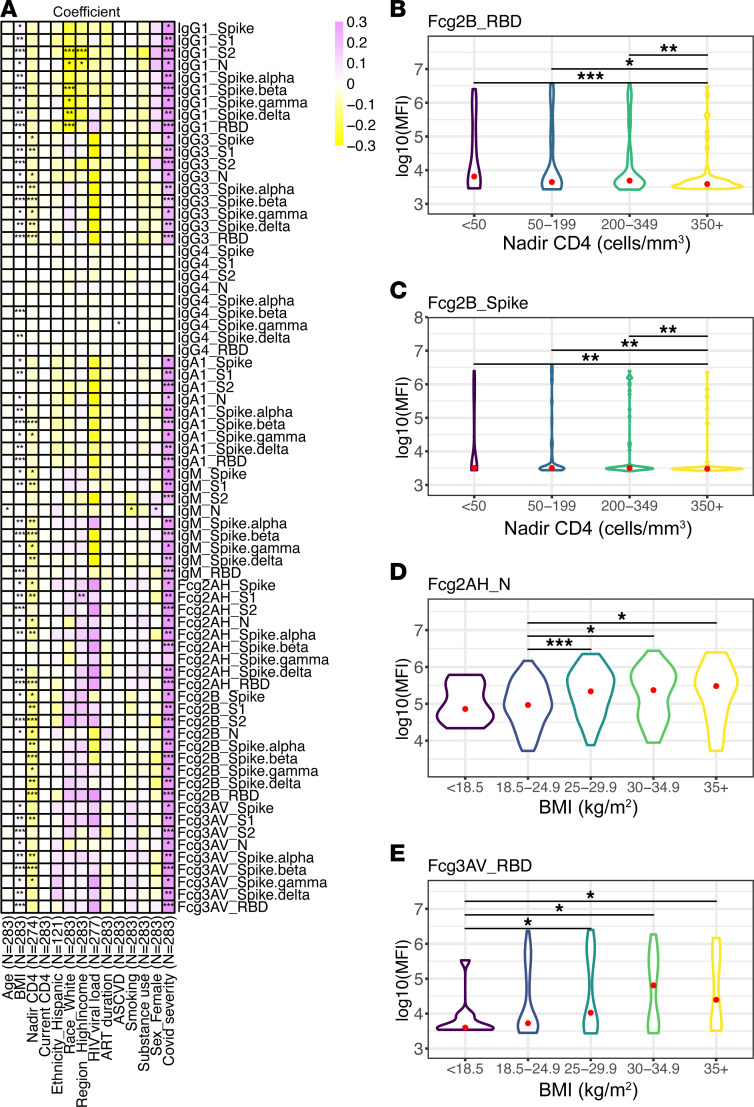
Univariate associations among COVID-19^+^ participants. (**A**) Univariate heatmap relating COVID-19 severity and host factors to SARS-CoV-2–specific antibody isotype and subclass and Fc-receptor binding. Coefficients derived from unadjusted linear regression modeling. (**B** and **C**) Violin plots of RBD-specific FcγRIIB (**B**) and Spike-specific FcγRIIB (**C**) across nadir CD4 groups are shown. (**D** and **E**) Violin plots of N-specific FcγRIIA (**D**) and RBD-specific FcγRIIIA (**E**) across BMI groups. Significance testing was performed via Wilcoxon rank-sum test and is shown as *P < 0.05, **P < 0.01, or ***P < 0.001.

Univariate analysis, performed with unadjusted linear regression and Wilcoxon rank-sum test, revealed multiple significant predictors across all antigens (Figure 2A and Supplemental Figures 1–3; supplemental material available online with this article; https://doi.org/10.1172/jci.insight.166848DS1). While we saw multiple univariate associations with sex and nadir CD4 in the COVID-19^–^ cohort, we observed an interesting pattern of higher SARS-CoV-2–specific antibodies that engage FcγRIIB among those with lower nadir CD4 in the COVID-19^+^ group (Figure 2, B and C). We also noted an association between higher BMI and higher SARS-CoV-2 antibody and FcγR binding capacity in the COVID-19^+^ cohort (Figure 2, D and E). Highlighted statistically significant responses associated with host factors in the COVID-19^+^ and COVID-19^–^ cohorts in univariate analysis are shown in Supplemental Figures 4–9. Based on univariate analysis and a priori assumptions, multivariate modeling was implemented to assess the relationship between (a) COVID-19 and (b) key host factors and the humoral immune repertoire, adjusting for age, natal sex, GBD region, nadir CD4, HIV viral load (VL).

### Effect of COVID-19 on the humoral immune repertoire.

To assess the impact of COVID-19 and other host factors on the humoral repertoire, we performed multivariate linear regression modeling, with the dependent variable as the antibody isotype, subclass, or FcγR binding ability adjusted for potential confounders as above. Adjusted coefficients of the predictors were depicted graphically in a volcano plot, with FDR-corrected *P* values by the Benjamini-Hochberg method (41).

**Figure 3:**
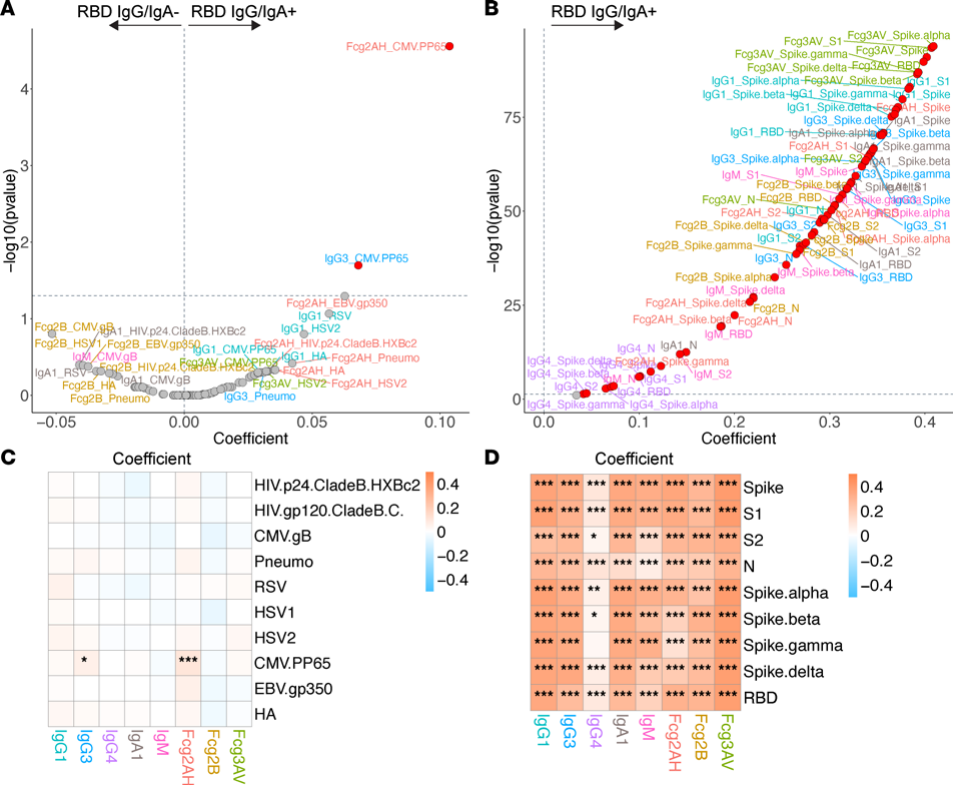
Volcano plots and heatmaps of effect of SARS-CoV-2 RBD IgG/IgA positivity on the humoral immune repertoire among all participants. (**A** and **B**) Volcano plots of effect of SARS-CoV-2 RBD IgG/IgA positivity on the non–SARS-CoV-2 humoral repertoire (**A**) and SARS-CoV-2 humoral repertoire (**B**) among all participants. Volcano plots constructed from linear regression models, adjusted for age, sex, GBD region, nadir CD4, and HIV viral load, with horizontal dashed line of significance displayed for FDR-corrected *P* = 0.05. Responses higher in the antibody-positive fall toward the right of the vertical dashed line, while responses higher in the antibody-negative fall toward the left of the vertical dashed line. (**C** and **D**) Respective heatmaps of the volcano plot coefficients for the non–SARS-CoV-2 (**C**) and SARS-CoV-2 (**D**) humoral responses. Coefficients > 0 reflect higher antibody responses in the antibody-positive participants, while coefficients < 0 reflect higher antibody responses in the antibody-negative participants. Significance in the heatmaps is shown as FDR-corrected *P < 0.05, **P < 0.01, or ***P < 0.001. Specific antibody isotype, subclass, and Fc-receptor responses are color-coded between the volcano plots and heatmaps.

We first asked to what extent COVID-19 affects SARS-CoV-2– and non–SARS-CoV-2–related humoral responses. As expected, among the full cohort, COVID-19 infection was associated with higher levels of virtually all SARS-CoV-2–related antibodies and FcγR binding capacity (Figure 3, B and D).

Among the entire cohort, we observed that COVID-19 did not affect most of the tested non–SARS-CoV-2–related humoral immune repertoire (Figure 3, A and C). COVID-19 was associated with significantly higher CMV pp65–specific IgG3 and FcγRIIA binding ability. The ability of EBV gp350–specific antibodies to bind FcγRIIA appeared higher in those with COVID-19 infection, though this bordered on FDR-corrected statistical significance (*P* = 0.051).

### COVID-19 severity.

**Figure 4:**
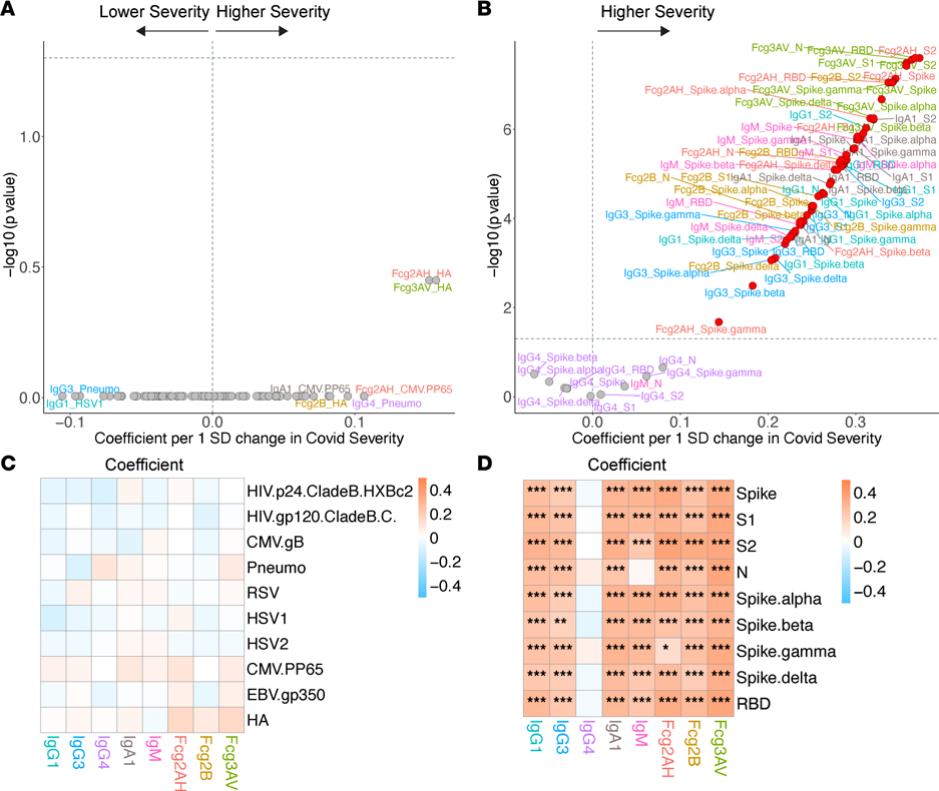
Volcano plots and heatmaps of effect of COVID-19 severity on the humoral immune repertoire among COVID-19^+^ participants. (**A** and **B**) Adjusted volcano plots of effect of COVID-19 severity on the non–SARS-CoV-2 humoral repertoire (**A**) and SARS-CoV-2 humoral repertoire (**B**) among COVID-19^+^ participants. Coefficients reflect the effect of a 1 SD increase in severity, which was *Z* scored for each participant from the ordinal scale of none reported/asymptomatic, mild, moderate, or severe. (**C** and **D**) Respective heatmaps of the volcano plot coefficients for the non–SARS-CoV-2 (**C**) and SARS-CoV-2 (**D**) humoral responses. Significance in the heatmaps is shown as FDR-corrected *P < 0.05, **P < 0.01, or ***P < 0.001.

We next assessed how COVID-19 severity was associated with the SARS-CoV-2– and non–SARS-CoV-2–related humoral profile. In multivariate modeling, a *Z* score was created for the independent predictor of COVID-19 severity (see Methods). Despite the overabundance of asymptomatic (or not reported) COVID-19, worse disease severity was associated with higher SARS-CoV-2–specific antibodies and antibody-specific FcγR binding capacity in the COVID-19^+^ cohort (Figure 4, B and D). In contrast, among the COVID-19^+^ cohort, we did not identify any association between COVID-19 severity and the non–SARS-CoV-2–related humoral immune repertoire (Figure 4, A and C).

### Assessment of host- and HIV-specific factors on the SARS-CoV-2 and non–SARS-CoV-2 humoral repertoire.

After assessing the effect of COVID-19 in the overall cohort, we individually assessed host-specific and HIV-specific factors and their impact on SARS-CoV-2 and non–SARS-CoV-2 responses in the COVID-19^+^ and COVID-19^–^ cohorts, respectively.

### Natal sex.

**Figure 5:**
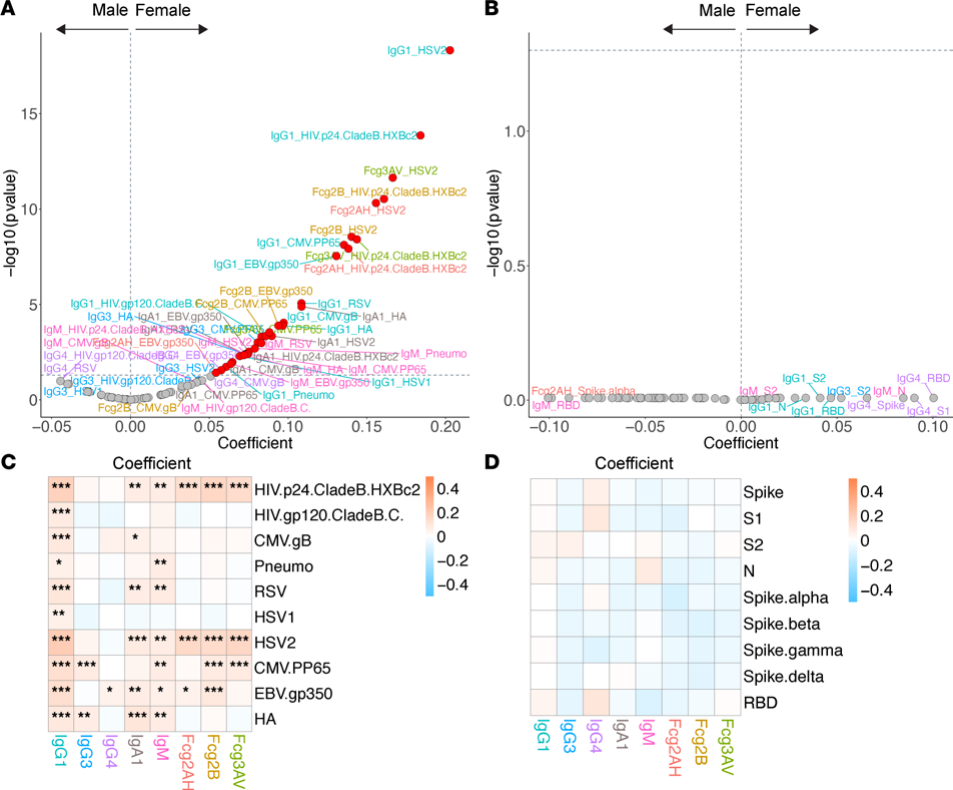
Volcano plots and heatmaps of effect of natal sex on the humoral immune repertoire. (**A** and **B**) Adjusted volcano plots of effect of natal sex on the non–SARS-CoV-2 humoral repertoire (**A**) among the COVID-19^–^ cohort and SARS-CoV-2 humoral repertoire (**B**) among the COVID-19^+^ cohort. (**C** and **D**) Respective heatmaps of the volcano plot coefficients for the non–SARS-CoV-2 (**C**) and SARS-CoV-2 (**D**) humoral responses. Significance in the heatmaps is shown as FDR-corrected *P < 0.05, **P < 0.01, or ***P < 0.001.

Among the COVID-19^+^ cohort, no shifts in the SARS-CoV-2–related humoral repertoire were observed by natal sex (Figure 5, B and D). We did note prominent sex-related humoral differences among the COVID-19^–^ participants (Figure 5, A and C). After adjustment, IgG1 (and most IgA1) levels were higher in women for all non–SARS-CoV-2 antigens assessed. Women tended to have greater repertoire shifts toward the herpesviruses, including EBV, CMV, and HSV-2, as well as HA, RSV, and HIV-specific responses.

### Age.

**Figure 6:**
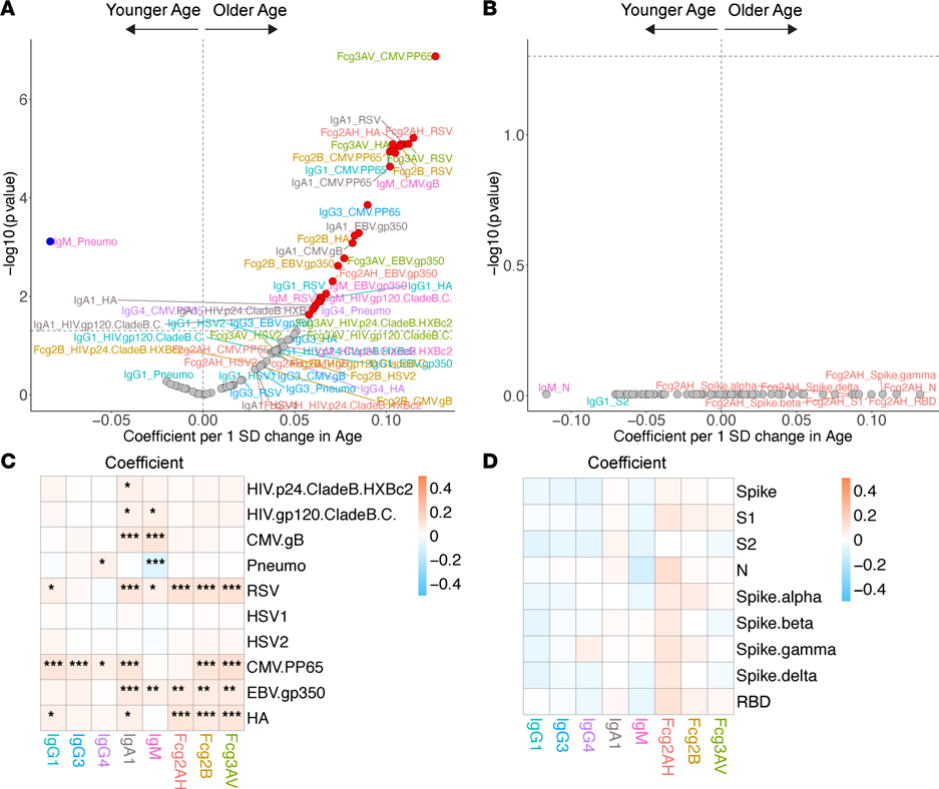
Volcano plots and heatmaps of effect of age on the humoral immune repertoire. (**A** and **B**) Adjusted volcano plots of effect of age on the non–SARS-CoV-2 humoral repertoire (**A**) among the COVID-19^–^ cohort and SARS-CoV-2 humoral repertoire (**B**) among COVID-19^+^ cohort. Coefficients reflect the effect of a 1 SD increase in age, which was *Z* scored for each participant. (**C** and **D**) Respective heatmaps of the volcano plot coefficients for the non–SARS-CoV-2 (**C**) and SARS-CoV-2 (**D**) humoral responses. Significance in the heatmaps is shown as FDR-corrected *P < 0.05, **P < 0.01, or ***P < 0.001.

Among the COVID-19^+^ cohort, we observed no association between age and SARS-CoV-2 humoral responses (Figure 6, B and D). Among the COVID-19^–^ cohort, IgA responses to most antigens assessed were higher with older age (Figure 6, A and C). Older age was associated with higher antibody responses and FcγR binding ability to EBV and CMV, but not HSV. We also observed an association between older age and heightened antibody binding capacity and antibody-specific FcγRs binding capacity to influenza HA and RSV, likely related to higher numbers of exposures throughout one’s lifetime. There were no significant differences in pneumococcus responses, perhaps as all participants are indicated for pneumococcal immunizations based on HIV infection, irrespective of age.

### BMI.

**Figure 7:**
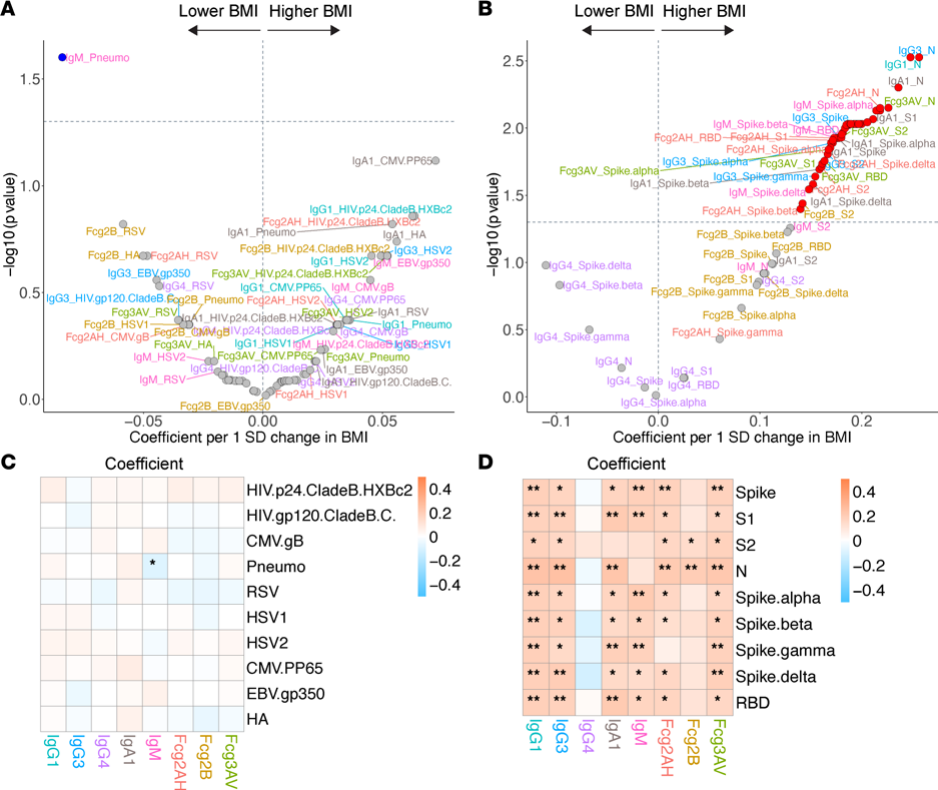
Volcano plots and heatmaps of effect of BMI on the humoral immune repertoire. (**A** and **B**) Adjusted volcano plots of effect of BMI on the non–SARS-CoV-2 humoral repertoire (**A**) among the COVID-19^–^ cohort and SARS-CoV-2 humoral repertoire (**B**) among COVID-19^+^ cohort. Coefficients reflect the effect of a 1 SD increase in BMI, which was *Z* scored for each participant. (**C** and **D**) Respective heatmaps of the volcano plot coefficients for the non–SARS-CoV-2 (**C**) and SARS-CoV-2 (**D**) humoral responses. Significance in the heatmaps is shown as FDR-corrected *P < 0.05 or **P < 0.01.

Among COVID-19^+^ participants, higher BMI was associated with a striking amplification of the SARS-CoV-2 humoral repertoire, with significantly higher IgG, IgA, and IgM levels and almost all antibody-specific FcγRIIA binding abilities (Figure 7, B and D). Interestingly, there were no differences in levels of IgG4 and most antibodies’ capacities to bind FcγRIIB based on BMI (a trend toward higher IgG4 levels with lower BMI). No significant BMI effects were observed among COVID-19^–^ participants for non–SARS-CoV-2–related antigens (Figure 7, A and C).

### GBD region.

**Figure 8:**
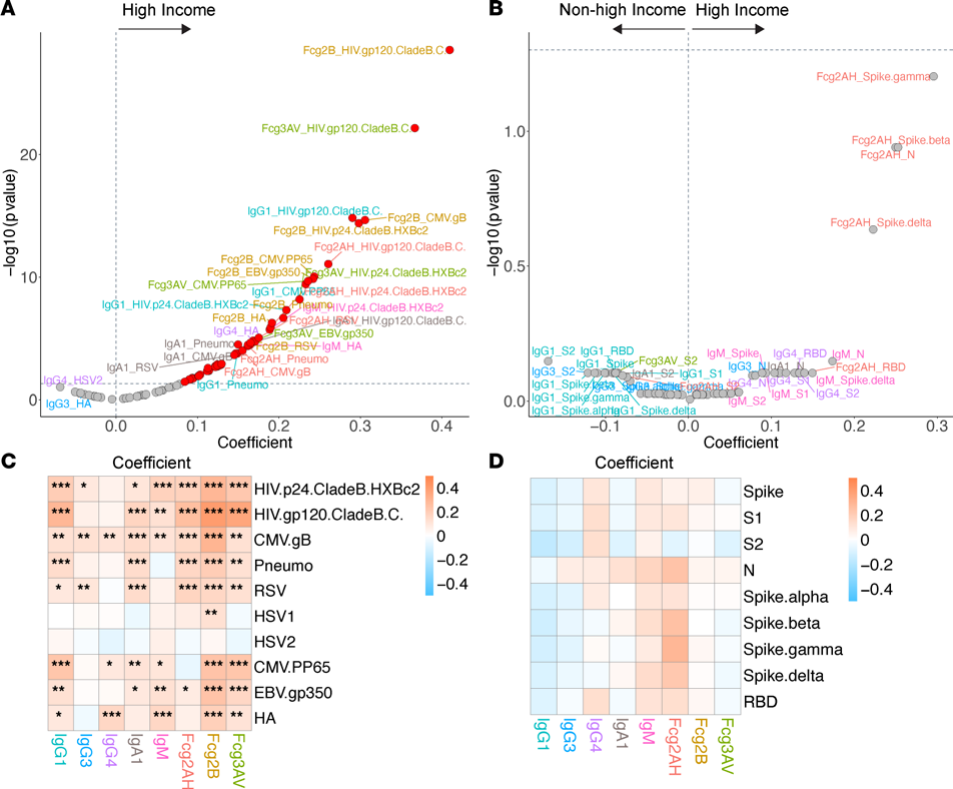
Volcano plots and heatmaps of effect of GBD region on the humoral immune repertoire. (**A** and **B**) Adjusted volcano plots of effect of high-income GBD region (vs. non–high-income) on the non–SARS-CoV-2 humoral repertoire (**A**) among the COVID-19^–^ cohort and SARS-CoV-2 humoral repertoire (**B**) among the COVID-19^+^ cohort. (**C** and **D**) Respective heatmaps of the volcano plot coefficients for the non–SARS-CoV-2 (**C**) and SARS-CoV-2 (**D**) humoral responses. Significance in the heatmaps is shown as FDR-corrected *P < 0.05, **P < 0.01, or ***P < 0.001.

To capture regional differences in this global cohort, high-income GBD region (United States and Canada) was compared with all other regions: Latin America/Caribbean (Brazil, Haiti, Peru, Puerto Rico), Southeast/East Asia (Thailand), South Asia (India), and sub-Saharan Africa (Botswana, South Africa, Uganda, Zimbabwe). Among COVID-19^+^ participants, no humoral immune differences were noted between the high-income and other GBD regions (though there was a trend toward higher antibody-specific FcγRIIA binding capacity in individuals from a high-income GBD region) (Figure 8, B and D). Among COVID-19^–^ participants, high-income GBD region was associated with generally higher antibodies and FcγR binding capacity for all non–SARS-CoV-2 antigens except HSV-1 and HSV-2 (Figure 8, A and C). High-income GBD region participants also tended to have higher EBV, CMV, RSV, influenza HA, and pneumococcal responses.

### Cigarette smoking and substance use.

There were no differences in the SARS-CoV-2 humoral immune repertoire related to current or former cigarette smoking or substance use history among COVID-19^+^ participants (Supplemental Figures 10 and 11). Among COVID-19^–^ participants, no consistent trends were observed. IgA1 responses tended to be higher in those with current or former cigarette smoking and substance use. Some CMV-, EBV-, and HIV-specific responses were higher with current or former substance use.

### HIV viremia.

**Figure 9:**
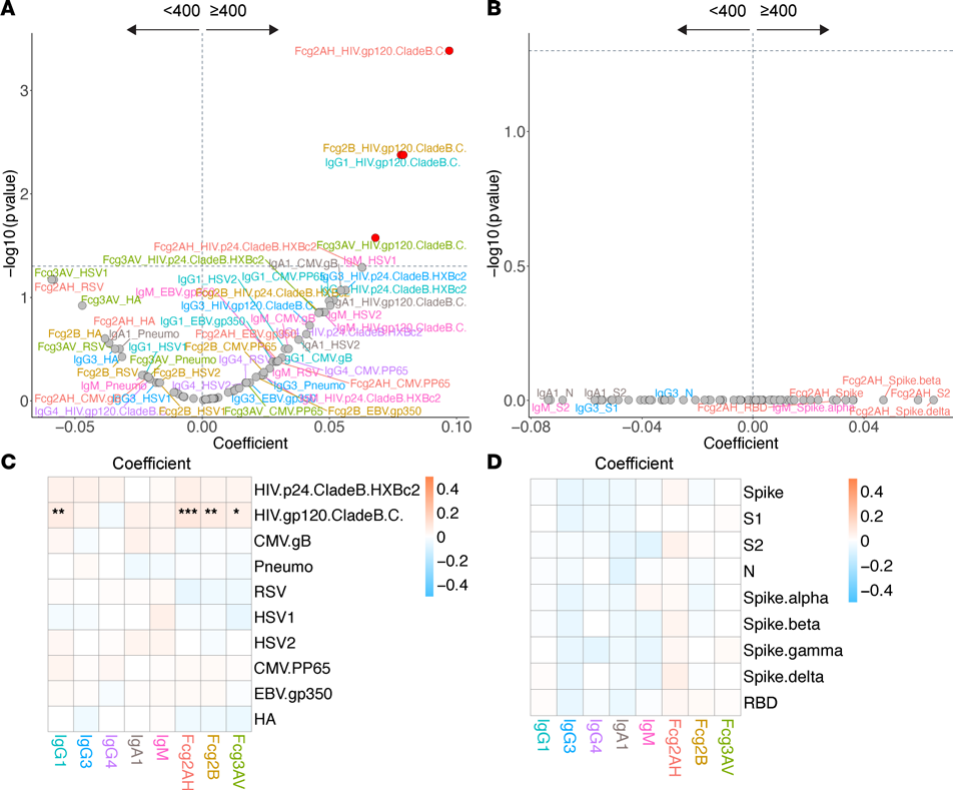
Volcano plots and heatmaps of effect of HIV viremia on the humoral immune repertoire. (**A** and **B**) Adjusted volcano plots of effect of HIV viremia (≥400 vs. <400 copies/mL) on the non–SARS-CoV-2 humoral repertoire (**A**) among the COVID-19^–^ cohort and SARS-CoV-2 humoral repertoire (**B**) among the COVID-19^+^ cohort. (**C** and **D**) Respective heatmaps of the volcano plot coefficients for the non–SARS-CoV-2 (**C**) and SARS-CoV-2 (**D**) humoral responses. Significance in the heatmaps is shown as FDR-corrected *P <0.05, **P < 0.01, or ***P < 0.001.

In this predominantly (97%) ART-suppressed cohort, no differences in SARS-CoV-2 humoral responses were observed among viremic versus virally suppressed COVID-19^+^ participants (Figure 9, B and D). Among COVID-19^–^ participants, as expected, HIV gp120–specific IgG1 and antibody-specific FcγR binding capacity were higher among the participants with viremia (Figure 9, A and C). None of the non–HIV-related antigen responses varied in participants who experienced viremia versus those who were virally suppressed.

### Current and nadir CD4^+^ T cell counts.

**Figure 10:**
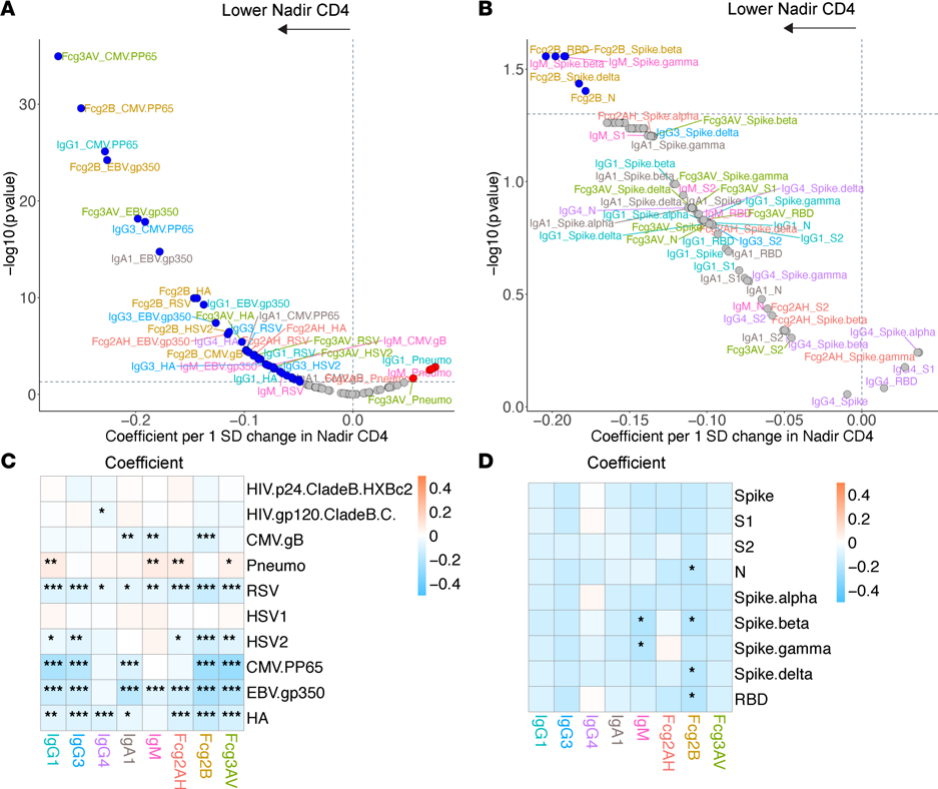
Volcano plots and heatmaps of effect of nadir CD4 on the humoral immune repertoire. (**A** and **B**) Adjusted volcano plots of effect of nadir CD4 on the non–SARS-CoV-2 humoral repertoire (**A**) among the COVID-19^–^ cohort and SARS-CoV-2 humoral repertoire (**B**) among the COVID-19^+^ cohort. Coefficients reflect the effect of a 1 SD increase in nadir CD4, which was *Z* scored for each participant from the ordinal scale of < 50, 50–199, 200–349, or ≥ 350 cells/mm^3^. (**C** and **D**) Respective heatmaps of the volcano plot coefficients for the non–SARS-CoV-2 (**C**) and SARS-CoV-2 (**D**) humoral responses. Significance in the heatmaps is shown as FDR-corrected *P < 0.05, **P < 0.01, or ***P < 0.001.

**Figure 11:**
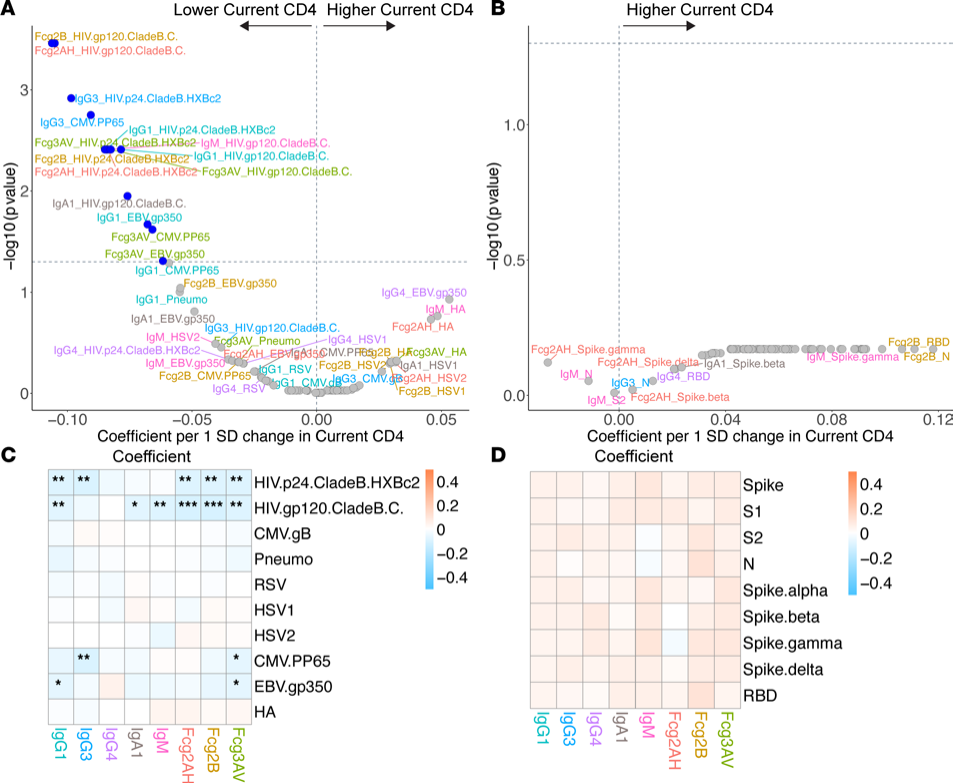
Volcano plots and heatmaps of effect of current CD4 on the humoral immune repertoire. (**A** and **B**) Adjusted volcano plots of effect of current CD4 (without nadir CD4 adjustment) on the non–SARS-CoV-2 humoral repertoire (**A**) among the COVID-19^–^ cohort and SARS-CoV-2 humoral repertoire (**B**) among COVID-19^+^ cohort. Coefficients reflect the effect of a 1 SD increase in current CD4 (cells/mm^3^), which was *Z* scored for each participant. (**C** and **D**) Respective heatmaps of the volcano plot coefficients for the non–SARS-CoV-2 (**C**) and SARS-CoV-2 (**D**) humoral responses. Significance in the heatmaps is shown as FDR-corrected *P < 0.05, **P < 0.01, or ***P < 0.001.

As current and nadir CD4^+^ T cell counts may act as different surrogates for persistent immune dysfunction, we assessed effects on the humoral repertoire through multivariate models with both parameters separately and with current CD4 adjusted by nadir CD4. In the COVID-19^+^ cohort, despite adequate CD4 recovery in the majority of participants with a median CD4 count of 609 (Q1–Q3, 466–817), lower nadir CD4 was associated with a significant humoral immune repertoire shift toward more IgM responses and a greater capacity for antibody-specific FcγRIIB binding (Figure 10, B and D). Current CD4, however, was not associated with any SARS-CoV-2 repertoire shift (Figure 11, B and D).

Among COVID-19^–^ participants, we observed clear associations between nadir CD4 and non–SARS-CoV-2 responses (Figure 10, A and C). The largest magnitude of associations appeared to be with higher EBV, CMV, and HSV-2 responses in those with lower nadir CD4. Those with lower nadir CD4^+^ T cell counts tended to have higher humoral responses to influenza HA and RSV but lower antibody responses to pneumococcus.

Modeling current CD4 without nadir CD4 adjustment revealed similar associations as those observed when modeling nadir CD4 alone but were generally less robust (Figure 11, A and C). Unlike nadir CD4, lower current CD4 was associated with heightened HIV-specific responses. In modeling adjusted for nadir CD4, lower current CD4 was associated with higher HIV p24 and gp120 responses and several CMV- and EBV-specific responses (Supplemental Figure 12). Together, these data reveal similar associations of current and nadir CD4 with the non–SARS-CoV-2 repertoire among COVID-19^–^ participants, but they also show a novel association of nadir CD4 with the SARS-CoV-2 repertoire among COVID-19^+^ participants not seen with current CD4.

### Supporting analyses.

Our primary analysis limited COVID-19 cases to those with positive antibody testing, and all other participants were included in the COVID-19^–^ cohort. Supporting analyses were performed in 2 ways to address those participants who were antibody negative but reported COVID-19–related adverse events. First, we included these 35 participants in the COVID-19^+^ cohort. Second, we excluded those cases from the COVID-19^–^ group. Inclusion and exclusion of these participants did not alter the study’s inferences (Supplemental Figures 13–36).

## Discussion

It is well established that PWH have impaired responses to some pathogens and immunizations. Over the course of the COVID-19 pandemic, it has also become increasingly recognized that PWH may face a higher risk of severe COVID-19 outcomes. Nevertheless, few studies have investigated the overall humoral immune repertoire in treated HIV and the host factors associated with specific responses. And while some studies have hinted at factors that may underlie the increased risk of COVID-19 severity in PWH, the underlying mechanisms remain unknown (22, 23, 25, 26, 42). Leveraging a large global cohort of ART-treated PWH to assess a comprehensive humoral immune profile in relation to COVID-19 and key host and HIV-specific factors, we addressed these gaps and present several important observations. First, among the COVID-19^–^ participants, different host factors were associated with overlapping but unique humoral repertoire changes — particularly sex, but also age and nadir CD4. These findings support prior immunization studies and provide a comprehensive humoral repertoire in treated PWH. Second, among the entire cohort, we observed that COVID-19 infection was modestly associated with CMV responses and potentially EBV responses; though we could not adjust for lifestyle differences, this finding may be clinically relevant, with higher humoral responses reflecting increased susceptibility to COVID-19 or being a consequence of persistent inflammation or viral reactivation thereafter. Most importantly, we identified unique SARS-CoV-2 humoral repertoire shifts independently associated with BMI and nadir CD4, but not other host factors. These findings suggest mechanisms underlying specific risk factors associated with worse COVID-19 outcomes among PWH.

### Factors associated with the humoral repertoire among COVID-19^–^ participants.

We provide a comprehensive humoral profile and the associated host and HIV-specific factors in treated PWH. Among COVID-19^–^ participants, we identified distinct but overlapping repertoire shifts independently associated with natal sex, age, region, cigarette smoking and substance use history, current and nadir CD4, and HIV viremia. Overall, factors associated with higher systemic immune activation such as older age, female sex, and lower nadir and current CD4 were associated with broadly higher antibody responses. This is consistent with the observation that systemic inflammation from proinflammatory cytokines such as IL-6 may stimulate higher antibody levels or types of antibody responses but also greater decays in those responses after antigen exposure (43, 44). While antibody levels are classically considered as surrogates for the intensity and frequency of antigen exposure, the magnitude of these antibody responses can be differentially reflective of the quality and coordination of the humoral or cellular responses based on the host or antigen (45, 46).

We observed a prominent shift toward higher non–SARS-CoV-2 antibody responses in women compared with men in the COVID-19^–^ cohort, consistent with previously described responses after immunization (16, 47). Highly functional IgG1 antibodies were higher for women across all non–SARS-CoV-2 antigens assessed. Women also tended to have higher EBV, CMV, HSV, and influenza HA humoral responses. This is likely a combination of genetic and sex hormone–related effects. For example, TLR7 and CD40L — encoded on the X chromosome — can escape X chromosome inactivation and likely contribute to improved antigen recognition, induction of IFN, and durable antibody responses (48). The antibody-promoting versus -suppressing effects of estrogen versus testosterone, respectively, has also been demonstrated (49). While women with HIV tend to have higher levels of select immune activation markers compared with men, which have been postulated to contribute to the excess rates of comorbidities seen in women, it is unclear if the observed differences in humoral responses by natal sex are related (50, 51). Importantly, these observations contrast with the lack of association between sex and the SARS-CoV-2 repertoire among COVID-19^+^ participants discussed below.

Among COVID-19^–^ participants, older age was associated with a humoral repertoire shift toward the herpesviruses as well as RSV and influenza. Higher antibody levels or greater antibody abilities to engage FcγR in this setting, however, may not reflect better control or protection. As people age with CMV, for example, upwards of 10% of the entire memory T cell repertoire is devoted to controlling CMV replication and disease and may be associated with increased morbidity and mortality in the general population and those with cell-mediated defects such as solid organ transplant recipients on immunosuppression and PWH (52–56). Immune activation and repeated CMV exposures can also drive T cells toward a more senescent, exhausted, and inflammatory phenotype (57, 58). As people age, there is a simultaneous increase in memory B cells and a decrease in immature B cells and antibody-antigen affinity, ultimately resulting in a decline in future response to antigen (59, 60). This may explain the observation that older participants had higher inhibitory/regulatory IgG4 antibodies to pneumococcus, as reduced opsonization and changes to antibody subclass have been reported in older adults (61, 62).

The differences in humoral responses based on GBD region in the COVID-19^–^ cohort were also remarkable. High-income GBD region participants tended to have broadly higher antibody responses and greater FcγR binding capacity to most non–SARS-CoV-2 antigens assessed. It is well established that immunization responses vary geographically, with lower responses found in developing countries (63, 64). These findings may suggest diminished humoral responses to antigens in non–high-income GBD regions due to a combination of endemic infections, local environmental conditions, and increased immune activation, which together may drive lymphoid fibrosis, reduced T follicular helper cells, and blunted neutralizing antibody responses (3, 65).

We identified notable non–SARS-CoV-2 repertoire shifts for current and nadir CD4. Those with lower current and nadir CD4 had generally higher humoral responses to EBV and CMV, while those with lower nadir CD4 had higher influenza but diminished pneumococcus responses. Lower current and nadir CD4 are associated with diminished responsiveness to immunization or pathogen exposure, and this holds true for nadir CD4 even in those with current CD4 > 350 cells/mm^3^ (9, 14, 43, 66). While multiple studies have shown a reduced response to influenza vaccination in those with lower nadir CD4, in our cohort, humoral responses to influenza HA tended to be higher in those with lower nadir CD4, which may be consistent with a prior study that assessed influenza vaccine–specific IgG titer at baseline prior to immunization (17, 43, 67). The COVID-19^–^ participants with lower nadir CD4 tend to have antibody profiles that reflect a mix of activating and inhibitory responses to control chronic viral infections like EBV or CMV or from prior resolved infections (68). Current CD4, without controlling for nadir CD4, tended to have less robust associations compared with nadir CD4. After controlling for nadir CD4, we noted few associations other than with HIV-specific responses among COVID-19^–^ participants. Despite a reliance on one’s current CD4 to risk stratify infection-associated risks in the clinical setting, our data suggest that nadir CD4, obtained years earlier and irrespective of current viral suppression or duration of ART, may be more immunologically relevant. Nadir CD4 plausibly reflects a critical setpoint with persistently higher degrees of systemic immune activation, inflammation, irreversible lymphoid fibrosis, and B and T cell defects, which can predict outcomes (4, 5, 7, 69).

### Effects of COVID-19 on humoral responses in the overall cohort.

We identified an association between COVID-19 and CMV pp65–specific IgG3 and FcγRIIA binding capacity among the overall cohort. In the general population, CMV seropositivity was related to an increased risk of COVID-19 acquisition and severity (32, 33). Importantly, we cannot rule out lifestyle differences as a confounder, as these could account for both higher CMV humoral responses and higher risk of COVID-19 in certain participants (32). Moreover, our study’s cross-sectional nature precludes understanding if a CMV-specific humoral repertoire pattern influenced incident COVID-19 or if CMV reactivation occurred in the context of COVID-19, leading to transient or more prolonged repertoire shifts. Indeed, asymptomatic CMV replication can be induced and caused by systemic inflammation (70, 71). In CMV-seropositive PWH, treatment with valganciclovir reduces T cell activation and multiple markers of immune activation (72). It is plausible that CMV engenders a higher baseline inflammatory milieu coupled with broad naive T cell depletion and expansion of late-differentiated and CD28^–^CD57^+^CD8^+^ T cells. These effects may contribute to immune remodeling that makes individuals — especially the immunocompromised or those with excess systemic inflammation — more susceptible to COVID-19 and severe disease.

In addition to CMV, there is some evidence of EBV-specific humoral responses associated with COVID-19 in the overall cohort. We observed a higher EBV gp350–specific antibody capacity to bind FcγRIIA in those with COVID-19, but this bordered statistical significance after FDR correction. EBV reactivation after COVID-19 is common in the general population and associated with increased mortality (73). Additionally, EBV reactivation may be associated with long COVID or PASC (34, 74). Interestingly, one study associated CMV seropositivity with a lower likelihood of neurocognitive long COVID symptoms, perhaps related to its own immunoregulatory pathways (34). While CMV and EBV are both herpesviruses, they have different anatomic locations, with EBV in the B cell follicle and CMV diversely localized (75). Since HIV appears to be associated with a higher risk of severe COVID-19 as well as long COVID or PASC, and CMV seropositivity has a relatively larger association with multiple comorbidities in PWH versus the general population, this might suggest that herpesviruses have a disproportionate impact on immune responses to and outcomes of acute COVID-19 and long COVID or PASC in PWH (34–36, 76).

### Humoral immune responses among COVID-19^+^ participants.

Despite a majority asymptomatic population, we observed that worse COVID-19 severity was associated with an amplification of SARS-CoV-2 humoral responses among COVID-19^+^ participants. This aligns with data from the general population that reveal higher or more durable antibody responses (though possibly less coordinated with T cell responses) in those with more severe COVID-19 (33, 77). We did not observe a marked association between most of the non–SARS-CoV-2 repertoire and COVID-19 severity. This is the first study to our knowledge in the context of HIV, while others in the general population have shown mixed associations of influenza, RSV, or other coronavirus responses as they relate to SARS-CoV-2 responses or their evolution after thereafter (33, 78).

The most striking associations among host factors and the SARS-CoV-2 repertoire among the COVID-19^+^ cohort were related to BMI and nadir CD4. While BMI had no effect on the non–SARS-CoV-2 repertoire, higher BMI was associated with a broad upregulation of SARS-CoV-2 responses and FcγR binding capacity. Obesity has been a consistent risk factor for severe COVID-19 in the general population, though the data from PWH has been more limited (23, 25). In obesity, adipocytes undergo hypertrophy and hyperplasia, leading to greater immune cell recruitment and secretion of proinflammatory cytokines like IL-6 and TNF-α (reviewed in ref. 79). This results in a chronic systemic inflammatory state that promotes proinflammatory macrophage infiltration and CD8^+^ cytotoxic T cells with simultaneous declines in CD4^+^ T helper cells and Treg populations and functions, contributing to an autoimmune-like phenotype and positive inflammatory feedback loop (80). While those with higher BMI had generally higher antibody responses (and studies have observed an association between broadly higher antibody responses and more severe COVID-19 or mortality), we observed N responses to be the highest in those with higher BMI, which has been associated with more severe COVID-19 and has been postulated to be due to a higher antigen burden or compromised spike-targeting humoral evolution (81, 82). Since there are roughly 1,000 copies of N compared with 100 copies of spike in each virus, N responses may also be the most sensitive (83). The pivotal question surrounding these amplified responses is whether the quantitative antibody changes simply track with antigen burden or reflect qualitative differences that may drive pathology. Obesity is associated with upregulation of the ACE2 receptor in adipose tissue and the lungs, leading to higher VLs, decreased viral clearance, and a more durable antigenic reservoir; increased ACE2 shedding in this setting could also contribute (84–88). Moreover, while most SARS-CoV-2 humoral responses were higher in those with higher BMIs, there were no differences in the inhibitory IgG4 and antibody capacity to bind FcγRIIB; in fact, IgG4 responses tended to be higher in those with lower BMIs. The observation that SARS-CoV-2–specific IgG4 and FcRIIB binding capacity were not broadly higher in those with higher BMIs and tended to reflect inhibitory or regulatory responses suggests that this BMI-associated humoral profile may contribute to a pathologic response (68).

A unique SARS-CoV-2 humoral response was also associated with lower nadir CD4 among the COVID-19^+^ cohort. Most studies of COVID-19 in PWH have associated lower current CD4 with worse outcomes; nadir CD4 has seldom been assessed (22, 23, 26). We observed lower nadir CD4 was associated with higher SARS-CoV-2–specific IgM responses and antibody-specific FcγRIIB binding capacity. IgM responses were most strongly affected by nadir CD4, and this may suggest less effective antibody class switching. While CD4^+^ T cells are involved in class switching, lower nadir CD4 is associated with both the degree of CD4 recovery on ART and the functional response thereafter (2, 89). Our data point to permanent functional alterations, which may irreparably affect the ability of CD4^+^ T cells to assist with de novo responses to a novel pathogen. The humoral profile we identified could also be more extrafollicular and less effective. In the general population, acute COVID-19 is associated with germinal center loss in lymph nodes and the spleen, leading to an accumulation of non–germinal center–derived activated B cells associated with higher levels of inflammation (90). PWH have significant lymphoid fibrosis and inflammation associated with current and nadir CD4 (5). Therefore, the combination of germinal center pathology from both chronic HIV and acute COVID-19 could lead to a unique extrafollicular response that lacks high-affinity B cell formation, adequate viral clearance, and the development of long-lived memory B cells.

### Differences in host factors associated with humoral repertoires by COVID-19.

While multiple host factors were associated with unique non–SARS-CoV-2 humoral responses among COVID-19^–^ participants, only nadir CD4 and BMI were associated with SARS-CoV-2 profile shifts in the COVID-19^+^ cohort. We did not observe any SARS-CoV-2 humoral profile differences by current CD4, perhaps due to our cohort’s inclusion of PWH on stable ART, leading to a narrower range of current CD4, and due to REPRIEVE’s exclusion of those with current CD4 < 100 cells/mm^3^. Other studies have associated HIV viremia with worse COVID-19 outcomes; the lack of association between viremia and SARS-CoV-2 responses is likely due to REPRIEVE’s enrollment of predominantly virally suppressed participants (23, 91, 92). The lack of association of natal sex and humoral responses among the COVID-19^+^ PWH is notable and stands in contrast to what we observed in the COVID-19^–^ cohort. Several studies have shown sex differences in COVID-19 outcomes in the general population, with higher levels of inflammatory markers in men as a prominent feature impacting severe disease (29, 30). The association of sex with severe disease, however, declined over the age spectrum, suggesting that sex hormone alteration may be a relevant factor.

### Strengths and limitations.

The study’s strengths include its large size, global nature, and inclusion of a large proportion of non-White and female participants. We incorporated clinical data with potentially novel assays to provide comprehensive humoral immune phenotyping within a modern population of well-controlled PWH. Nevertheless, this study does have several limitations. First, the study’s cross-sectional nature limits our ability to assess dynamic humoral repertoire changes over time in relation to COVID-19. Second, we were unable to control for statin use, given the ongoing nature of the trial. Nonetheless, the differences in randomization to statin therapy between the COVID-19^+^ and COVID-19^–^ groups were less than 5%, arguing against significant confounding. Additionally, while we had a global and diverse study population, there was a relatively narrow age range (close to 90% were ages 40–60), which may limit our ability to detect key age-related differences. Since most participants were virally suppressed, our ability to detect effects of HIV viremia on humoral responses was also limited. In terms of COVID-19 ascertainment, we defined COVID-19 based on antibody status, though sensitivity analysis with inclusion or exclusion of potential clinical cases maintained similar inferences. Since most participants had asymptomatic or subclinical infection, we were unable to more thoroughly evaluate host factors associated with severe COVID-19 and associated SARS-CoV-2 and non–SARS-CoV-2 responses. Finally, we used the standardized DAIDS adverse event grading scale to assess COVID-19 severity; further insights may be provided with more granular severity scales (e.g., World Health Organization).

### Conclusion.

In summary, we provide a comprehensive overview of the humoral immune repertoire in treated PWH as well as host and HIV-specific factors that are associated with differential humoral shifts, both within and between the COVID-19^+^ and COVID-19^–^ participants. We demonstrated a possible association between COVID-19 infection and higher EBV- and CMV-specific humoral responses in the overall cohort, and this association may reflect an increased susceptibility to COVID-19 or be indicative of a consequence of persistent inflammation or reactivation after infection — both critical in the pathogenesis of acute COVID-19 and long COVID or PASC. Finally, in individuals with COVID-19, we identified a striking inflammatory-like amplification of SARS-CoV-2 responses with higher BMI and a unique extrafollicular and poorly functioning SARS-CoV-2 repertoire shift toward higher IgM and FcγRIIB binding capacity in those with lower nadir CD4, but we did not see major influences of other host factors on the SARS-CoV-2 repertoire. These distinctive profiles may suggest humorally mediated mechanisms underlying worse COVID-19 outcomes in this setting.

## Methods

### RBD ELISA assay.

The Alter Lab developed a SARS-CoV-2–specific ELISA, allowing for the detection of RBD-specific IgG and IgA in an automated manner, and has been previously evaluated against EUA-approved ELISAs with > 99.5% specificity (93, 94). The 384-well ELISA robot-automated platform utilized plates coated with 0.5 μg/mL of RBD for 1 hour at 37°C in a bicarbonate buffer. After plate washing, plasma samples were added at 1:100 dilution, in duplicate, for 1 hour at 37°C. Plates were washed and detected with a secondary anti–IgG HRP–coupled detection antibody (Bethyl Laboratories, A80-104P) for 1 hour. Plates were washed again with addition of colorimetric detector (TMB; Thermo Fisher Scientific) for 5 minutes. The reaction was stopped, and the absorbance was acquired at 450/570 nm. Conversion from OD values to μg/mL concentrations were performed on ELISA plates via twelve 2-fold dilution curves, starting at 625 ng/mL, of a SARS-CoV-2 RBD-specific monoclonal IgG1 (Alter Lab, clone CR3022). The sample concentration was then interpolated from the standard curve (93). A sample was considered positive if it equaled the mean of the negative-control wells on each respective plate plus 5 times the SD of the concentration from negative plasma samples. Background-corrected concentrations were divided by the cutoff to generate signal-to-cutoff (S/CO) ratios. Lab members were blinded until all data were collected and analyzed.

### Antigens.

The Luminex assay assessed the following SARS-CoV-2 antigens including: spike WT (LakePharma), spike α variant (LakePharma), spike β variant (LakePharma), spike γ variant (LakePharma), spike δ variant (Saphire Lab), RBD (WT) (Aaron Schmidt Lab), S1 (WT) (Sino Biological), S2 (WT) (Sino Biological), and N (WT) (Aalto Bio Reagents Ltd). The Luminex assay assessed the following non–SARS-CoV-2 antigens: HIV p24 clade B (Immune Technology Corp.), HIV gp120 clade B/C (Immune Technology Corp.), CMV gB (Sino Biological), CMV pp65 (Sino Biological), EBV gp350 (Immune Technology Corp.), HSV-1 (Immune Technology Corp.), HSV-2 (Immune Technology Corp), influenza HA (Immune Technology Corp), pneumococcus (Massachusetts General Hospital Pharmacy), and RSV (Sino Biological).

### Luminex array for IgG subclass, isotype, and FcγR binding.

SARS-CoV-2 and non–SARS-CoV-2 antigen-specific antibody subclass, isotype, and FcγR binding capacity were assessed on a custom Luminex array run in singlicate in a batched manner (95).

Fluorescently coded microspheres captured all antigen specificities concurrently and profiled (a) the isotype and subclass distributions in an antigen-specific manner and (b) the effector capacity of each antigen specificity by assessing the ability of antigen-specific antibodies to interact with Fc receptors. The specific target antigen, a positive control antigen (influenza HA), and a negative control antigen (Ebola virus group, Mayflower Bioscience) were covalently coupled to magnetic Luminex beads (Luminex Corp.) via N-hydroxysuccinimide–ester (NHS-ester) linkages with Sulfo-NHS and EDC (Thermo Fisher Scientific) for 2 hours at room temperature (RT). Pneumococcal polysaccharide vaccine was modified by COOH-4-(4,6-dimethoxy[1,3,5]triazin-2-yl)-4-methyl-morpholinium (DMTMM) before coupling to Luminex beads (96). Dilution curves on pooled cohort samples were created to determine dilutions in the linear range for individual detection reagents. Beads were incubated with appropriate diluted (in PBS) serum at 4°C overnight (1:100 for IgG1, IgG3, IgG4, IgA1, and IgM; 1:1,000 for FcRIIA, FcγRIIB, and FcγRIIIA). Unbound antibodies were washed, and bound antigen-specific antibodies were probed with polyclonal PE–conjugated antibody (Southern Biotech; anti-IgG1, 9052-09; anti-IgG3, 9210-09; anti-IgG4, 9200-09; anti-IgM, 9020-09; anti-IgA1, 9130-09) or tetramerized recombinant Fc receptors (Duke Protein Production Facility) for 1 hour at RT. FcγRs were biotinylated with a BirA500 kit (Avidity LLC) beforehand. The biotinylated FcγRs were fluorescently tagged using streptavidin-PE (Agilent) and incubated with antigen-specific antibodies (Agilent, PJ31S). After the incubation, excess secondary reagent was washed. Relative concentrations per antigen were measured on an IQue analyzer (IntelliCyt). Data were reported as the median fluorescence intensity (MFI) of phycoerythrin (PE) for a specific bead channel.

### Statistics.

Univariate analysis was performed to assess the relationships between antibody responses and the following covariates: COVID-19 RBD IgG/IgA status, COVID-19 severity, age, sex, GBD region, BMI, history of current/former smoking, history of current/former substance use, current CD4, nadir CD4, and HIV VL. Violin plots were evaluated via 2-sided Wilcoxon rank-sum test. Univariate heatmaps reflected the results from unadjusted linear regression modeling, with the antibody or FcγR as the dependent outcome and the covariates as predictors. All ordinal and continuous variables, including the antibody and FcγR values, were *Z* scored by subtracting the cohort mean from the observed value and dividing by the respective cohort SD for interpretability and to satisfy model assumptions. This allowed for an interpretation of a SD increase in the predictor of interest associating with a coefficient increase in the *Z* scored antibody response.

Based on univariate findings and a priori assumptions, multivariate linear regression models were constructed for each antibody response as the dependent variable and for each predictor of interest as the main independent variable. Models were adjusted for age, sex, GBD region, nadir CD4, and HIV VL. Current CD4 as a main predictor was assessed with and without adjustment for nadir CD4. As a main predictor, GBD region was considered as binary (high-income vs. others); as a covariate in other models, it was included as a categorical variable. HIV VL was considered as a binary (<400 or ≥400 copies/mL). Volcano plots and heatmaps were used to visualize the coefficient and *P* values.

The primary analysis was performed with COVID-19 defined as a positive RBD IgG and/or IgA (*n* = 283). Supporting analyses were performed with those reporting an adverse event from COVID-19 as either excluded from the COVID-19^–^ cohort or included in the COVID-19^+^ cohort (*n* = 318).

There were limited missing data; nadir CD4 values were missing in 2.6% (*n* = 63/2,464), and HIV VL was missing in 0.9% (*n* = 22/2,464) of participants. Those with missing data for the relevant variables were excluded from analysis.

All statistical tests were 2 tailed. An α level of 0.05 was used to guide statistical inference, either uncorrected as in the univariate models or FDR-corrected as above in the multivariate linear regression models (41). Analyses were performed with R (version 4.1.0).

### Study approval.

Each clinical research site obtained IRB/ethics committee approval and any other applicable regulatory entity approvals. Participants were provided with study information, including discussion of risks and benefits, and signed the approved declaration of informed consent.

## Author contributions

SKG and GA conceived the study. KVF, MVZ, SM, ESF, MRD, FG, RC, ANS, CJF, CM, JAA, JC, PSD, HJR, and SKG managed the parent trial. JSLL, SS, and BJD performed the laboratory measurements under the guidance of GA. SRS, WJ, GA, and SKG designed the analysis. WJ performed the statistical analysis. HJR and DAL advised on the statistical analysis. SRS drafted the manuscript. All authors provided revisions and edits in the drafting of the manuscript. Order of coauthors was determined by agreement of coauthors.

## Supplementary Material

Supplemental data

## Figures and Tables

**Table 1:**
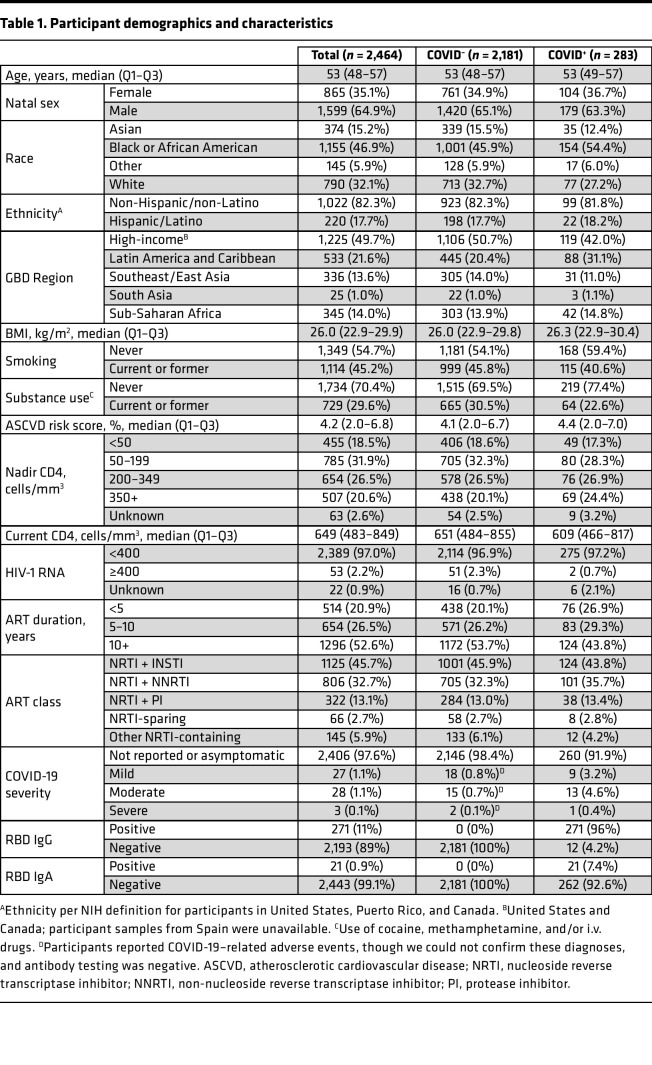
Participant demographics and characteristics

## Version 1 02/20/2023

In-Press Preview

## Version 2 03/08/2023

Electronic publication
