# Supplementary material for: Effect of host factors and COVID-19 infection on the humoral immune repertoire in treated HIV
Source: JCI Insight. 2023 Mar 8;8(5):e166848. doi: 10.1172/jci.insight.166848 (PMC10077482; doi:10.1172/jci.insight.166848)
Supplement: Supplemental data [file jciinsight-8-166848-s139.pdf]

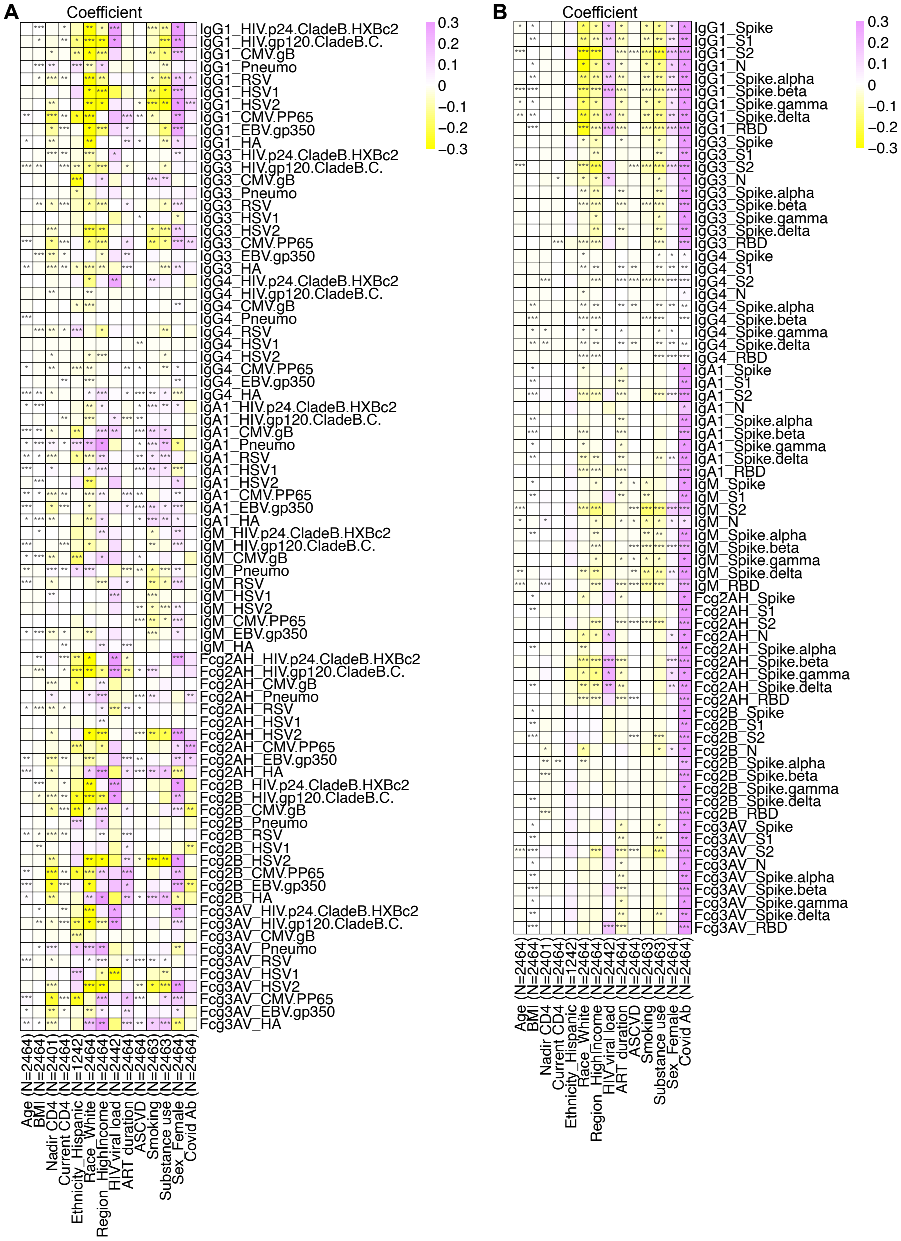

Figure S1. Univariate heat maps, all participants

Univariate heat maps relating COVID-19 status, COVID-19 severity, and host factors to the (A) non-SARS-CoV-2 and (B) SARS-CoV-2 humoral repertoire in all participants. Coefficients derived from unadjusted linear regression modeling. Significance in the heat maps is depicted as non-FDR-corrected P<0.05 (*), P<0.01 (**), or P<0.001 (***).

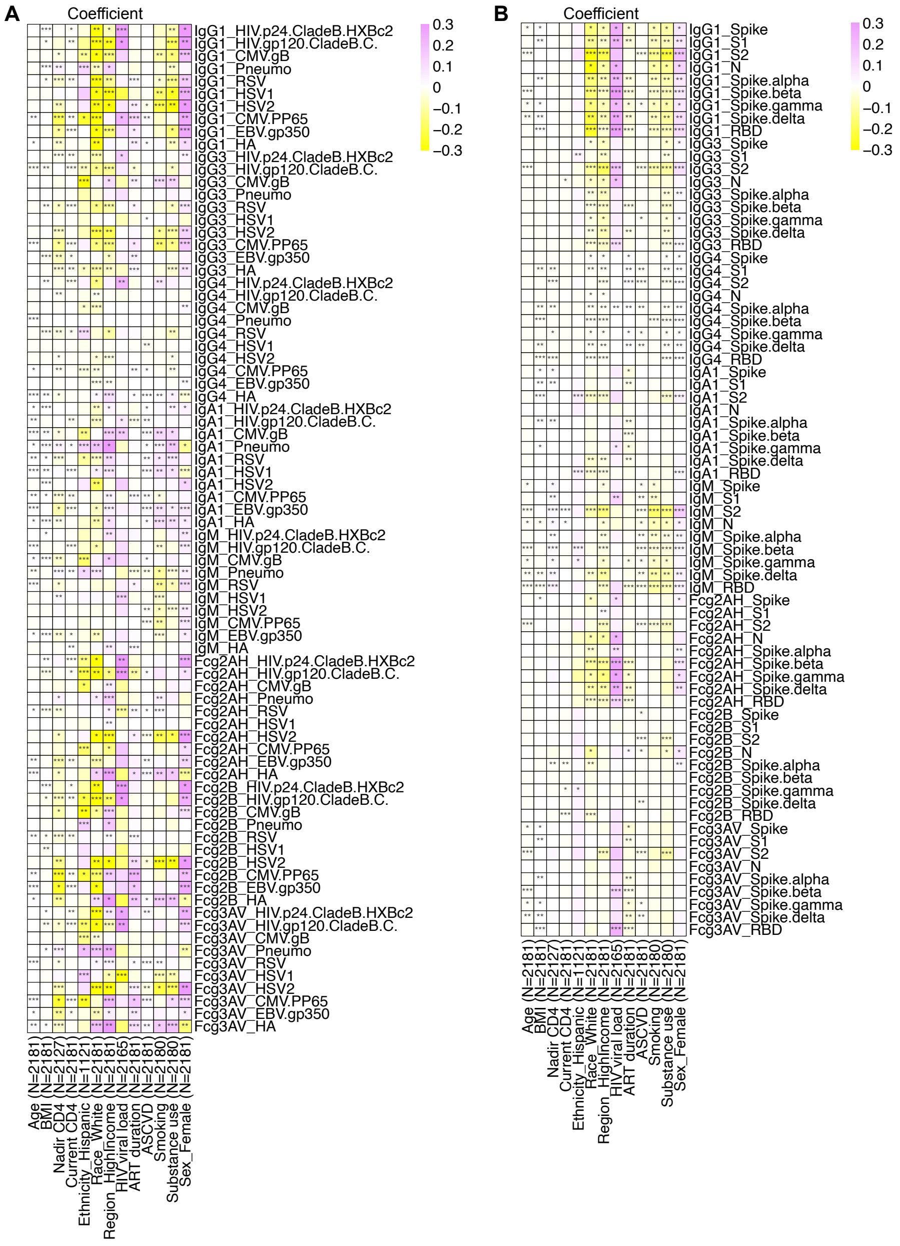

Figure S2. Univariate heat maps, COVID-negative participants Univariate heat maps relating host factors to the (A) non-SARS-CoV-2 and (B) SARS-CoV-2 humoral repertoire among COVID-negative participants. Coefficients derived from unadjusted linear regression modeling. Significance in the heat maps is depicted as non-FDR-corrected P<0.05 (*), P<0.01 (**), or P<0.001 (***).

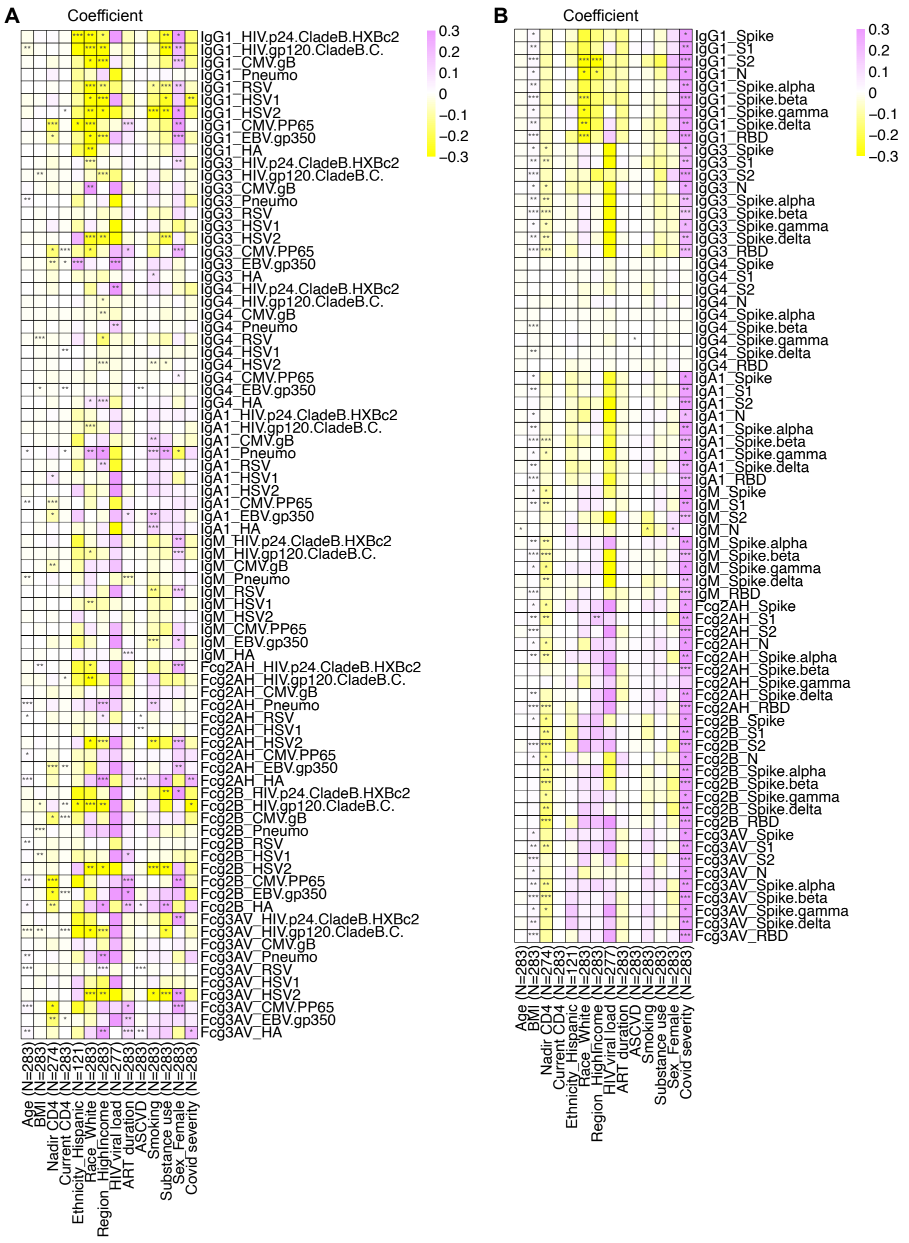

Figure S3. Univariate heat maps, COVID-positive participants Univariate heat maps relating COVID-19 severity and host factors to the (A) non-SARS-CoV-2 and (B) SARS-CoV-2 humoral repertoire in COVID-positive participants. Coefficients derived from unadjusted linear regression modeling. Significance in the heat maps is depicted as non-FDR-corrected P<0.05 (*), P<0.01 (**), or P<0.001 (***).

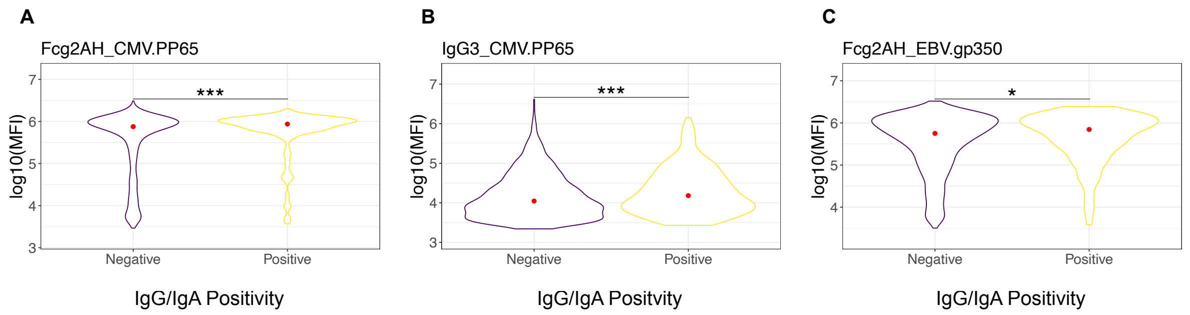

Figure S4. Univariate violin plots of effect of SARS-CoV-2 RBD IgG/IgA positivity on the humoral immune repertoire among all participants.

Violin plots of effect of SARS-CoV-2 RBD IgG/IgA positivity on **(A)** CMV PP65 FcγRIIA, **(B)** CMV PP65 IgG3, **(C)** EBV gp350 FcγRIIA. Significance testing was performed via Wilcoxon rank-sum test and is shown as P<0.05 (*), P<0.01 (**), or P<0.001 (***).

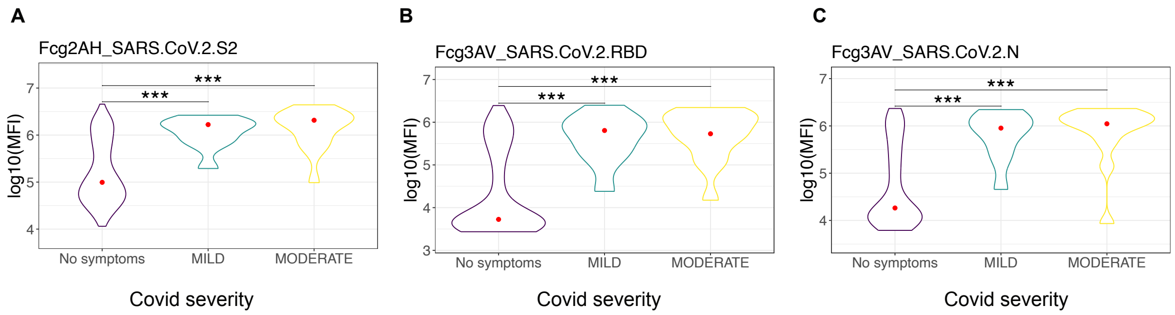

Figure S5. Univariate violin plots of effect of COVID-19 severity on the humoral immune repertoire among COVID-positive participants.

Violin plots of effect of COVID-19 severity on SARS-CoV-2 **(A)** Spike S2 subunit FcγRIIA **(B)** Receptor-binding domain (RBD) FcγRIIIA **(C)** Nucleocapsid FcγRIIIA. Significance testing was performed via Wilcoxon rank-sum test and is shown as $P<0.05$ (*), $P<0.01$ (**), or $P<0.001$ (***).

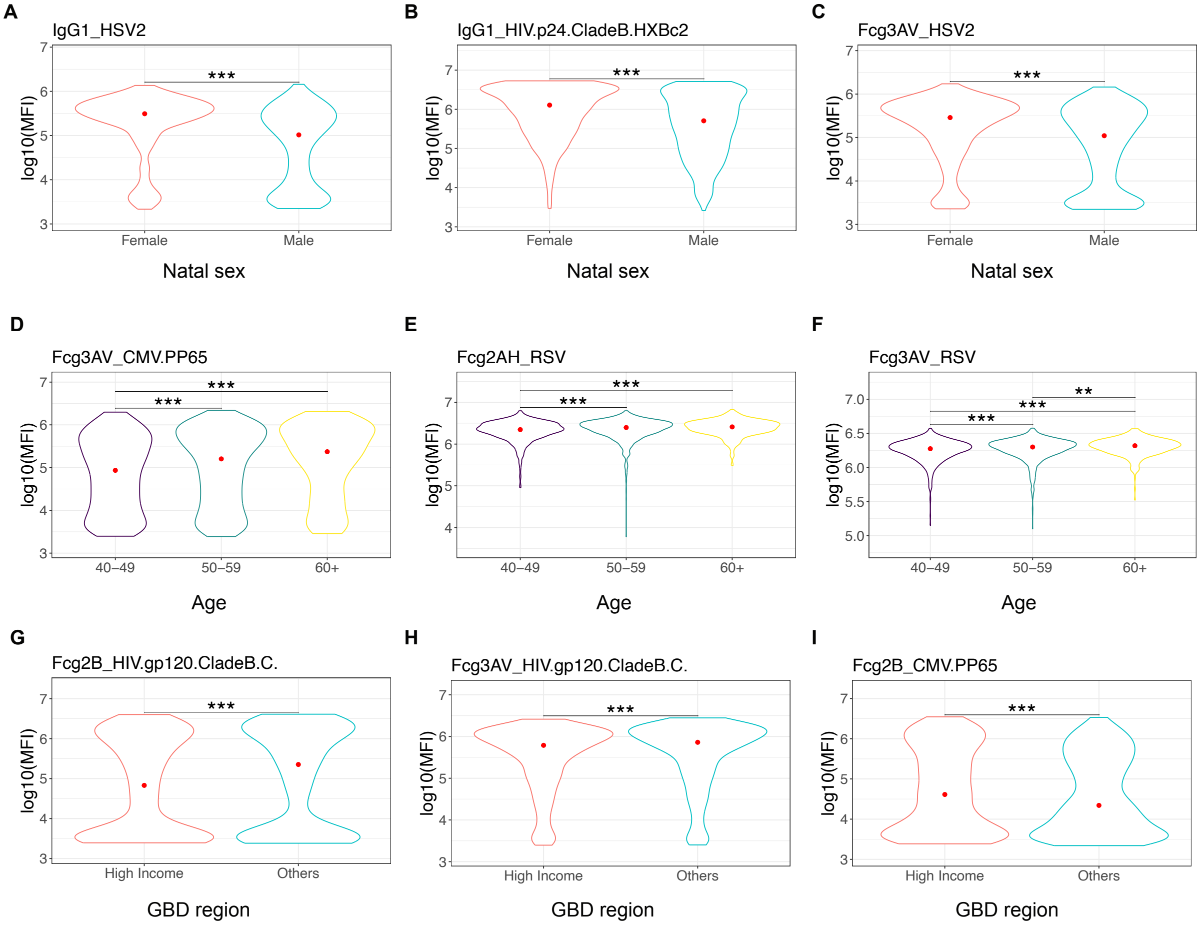

Figure S6. Univariate violin plots of effect of natal sex, age, and GBD on the humoral immune repertoire among COVID-negative participants.
Violin plots of effect of natal sex on **(A)** HSV-2 IgG1, **(B)** HIV p24 IgG1, **(C)** HSV-2 FcγRIIIA. Violin plots of effect of age on **(D)** CMV PP65 FcγRIIIA, **(E)** RSV FcγRIIA, **(F)** RSV FcγRIIIA. Violin plots of effect of GBD region on **(G)** HIV gp120 FcγRIIB, **(H)** HIV gp120 FcγRIIIA, **(I)** CMV gB FcγRIIB. Significance testing was performed via Wilcoxon rank-sum test and is shown as P<0.05 (*), P<0.01 (**), or P<0.001 (***).

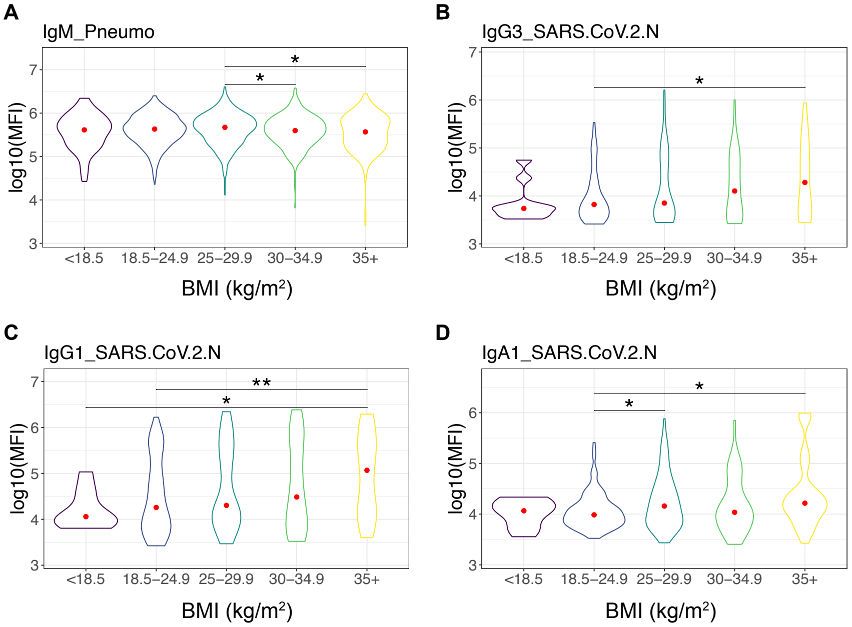

Figure S7. Univariate violin plots of effect of BMI on the humoral immune repertoire among COVID-negative and COVID-positive participants.
Violin plots of effect of BMI on **(A)** pneumococcus IgM among COVID-negative participants. Violin plots of effect of BMI on SARS-CoV-2 **(B)** Nucleocapsid IgG3, **(C)** Nucleocapsid IgG1, **(D)** Nucleocapsid IgA1 among COVID-positive participants. Significance testing was performed via Wilcoxon rank-sum test and is shown as P<0.05 (*), P<0.01 (**), or P<0.001 (***).

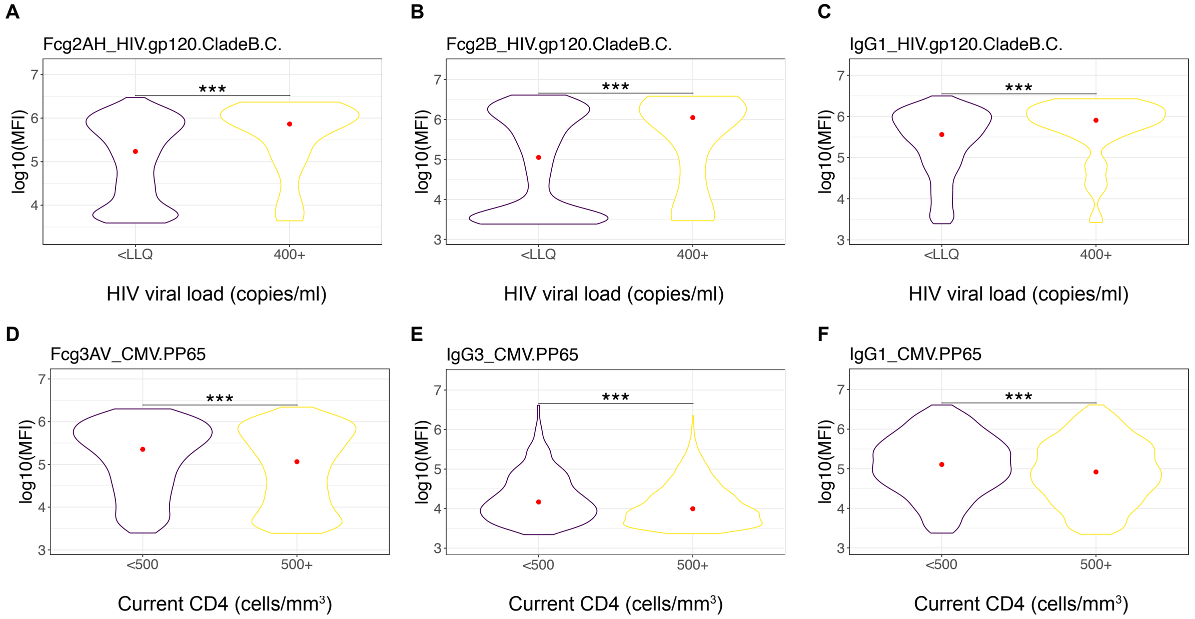

Figure S8. Univariate violin plots of effect of plasma HIV-1 RNA and current CD4 on the humoral immune repertoire among COVID-negative participants.
Violin plots of effect of HIV-1 RNA on **(A)** HIV gp120 FcγRIIA, **(B)** HIV gp120 FcγRIIB, and **(C)** HIV gp120 IgG1 among COVID-negative participants. Violin plots of effect of current CD4 on **(D)** CMV pp65 FcγRIIIA, **(E)** CMV PP65 IgG3, **(F)** CMV PP65 IgG1 among COVID-negative participants. Significance testing was performed via Wilcoxon rank-sum test and is shown as P<0.05 (*), P<0.01 (**), or P<0.001 (***).

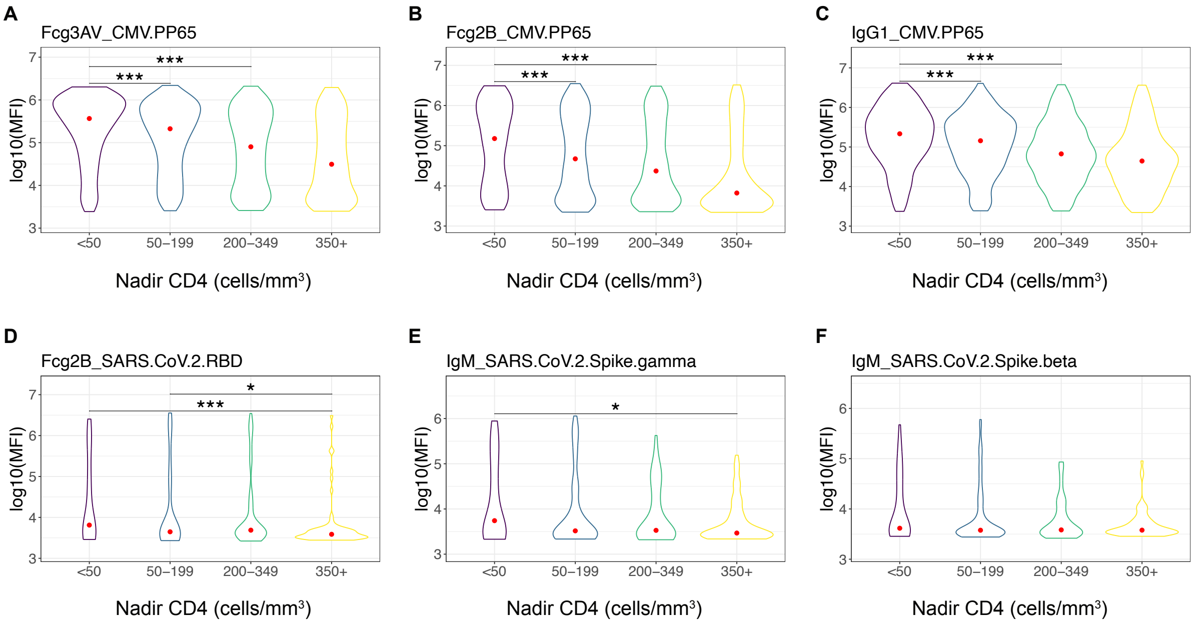

Figure S9. Univariate violin plots of effect of nadir CD4 on the humoral immune repertoire among COVID-negative and COVID-positive participants.

Violin plots of effect of nadir CD4 on **(A)** CMV PP65 FcγRIIIA, **(B)** CMV PP65 FcγRIIB, and **(C)** CMV PP65 IgG1 among COVID-negative participants. Violin plots of effect of nadir CD4 on SARS-CoV-2 **(D)** Receptor binding domain (RBD) FcγRIIB, **(E)** Spike gamma IgM, **(F)** Spike beta IgM among COVID-positive participants. Significance testing was performed via Wilcoxon rank-sum test and is shown as P<0.05 (*), P<0.01 (**), or P<0.001 (***).

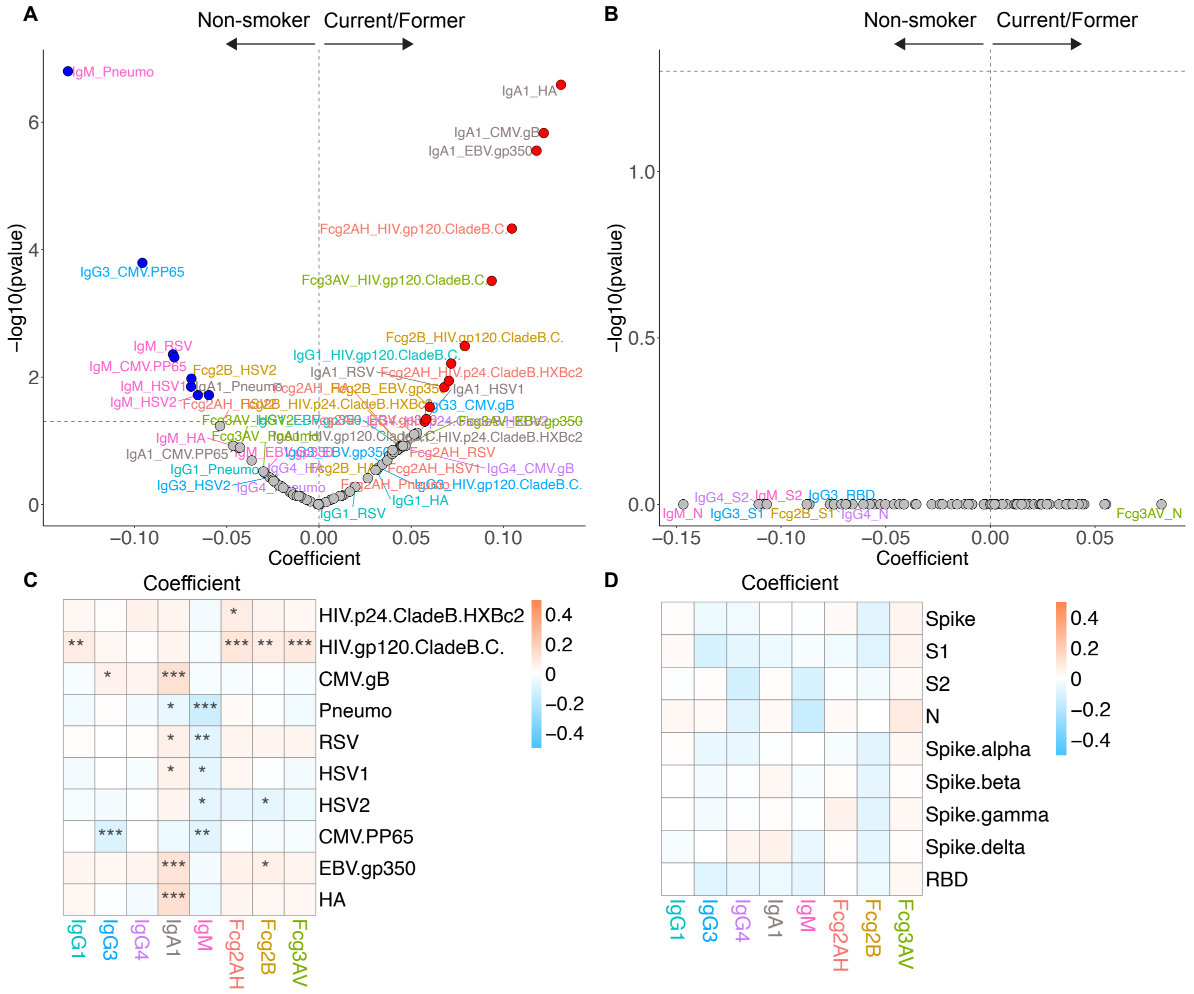

Figure S10. Volcano plots and heat maps of effect of smoking on the humoral immune repertoire. Volcano plot of effect of smoking on the **(A)** non-SARS-CoV-2 humoral repertoire among the COVID-negative cohort and **(B)** SARS-CoV-2 humoral repertoire among the COVID-positive cohort. Volcano plots constructed from linear regression models, adjusted for age, sex, GBD region, nadir CD4, and HIV viral load, with horizontal dashed line of significance displayed for FDR-corrected P=0.05. Responses higher in current/former smoking fall toward the right of the vertical dashed line, while responses higher in never-smoking fall toward the left of the vertical dashed line. Respective heat maps of the volcano plot coefficients for the **(C)** non-SARS-CoV-2 and **(D)** SARS-CoV-2 humoral responses. Coefficients >0 are higher in current/former smoking, while coefficients <0 are higher in never-smoking. Significance in the heat maps is shown as FDR-corrected P<0.05 (*), P<0.01 (**), or P<0.001 (***). Specific antibody isotype, subclass, and Fc-receptor responses are color-coded between the volcano plots and heat maps.

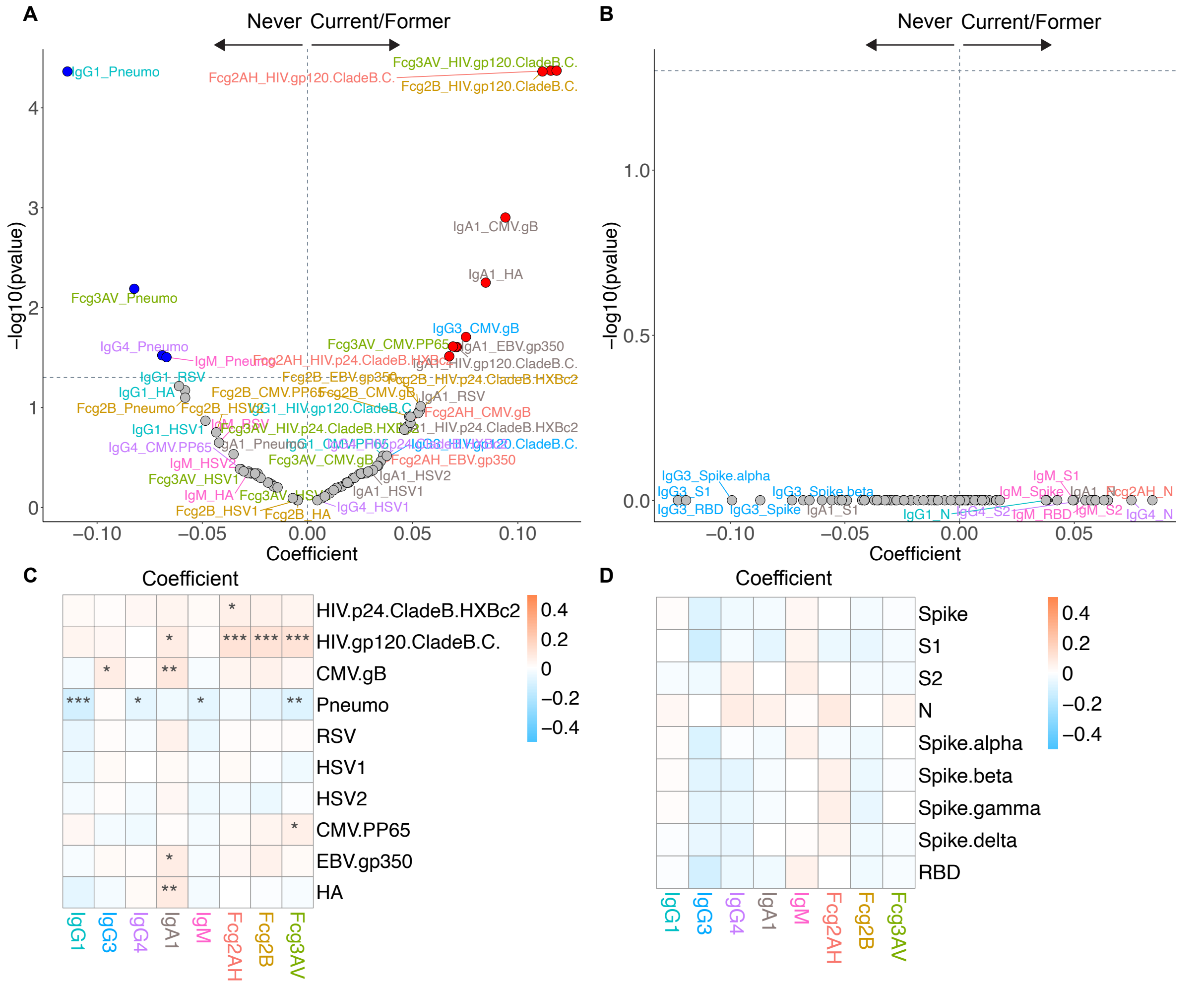

Figure S11. Volcano plots and heat maps of effect of substance use on the humoral immune repertoire. Volcano plot of effect of substance use on the **(A)** non-SARS-CoV-2 humoral repertoire among the COVID-negative cohort and **(B)** SARS-CoV-2 humoral repertoire among the COVID-positive cohort. Volcano plots constructed from linear regression models, adjusted for age, sex, GBD region, nadir CD4, and HIV viral load, with horizontal dashed line of significance displayed for FDR-corrected P=0.05. Responses higher in current/former substance use fall toward the right of the vertical dashed line, while responses higher in never-substance use fall toward the left of the vertical dashed line. Respective heat maps of the volcano plot coefficients for the **(C)** non-SARS-CoV-2 and **(D)** SARS-CoV-2 humoral responses. Coefficients >0 are higher in current/former substance use, while coefficients <0 are higher in never-substance use. Significance in the heat maps is shown as FDR-corrected P<0.05 (*), P<0.01 (**), or P<0.001 (***). Specific antibody isotype, subclass, and Fc-receptor responses are color-coded between the volcano plots and heat maps.

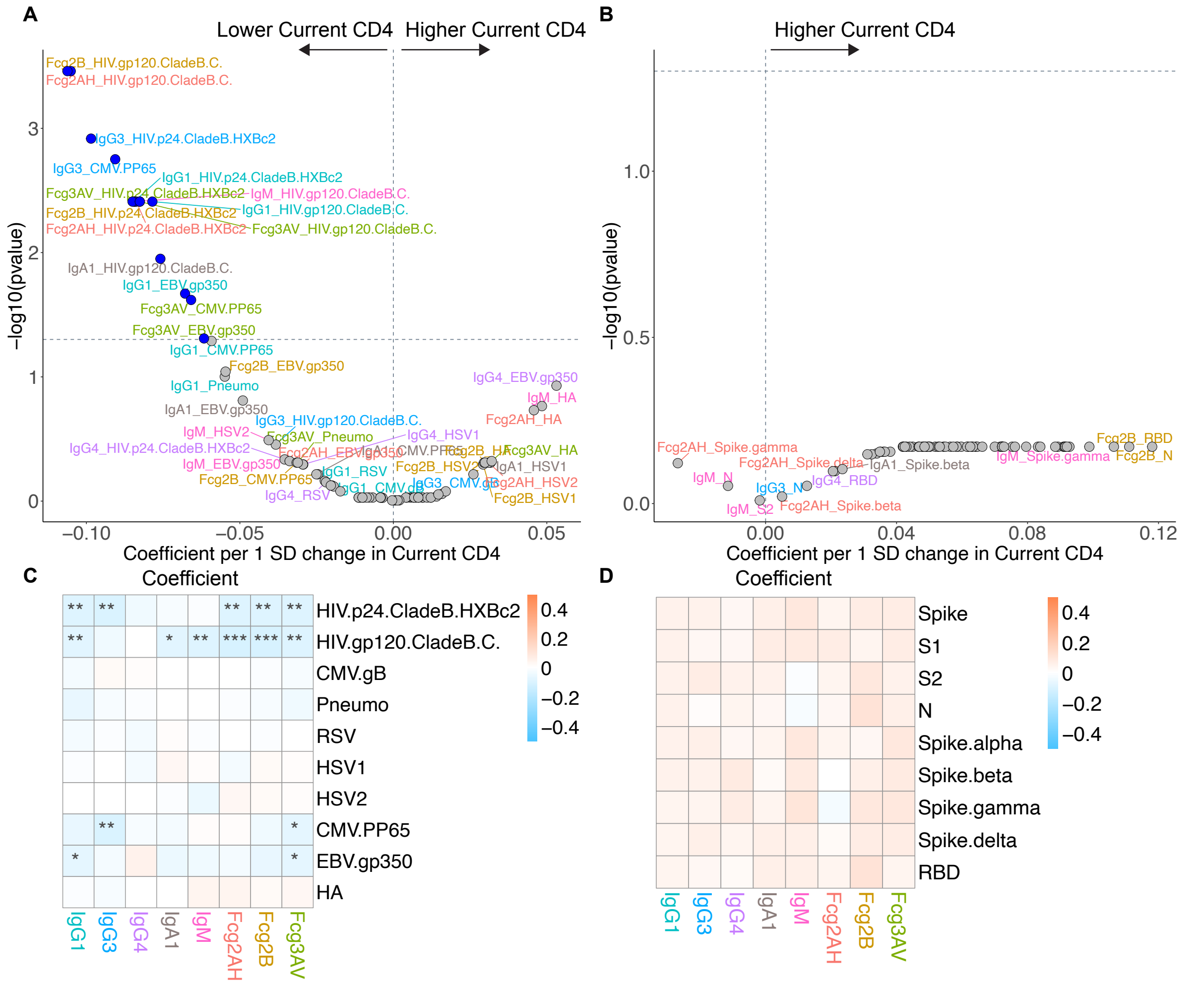

Figure S12. Volcano plots and heat maps of effect of current CD4 adjusted for nadir CD4 on the humoral immune repertoire. Volcano plot of effect of current CD4 adjusted for nadir CD4 on the **(A)** non-SARS-CoV-2 humoral repertoire among the COVID-negative cohort and **(B)** SARS-CoV-2 humoral repertoire among COVID-positive cohort. Volcano plots constructed from linear regression models, adjusted for age, sex, GBD region, nadir CD4, and HIV viral load, with horizontal dashed line of significance displayed for FDR-corrected P=0.05. Coefficients reflect the effect of a 1 SD increase in current CD4 (cells/mm^3), which was z-scored for each participant. Responses higher with higher current CD4 fall toward the right of the vertical dashed line, while responses higher with lower current CD4 fall toward the left of the vertical dashed line. Respective heat maps of the volcano plot coefficients for the **(C)** non-SARS-CoV-2 and **(D)** SARS-CoV-2 humoral responses. Coefficients >0 are higher with higher current CD4, while coefficients <0 are higher with lower current CD4. Significance in the heat maps is shown as FDR-corrected P<0.05 (*), P<0.01 (**), or P<0.001 (***). Specific antibody isotype, subclass, and Fc-receptor responses are color-coded between the volcano plots and heat maps.

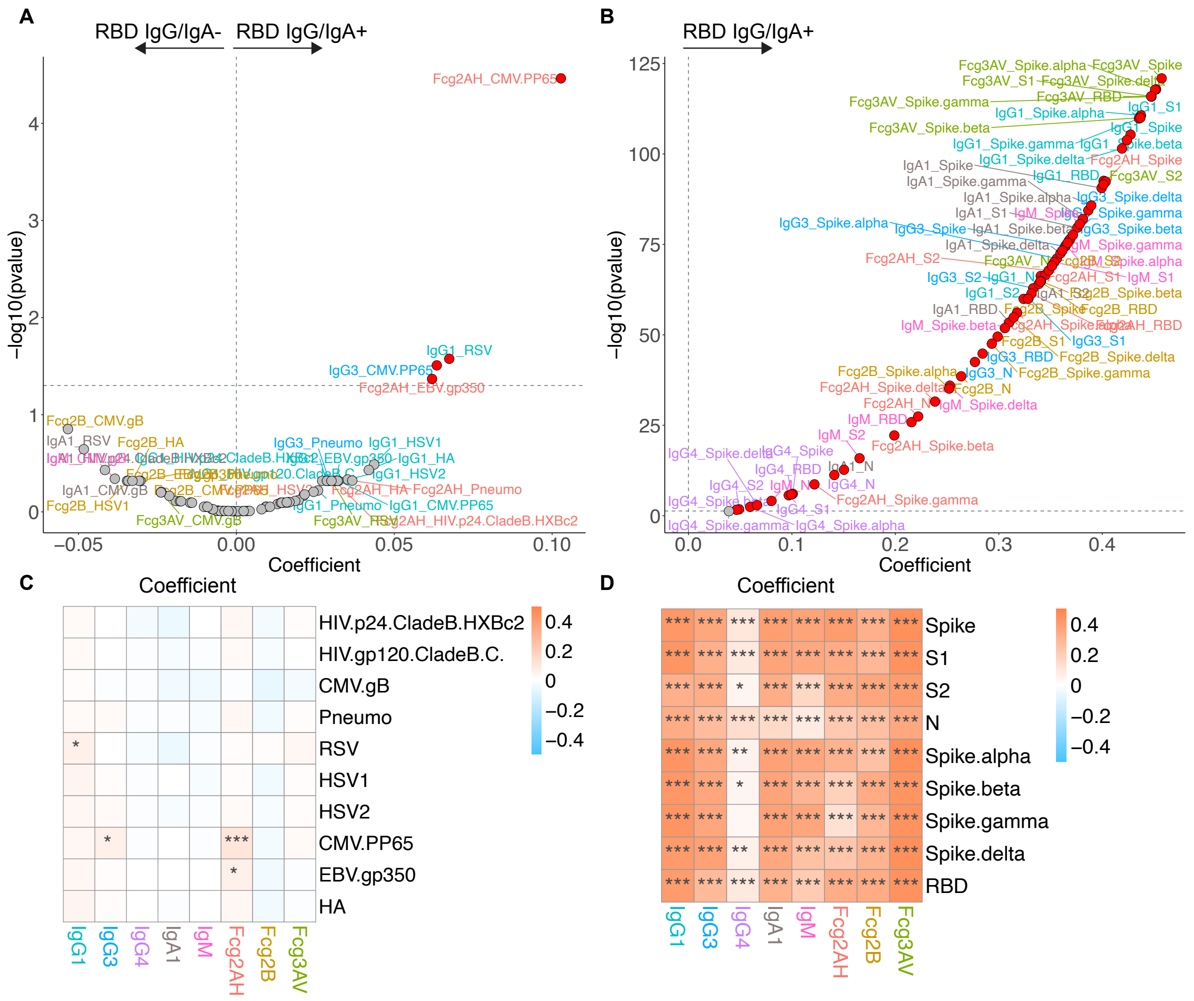

Figure S13. Volcano plots and heat maps of effect of SARS-CoV-2 RBD IgG/IgA/Symptoms positivity on the humoral immune repertoire among all participants. Sensitivity analysis of Figure 3. Participants with COVID symptoms and IgG/IgA- are classified in the same group as participants with RBD IgG/IgA+**.** Volcano plots of effect of SARS-CoV-2 RBD IgG/IgA positivity on the **(A)** non-SARS-CoV-2 humoral repertoire and **(B)** SARS-CoV-2 humoral repertoire among all participants. Volcano plots constructed from linear regression models, adjusted for age, sex, GBD region, nadir CD4, and HIV viral load, with horizontal dashed line of significance displayed for FDR-corrected P=0.05. Responses higher in the antibody-positive fall toward the right of the vertical dashed line, while responses higher in the antibody-negative fall toward the left of the vertical dashed line. Respective heat maps of the volcano plot coefficients for the **(C)** non-SARS-CoV-2 and **(D)** SARS-CoV-2 humoral responses. Coefficients >0 are higher in the antibody-positive, while coefficients <0 are higher in the antibody-negative. Significance in the heat maps is shown as FDR-corrected P<0.05 (*), P<0.01 (**), or P<0.001 (***). Specific antibody isotype, subclass, and Fc-receptor responses are color-coded between the volcano plots and heat maps.

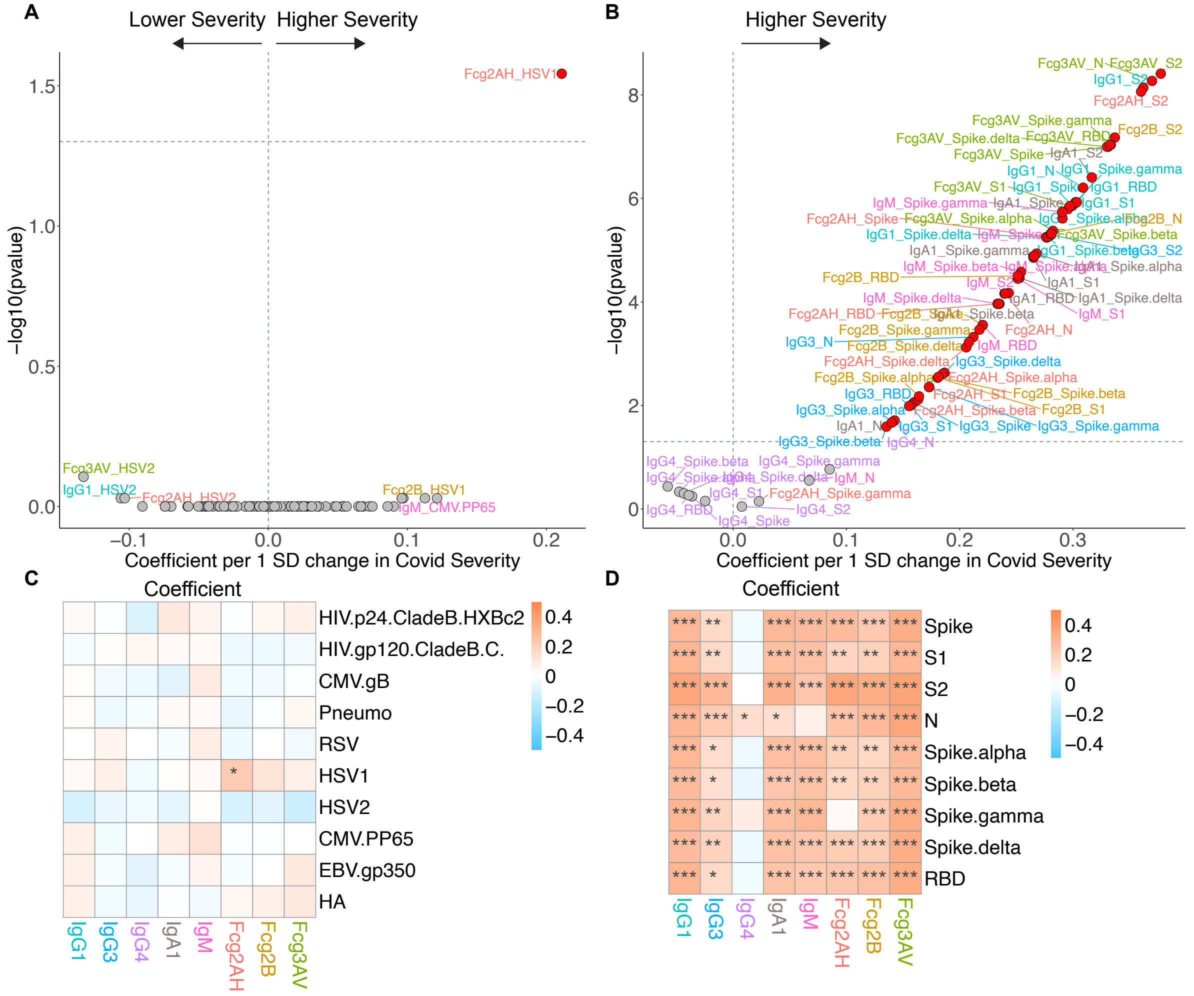

Figure S14. Volcano plots and heat maps of effect of COVID-19 severity on the humoral immune repertoire among COVID-positive participants. Sensitivity analysis of Figure 4. Participants with COVID symptoms and IgG/IgA- are classified in the same group as participants with RBD IgG/IgA+**.** Adjusted volcano plots of effect of COVID-19 severity on the **(A)** non-SARS-CoV-2 humoral repertoire and **(B)** SARS-CoV-2 humoral repertoire among COVID-positive participants. Coefficients reflect the effect of a 1 SD increase in severity, which was z-scored for each participant from the ordinal scale of none reported/asymptomatic, mild, moderate, or severe. Respective heat maps of the volcano plot coefficients for the **(C)** non-SARS-CoV-2 and **(D)** SARS-CoV-2 humoral responses. See Figure 3 legend for details.

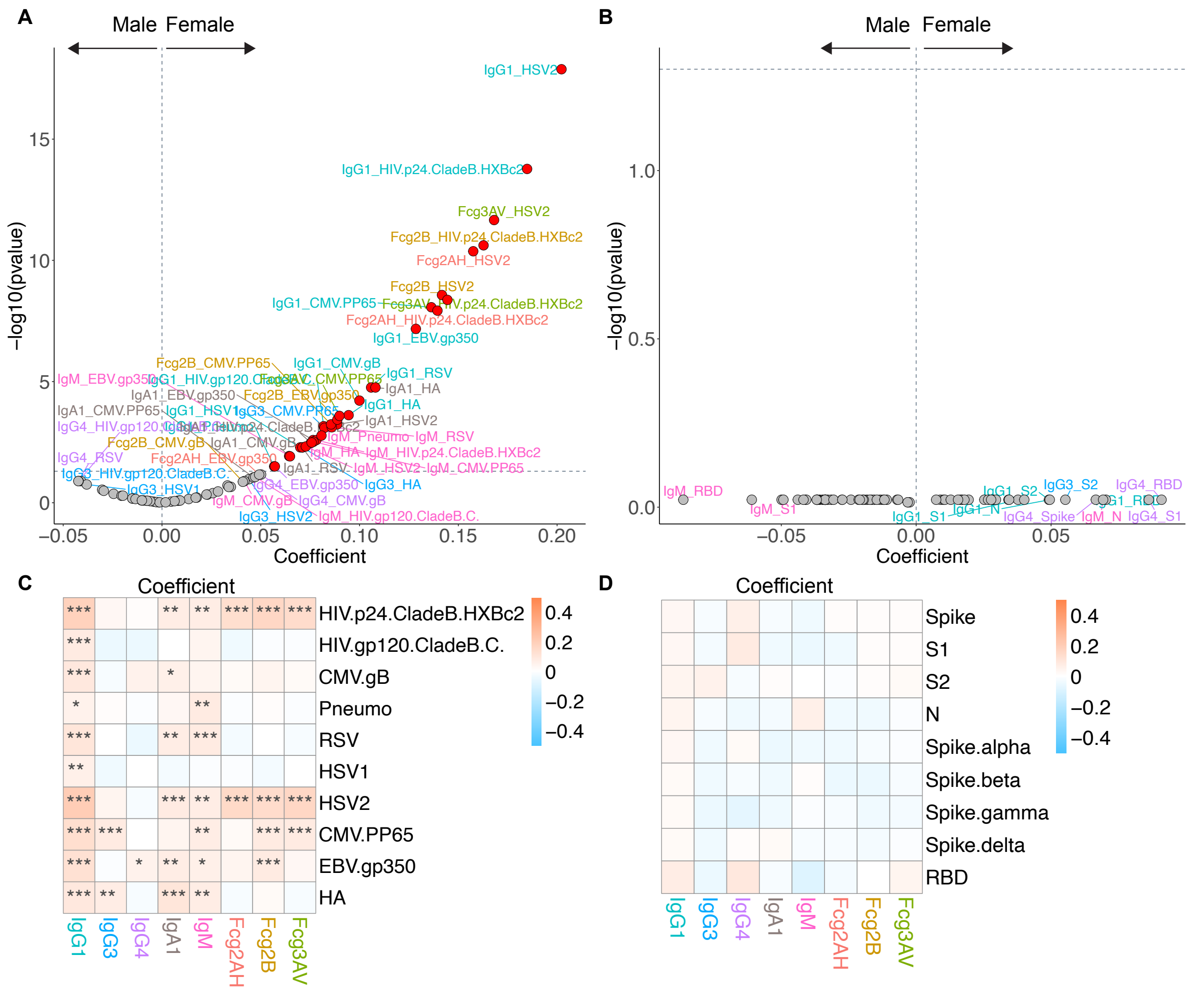

Figure S15. Volcano plots and heat maps of effect of natal sex on the humoral immune repertoire. Sensitivity analysis of Figure 5. Participants with COVID symptoms and IgG/IgA- are classified in the same group as participants with RBD IgG/IgA+**.** Adjusted volcano plots of effect of natal sex on the **(A)** non-SARS-CoV-2 humoral repertoire among the COVID-negative cohort and **(B)** SARS-CoV-2 humoral repertoire among the COVID-positive cohort. Respective heat maps of the volcano plot coefficients for the **(C)** non-SARS-CoV-2 and **(D)** SARS-CoV-2 humoral responses. See Figure 3 legend for details.

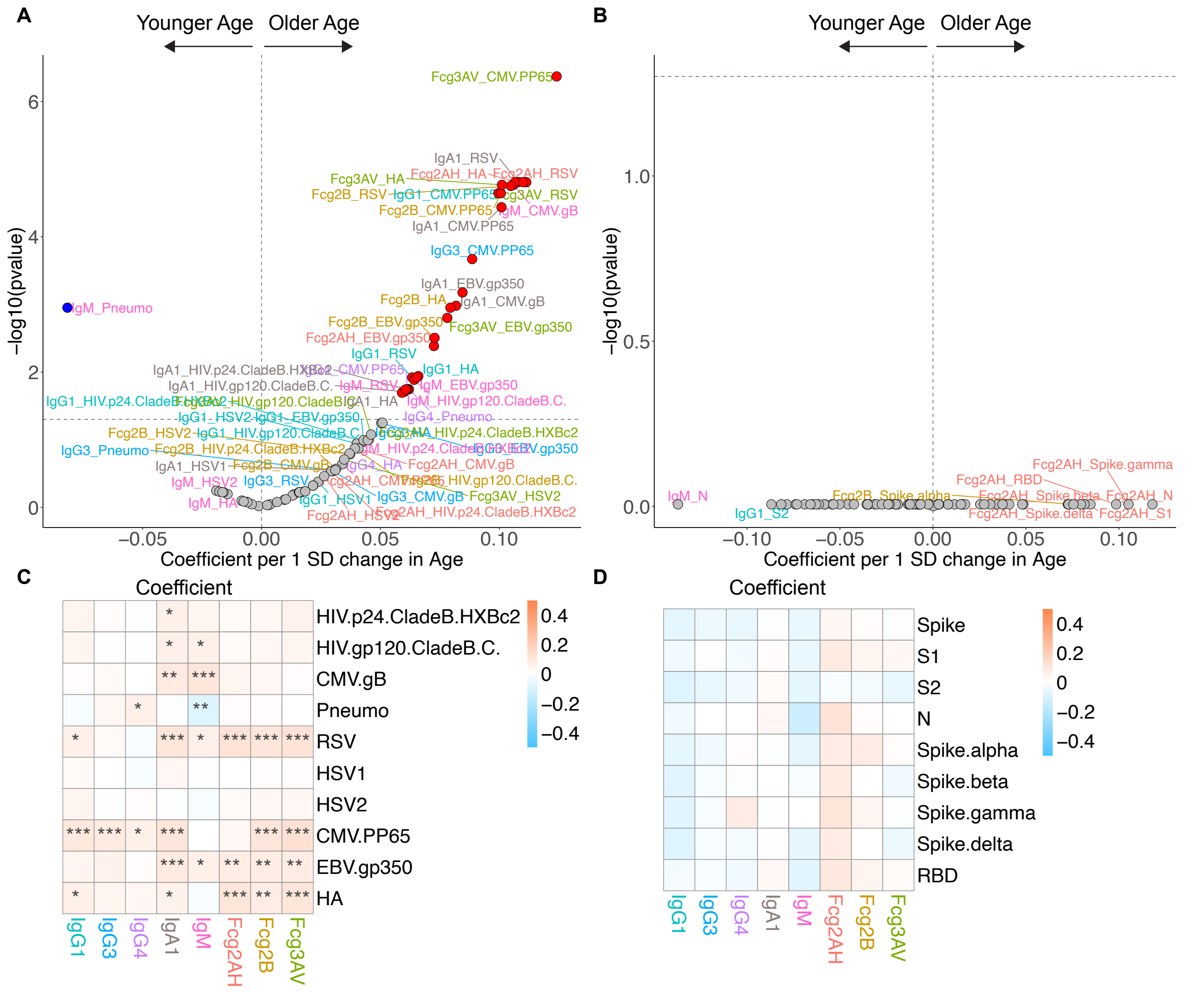

Figure S16. Volcano plots and heat maps of effect of age on the humoral immune repertoire. Sensitivity analysis of Figure 6. Participants with COVID symptoms and IgG/IgA- are classified in the same group as participants with RBD IgG/IgA+**.** Adjusted volcano plots of effect of age on the **(A)** non-SARS-CoV-2 humoral repertoire among the COVID-negative cohort and **(B)** SARS-CoV-2 humoral repertoire among COVID-positive cohort. Coefficients reflect the effect of a 1 SD increase in age, which was z-scored for each participant. Respective heat maps of the volcano plot coefficients for the **(C)** non-SARS-CoV-2 and **(D)** SARS-CoV-2 humoral responses. See Figure 3 legend for details.

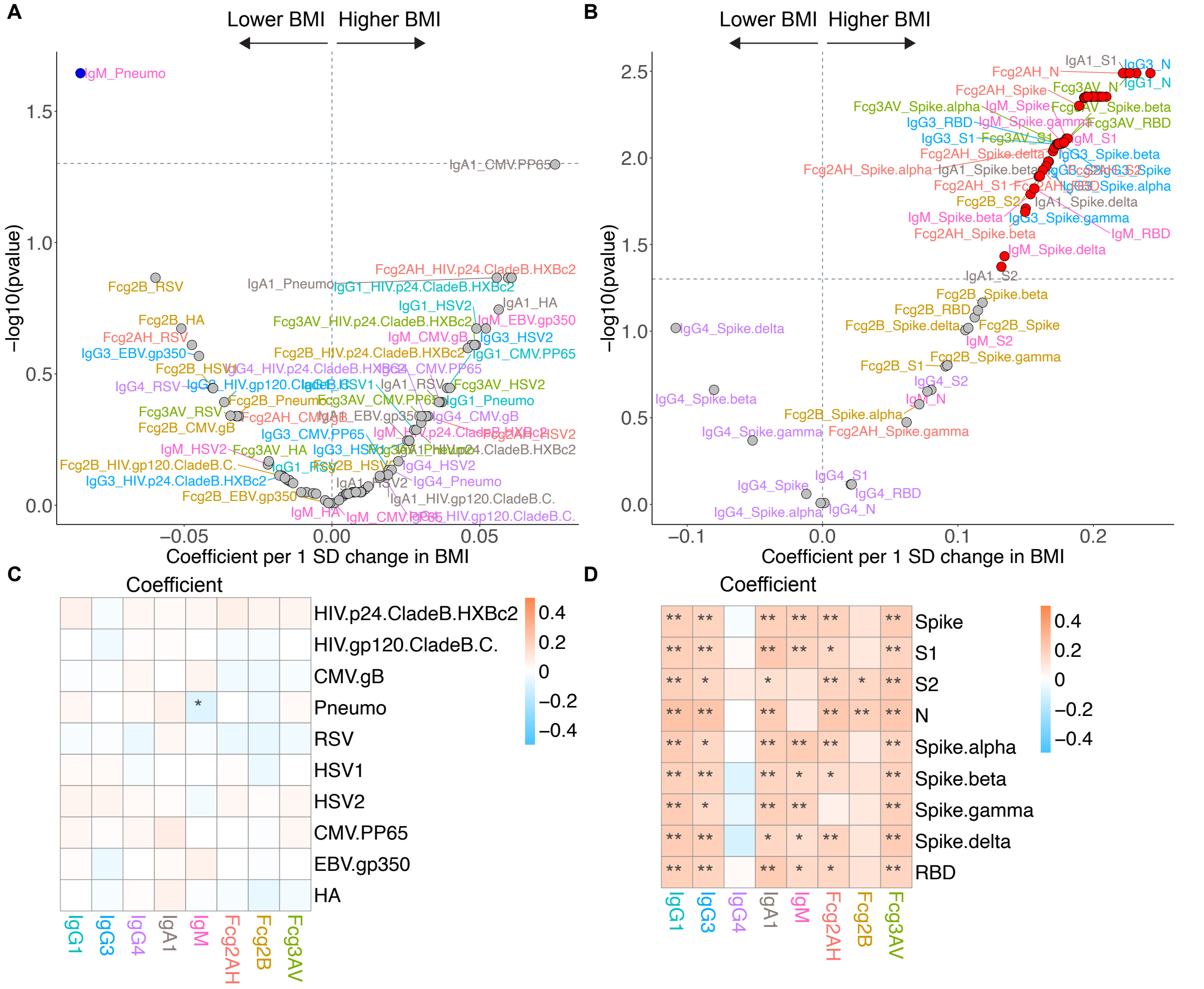

Figure S17. Volcano plots and heat maps of effect of BMI on the humoral immune repertoire. Sensitivity analysis of Figure 7. Participants with COVID symptoms and IgG/IgA- are classified in the same group as participants with RBD IgG/IgA+**.** Adjusted volcano plots of effect of BMI on the **(A)** non-SARS-CoV-2 humoral repertoire among the COVID-negative cohort and **(B)** SARS-CoV-2 humoral repertoire among COVID-positive cohort. Coefficients reflect the effect of a 1 SD increase in BMI, which was z-scored for each participant. Respective heat maps of the volcano plot coefficients for the **(C)** non-SARS-CoV-2 and **(D)** SARS-CoV-2 humoral responses. See Figure 3 legend for details.

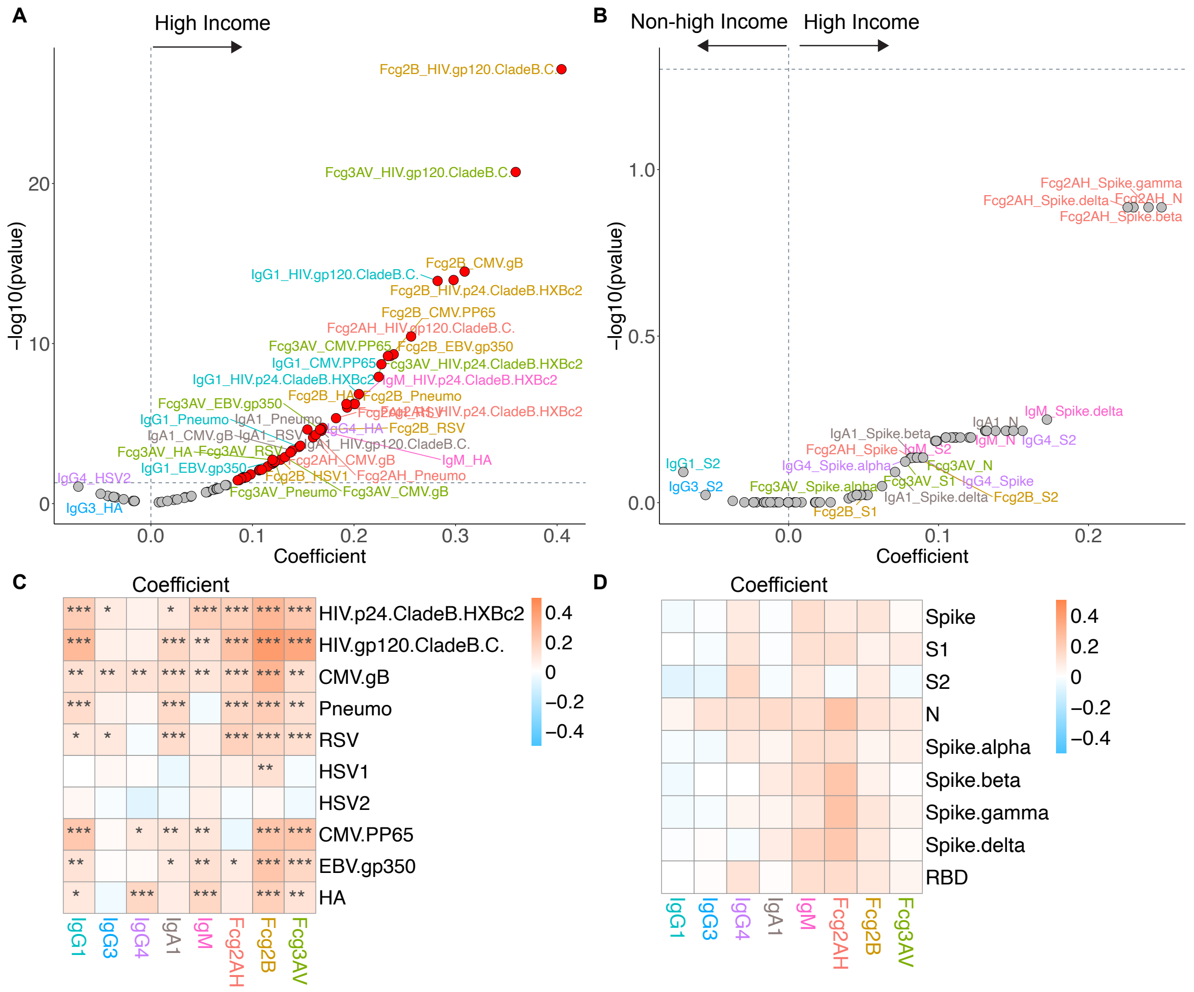

Figure S18. Volcano plots and heat maps of effect of GBD region on the humoral immune repertoire. Sensitivity analysis of Figure 8. Participants with COVID symptoms and IgG/IgA- are classified in the same group as participants with RBD IgG/IgA+**.** Adjusted volcano plots of effect of high-income GBD region (vs non-high-income) on the **(A)** non-SARS-CoV-2 humoral repertoire among the COVID-negative cohort and **(B)** SARS-CoV-2 humoral repertoire among the COVID-positive cohort. Respective heat maps of the volcano plot coefficients for the **(C)** non-SARS-CoV-2 and **(D)** SARS-CoV-2 humoral responses. See Figure 3 legend for details.

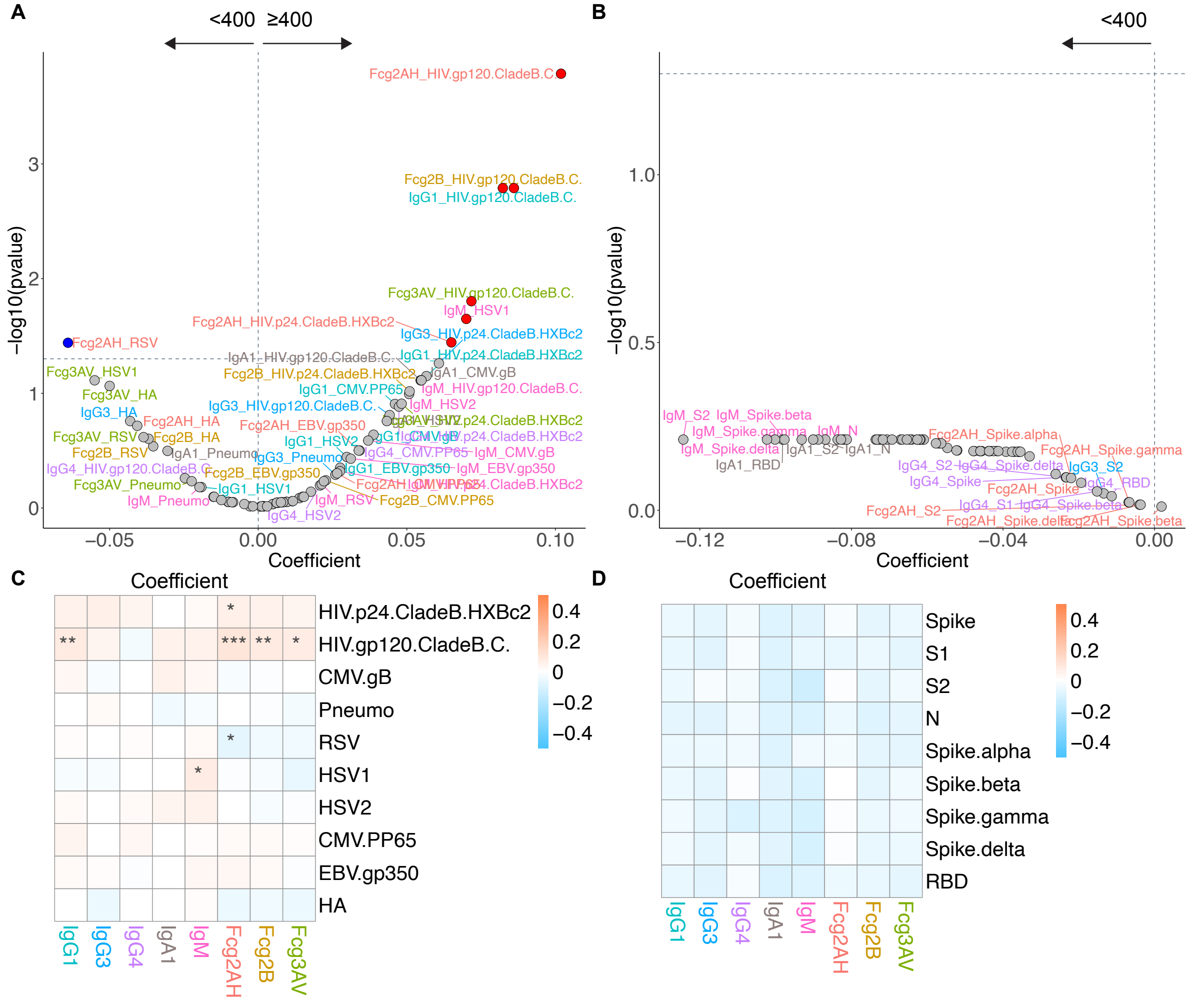

Figure S19. Volcano plots and heat maps of effect of HIV viremia on the humoral immune repertoire. Sensitivity analysis of Figure 9. Participants with COVID symptoms and IgG/IgA- are classified in the same group as participants with RBD IgG/IgA+**.** Adjusted volcano plots of effect of HIV viremia (≥400 vs <400 copies/ml) on the **(A)** non-SARS-CoV-2 humoral repertoire among the COVID-negative cohort and **(B)** SARS-CoV-2 humoral repertoire among the COVID-positive cohort. Respective heat maps of the volcano plot coefficients for the **(C)** non-SARS-CoV-2 and **(D)** SARS-CoV-2 humoral responses. See Figure 3 legend for details.

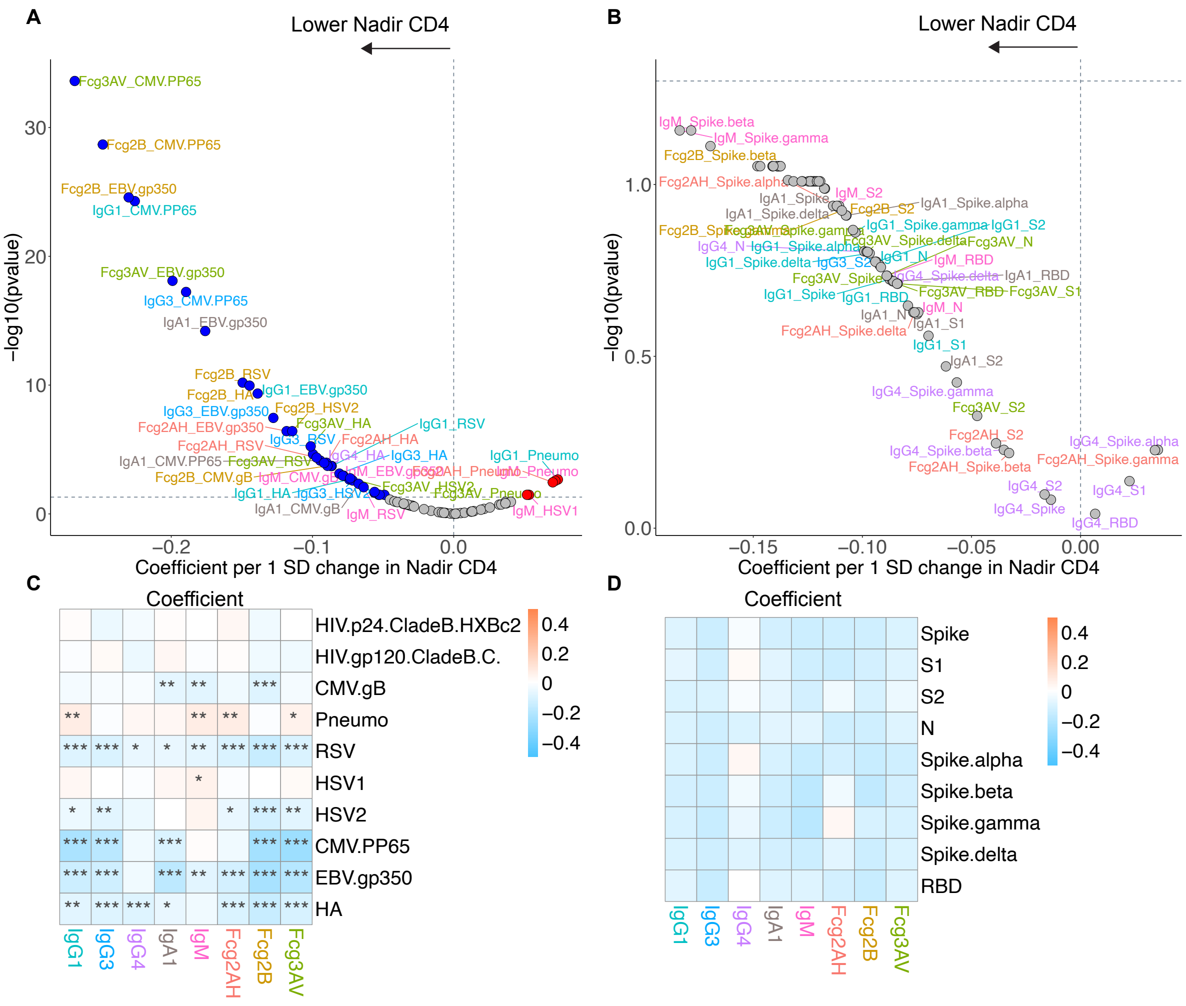

Figure S20. Volcano plots and heat maps of effect of nadir CD4 on the humoral immune repertoire. Sensitivity analysis of Figure 10. Participants with COVID symptoms and IgG/IgA- are classified in the same group as participants with RBD IgG/IgA+**.** Adjusted volcano plots of effect of nadir CD4 on the **(A)** non-SARS-CoV-2 humoral repertoire among the COVID-negative cohort and **(B)** SARS-CoV-2 humoral repertoire among the COVID-positive cohort. Coefficients reflect the effect of a 1 SD increase in nadir CD4, which was z-scored for each participant from the ordinal scale of <50, 50-199, 200-349, or ≥350 cell/mm^3. Respective heat maps of the volcano plot coefficients for the **(C)** non-SARS-CoV-2 and **(D)** SARS-CoV-2 humoral responses. See Figure 3 legend for details.

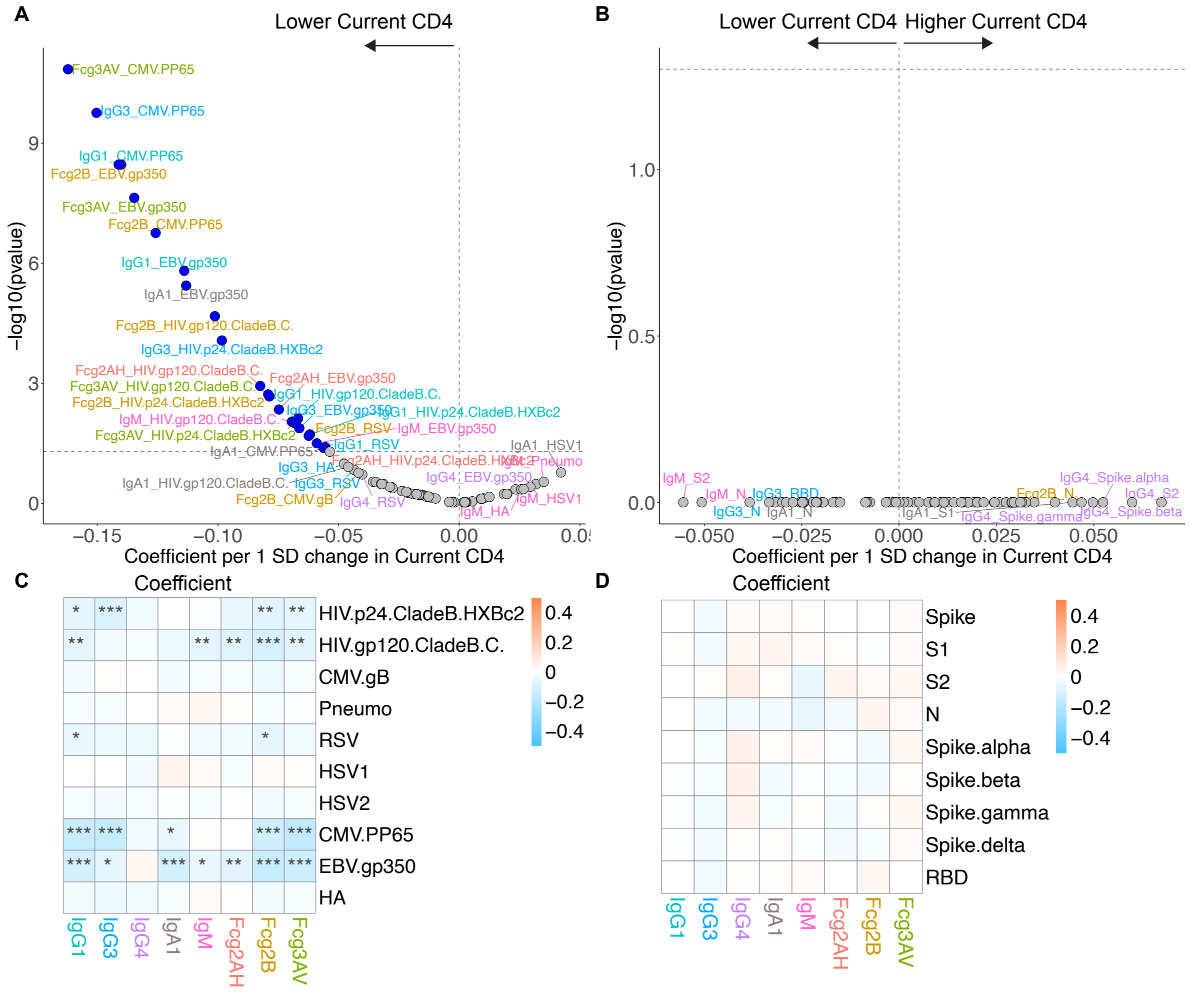

Figure S21. Volcano plots and heat maps of effect of current CD4 on the humoral immune repertoire. Sensitivity analysis of Figure 11. Participants with COVID symptoms and IgG/IgA- are classified in the same group as participants with RBD IgG/IgA+. Adjusted volcano plots of effect of current CD4 (without nadir CD4 adjustment) on the **(A)** non-SARS-CoV-2 humoral repertoire among the COVID-negative cohort and **(B)** SARS-CoV-2 humoral repertoire among COVID-positive cohort. Coefficients reflect the effect of a 1 SD increase in current CD4 (cells/mm^3), which was z-scored for each participant. Respective heat maps of the volcano plot coefficients for the **(C)** non-SARS-CoV-2 and **(D)** SARS-CoV-2 humoral responses. See Figure 3 legend for details.

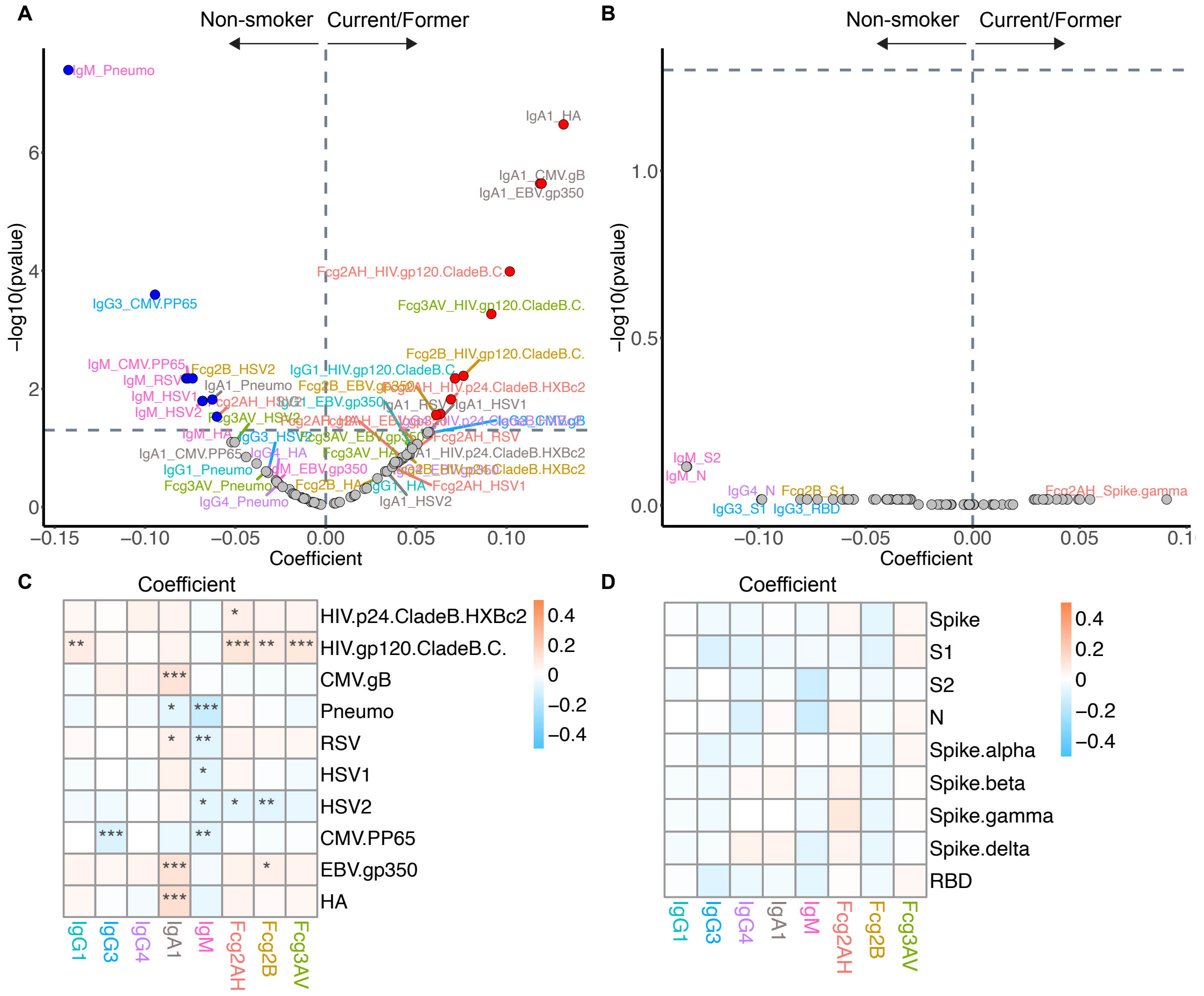

Figure S22. Volcano plots and heat maps of effect of smoking on the humoral immune repertoire. Sensitivity analysis of Figure S10. Participants with COVID symptoms and IgG/IgA- are classified in the same group as participants with RBD IgG/IgA+. Volcano plot of effect of smoking on the **(A)** non-SARS-CoV-2 humoral repertoire among the COVID-negative cohort and **(B)** SARS-CoV-2 humoral repertoire among the COVID-positive cohort. Volcano plots constructed from linear regression models, adjusted for age, sex, GBD region, nadir CD4, and HIV viral load, with horizontal dashed line of significance displayed for FDR-corrected P=0.05. Responses higher in current/former smoking fall toward the right of the vertical dashed line, while responses higher in never-smoking fall toward the left of the vertical dashed line. Respective heat maps of the volcano plot coefficients for the **(C)** non-SARS-CoV-2 and **(D)** SARS-CoV-2 humoral responses. Coefficients >0 are higher in current/former smoking, while coefficients <0 are higher in never-smoking. Significance in the heat maps is shown as FDR-corrected P<0.05 (*), P<0.01 (**), or P<0.001 (***). Specific antibody isotype, subclass, and Fc-receptor responses are color-coded between the volcano plots and heat maps.

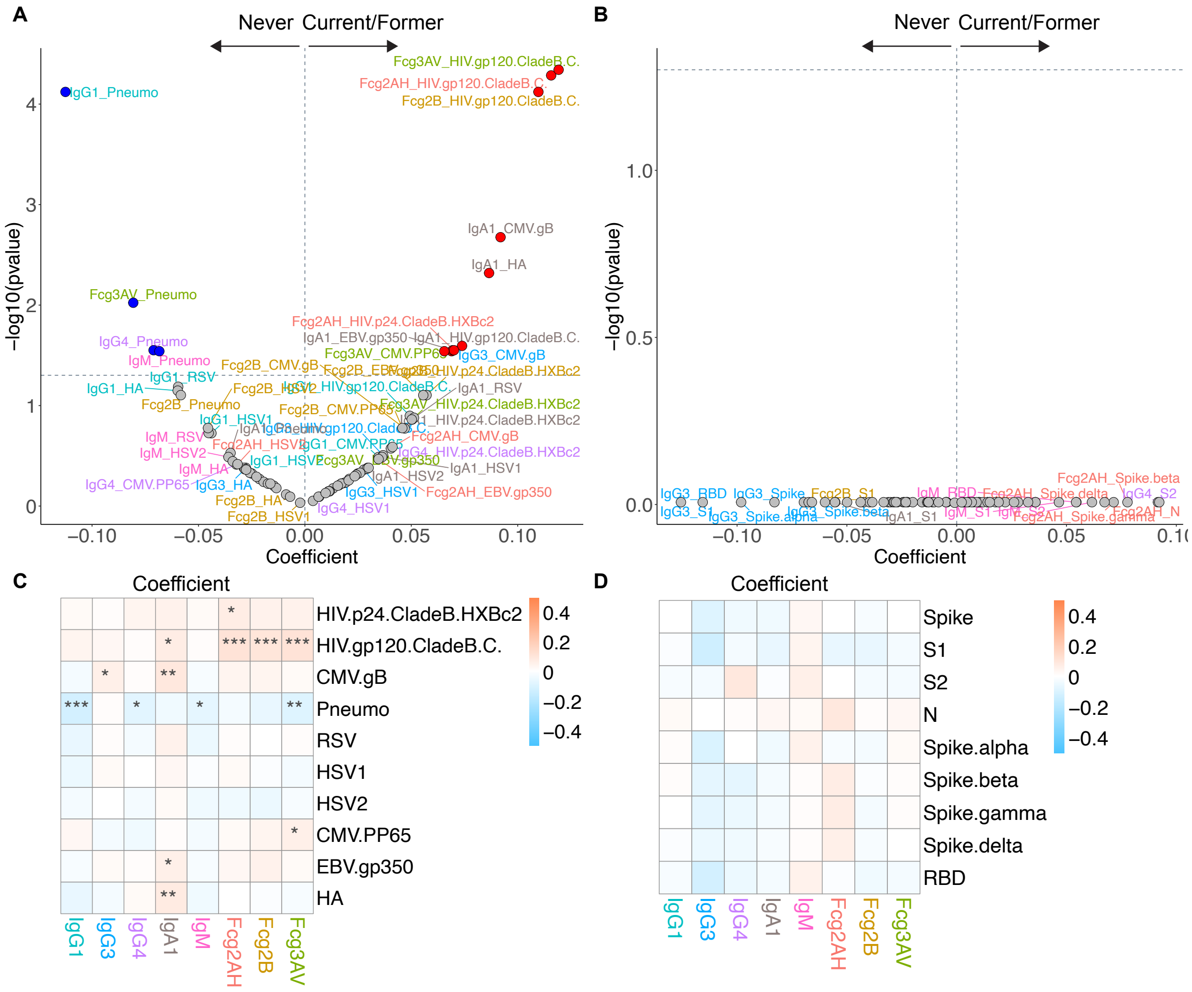

Figure S23. Volcano plots and heat maps of effect of substance use on the humoral immune repertoire. Sensitivity analysis of Figure S11. Participants with COVID symptoms and IgG/IgA- are classified in the same group as participants with RBD IgG/IgA+**.** Volcano plot of effect of substance use on the **(A)** non-SARS-CoV-2 humoral repertoire among the COVID-negative cohort and **(B)** SARS-CoV-2 humoral repertoire among the COVID-positive cohort. Volcano plots constructed from linear regression models, adjusted for age, sex, GBD region, nadir CD4, and HIV viral load, with horizontal dashed line of significance displayed for FDR-corrected P=0.05. Responses higher in current/former substance use fall toward the right of the vertical dashed line, while responses higher in never-substance use fall toward the left of the vertical dashed line. Respective heat maps of the volcano plot coefficients for the **(C)** non-SARS-CoV-2 and **(D)** SARS-CoV-2 humoral responses. Coefficients >0 are higher in current/former substance use, while coefficients <0 are higher in never-substance use. Significance in the heat maps is shown as FDR-corrected P<0.05 (*), P<0.01 (**), or P<0.001 (***). Specific antibody isotype, subclass, and Fc-receptor responses are color-coded between the volcano plots and heat maps.

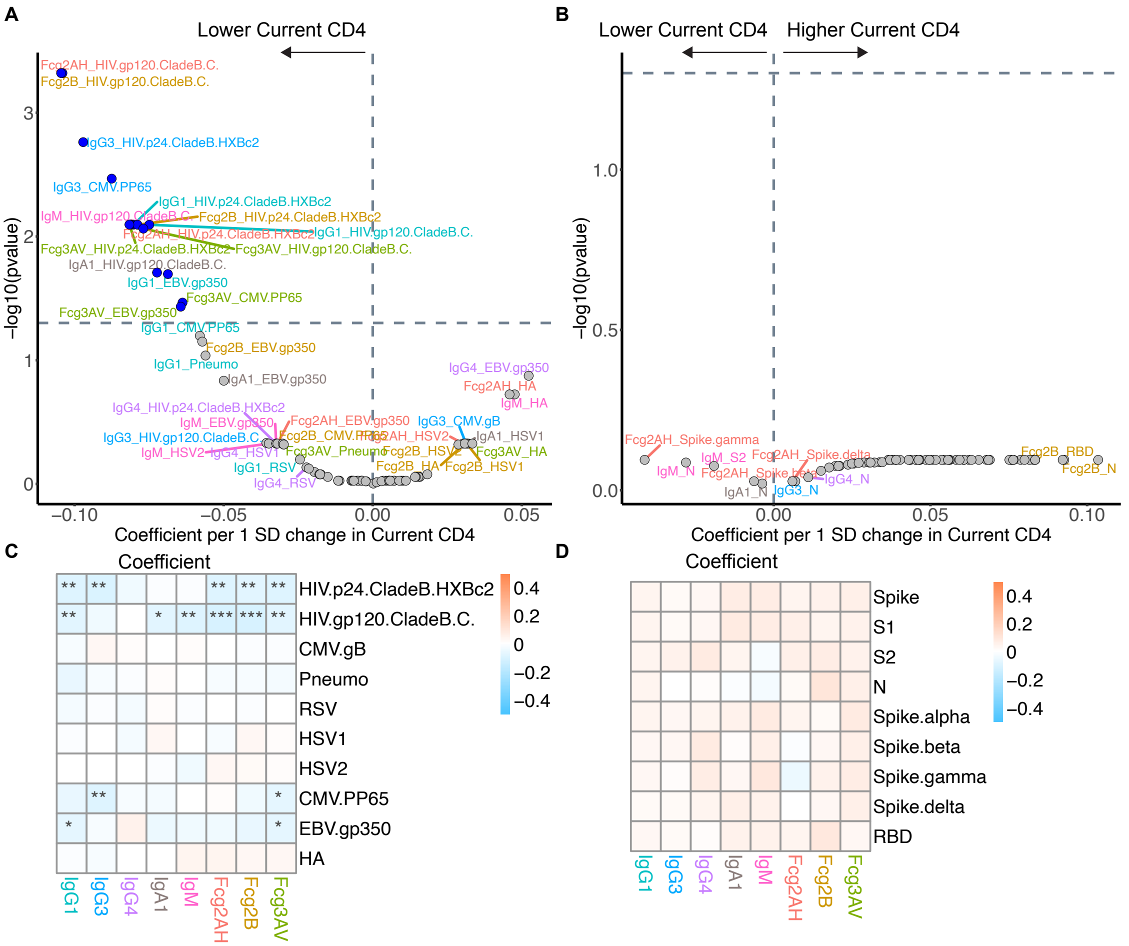

Figure S24. Volcano plots and heat maps of effect of current CD4 adjusted for nadir CD4 on the humoral immune repertoire. Sensitivity analysis of Figure S12. Participants with COVID symptoms and IgG/IgA- are classified in the same group as participants with RBD IgG/IgA+**.** Volcano plot of effect of current CD4 adjusted for nadir CD4 on the **(A)** non-SARS-CoV-2 humoral repertoire among the COVID-negative cohort and **(B)** SARS-CoV-2 humoral repertoire among COVID-positive cohort. Volcano plots constructed from linear regression models, adjusted for age, sex, GBD region, nadir CD4, and HIV viral load, with horizontal dashed line of significance displayed for FDR-corrected P=0.05. Coefficients reflect the effect of a 1 SD increase in current CD4 (cells/mm^3), which was z-scored for each participant. Responses higher with higher current CD4 fall toward the right of the vertical dashed line, while responses higher with lower current CD4 fall toward the left of the vertical dashed line. Respective heat maps of the volcano plot coefficients for the **(C)** non-SARS-CoV-2 and **(D)** SARS-CoV-2 humoral responses. Coefficients >0 are higher with higher current CD4, while coefficients <0 are higher with lower current CD4. Significance in the heat maps is shown as FDR-corrected P<0.05 (*), P<0.01 (**), or P<0.001 (***). Specific antibody isotype, subclass, and Fc-receptor responses are color-coded between the volcano plots and heat maps.

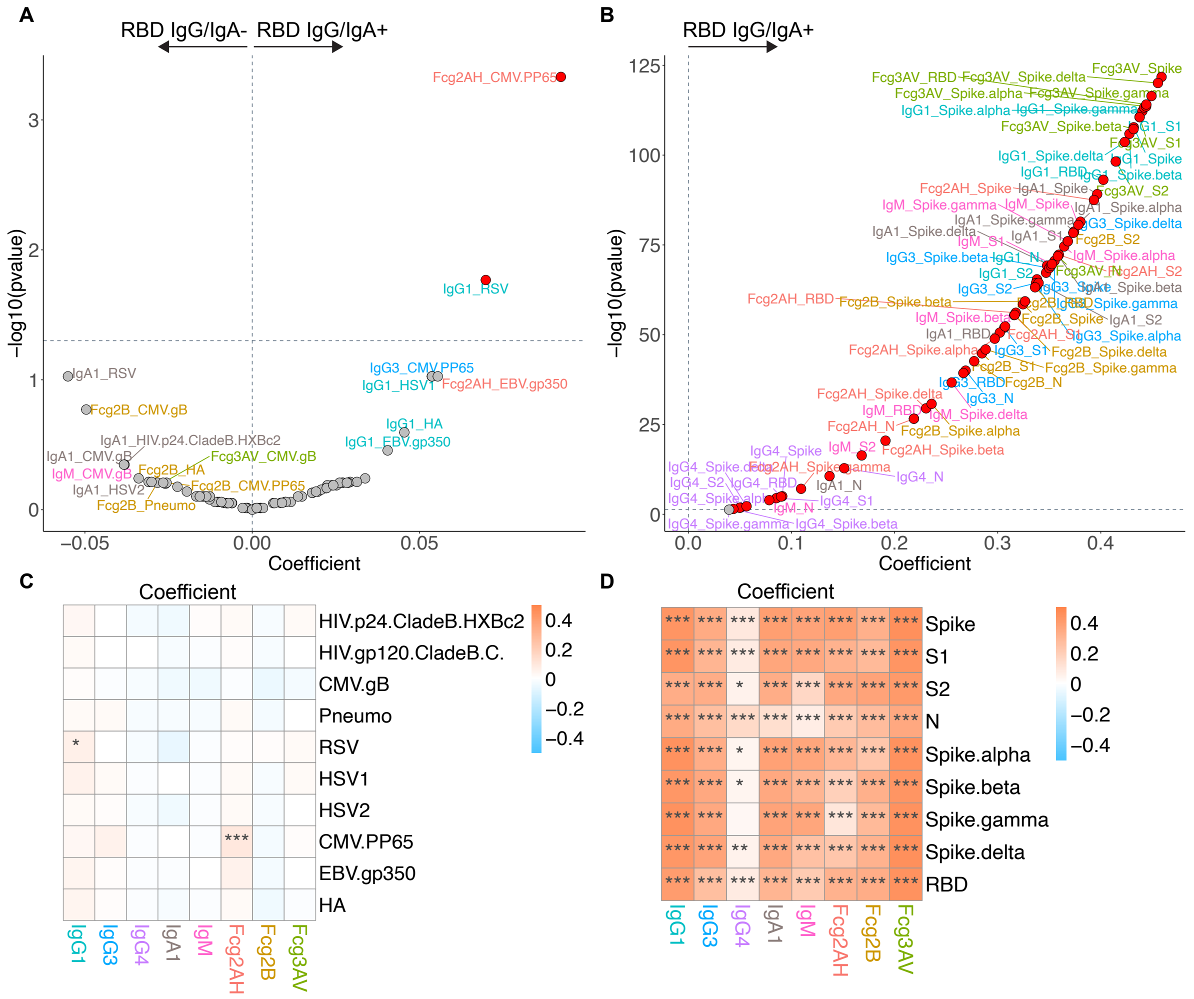

Figure S25. Volcano plots and heat maps of effect of SARS-CoV-2 RBD IgG/IgA positivity on the humoral immune repertoire among all participants. Sensitivity analysis of Figure 3. Participants with COVID symptoms and IgG/IgA- are removed from the group with RBD IgG/IgA**-**. Volcano plots of effect of SARS-CoV-2 RBD IgG/IgA positivity on the **(A)** non-SARS-CoV-2 humoral repertoire and **(B)** SARS-CoV-2 humoral repertoire among all participants. Volcano plots constructed from linear regression models, adjusted for age, sex, GBD region, nadir CD4, and HIV viral load, with horizontal dashed line of significance displayed for FDR-corrected P=0.05. Responses higher in the antibody-positive fall toward the right of the vertical dashed line, while responses higher in the antibody-negative fall toward the left of the vertical dashed line. Respective heat maps of the volcano plot coefficients for the **(C)** non-SARS-CoV-2 and **(D)** SARS-CoV-2 humoral responses. Coefficients >0 are higher in the antibody-positive, while coefficients <0 are higher in the antibody-negative. Significance in the heat maps is shown as FDR-corrected P<0.05 (*), P<0.01 (**), or P<0.001 (***). Specific antibody isotype, subclass, and Fc-receptor responses are color-coded between the volcano plots and heat maps.

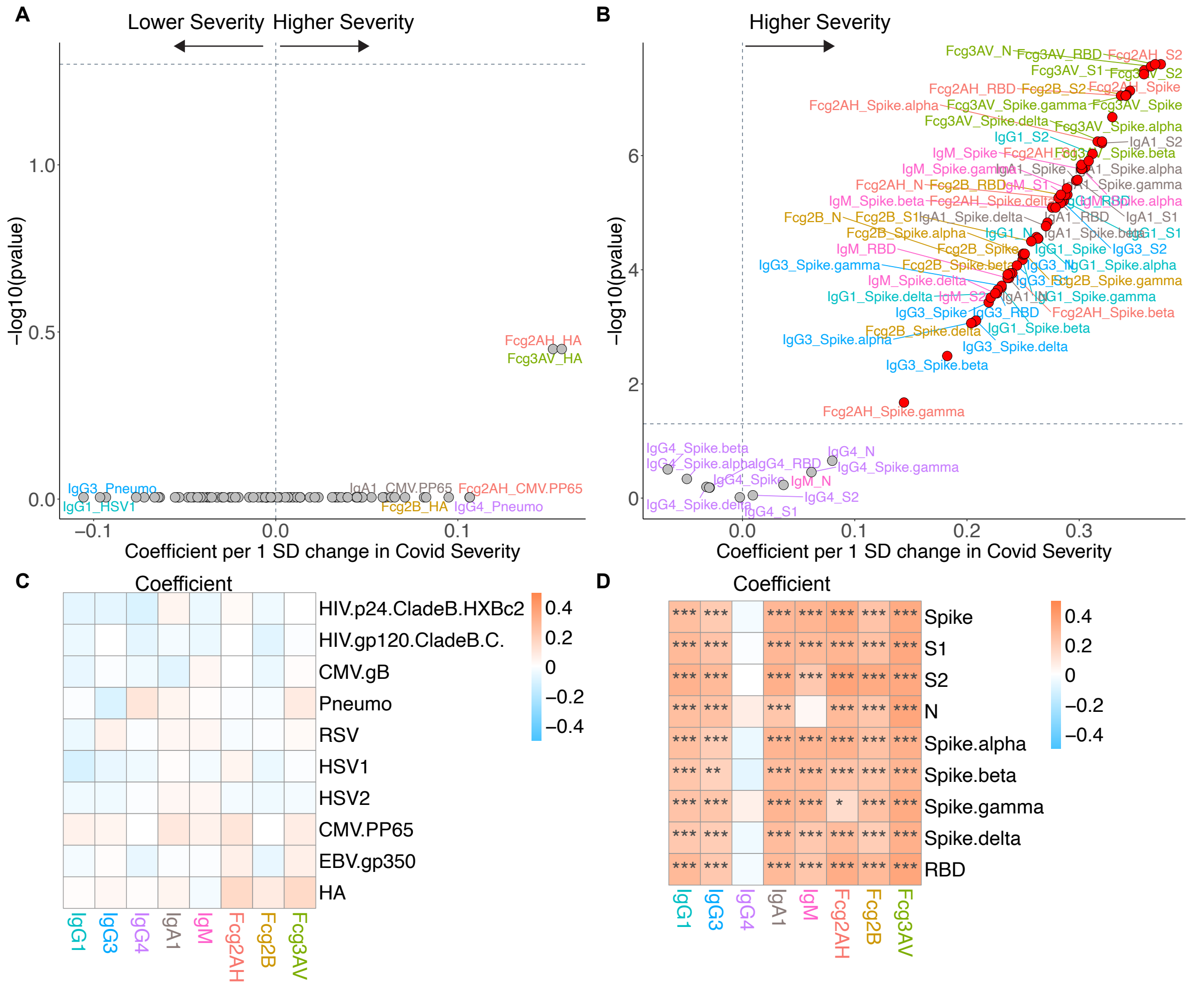

Figure S26. Volcano plots and heat maps of effect of COVID-19 severity on the humoral immune repertoire among COVID-positive participants. Sensitivity analysis of Figure 4. Participants with COVID symptoms and IgG/IgA- are removed from the group with RBD IgG/IgA-**.** Adjusted volcano plots of effect of COVID-19 severity on the **(A)** non-SARS-CoV-2 humoral repertoire and **(B)** SARS-CoV-2 humoral repertoire among COVID-positive participants. Coefficients reflect the effect of a 1 SD increase in severity, which was z-scored for each participant from the ordinal scale of none reported/asymptomatic, mild, moderate, or severe. Respective heat maps of the volcano plot coefficients for the **(C)** non-SARS-CoV-2 and **(D)** SARS-CoV-2 humoral responses. See Figure 3 legend for details.

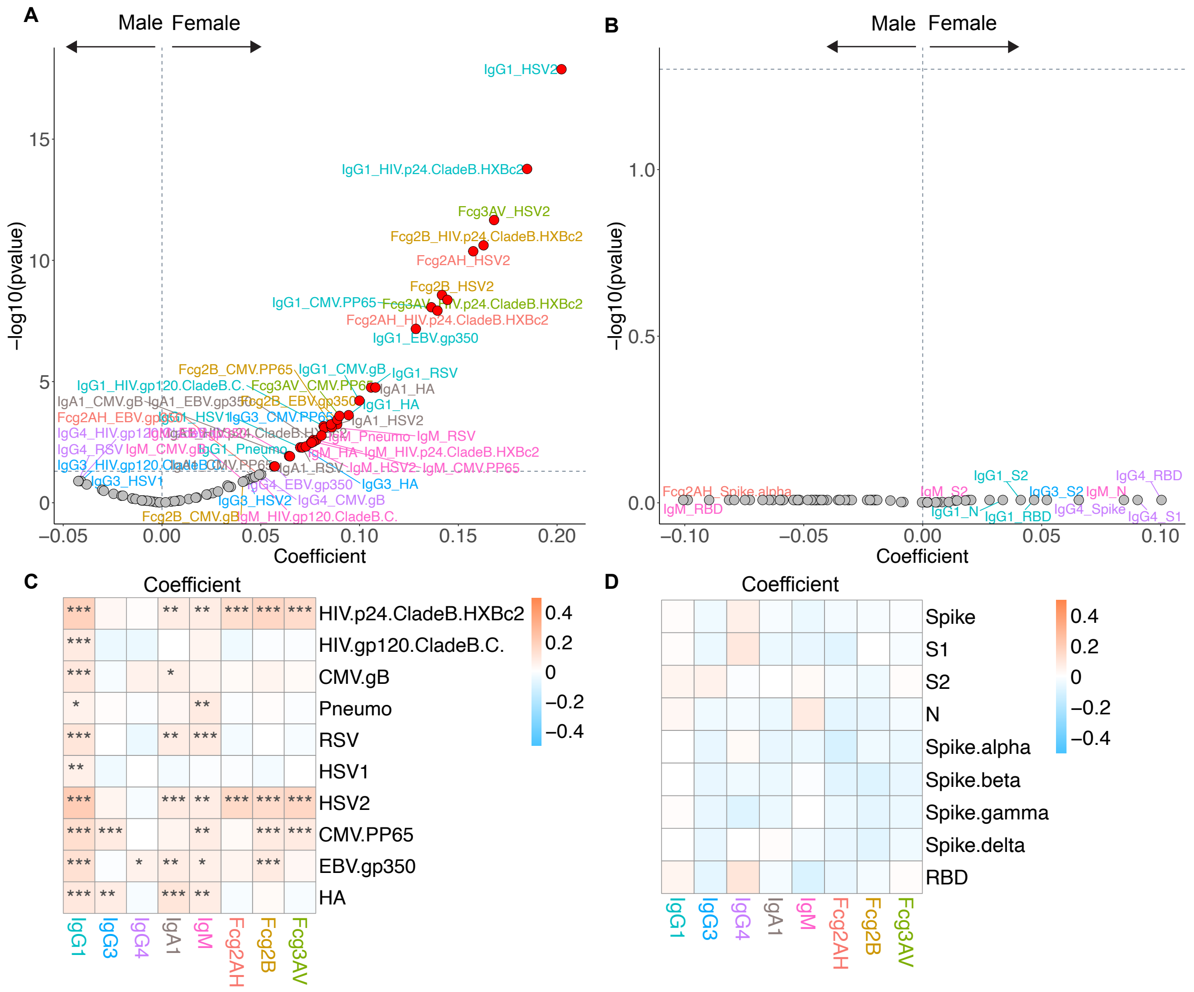

Figure S27. Volcano plots and heat maps of effect of natal sex on the humoral immune repertoire. Sensitivity analysis of Figure 5. Participants with COVID symptoms and IgG/IgA- are removed from the group with RBD IgG/IgA-. Adjusted volcano plots of effect of natal sex on the **(A)** non-SARS-CoV-2 humoral repertoire among the COVID-negative cohort and **(B)** SARS-CoV-2 humoral repertoire among the COVID-positive cohort. Respective heat maps of the volcano plot coefficients for the **(C)** non-SARS-CoV-2 and **(D)** SARS-CoV-2 humoral responses. See Figure 3 legend for details.

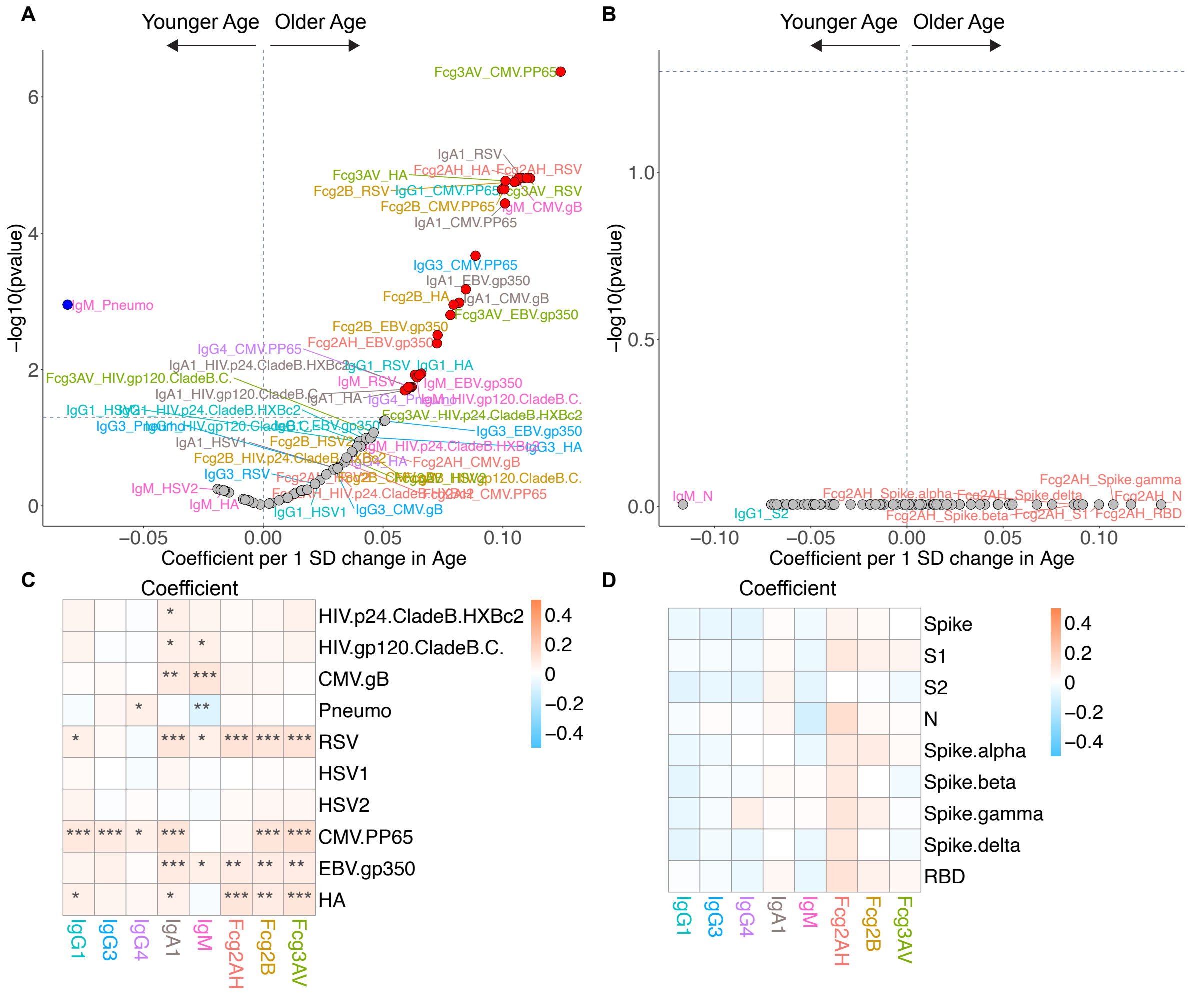

Figure S28. Volcano plots and heat maps of effect of age on the humoral immune repertoire. Sensitivity analysis of Figure 6. Participants with COVID symptoms and IgG/IgA- are removed from the group with RBD IgG/IgA-**.** Adjusted volcano plots of effect of age on the **(A)** non-SARS-CoV-2 humoral repertoire among the COVID-negative cohort and **(B)** SARS-CoV-2 humoral repertoire among COVID-positive cohort. Coefficients reflect the effect of a 1 SD increase in age, which was z-scored for each participant. Respective heat maps of the volcano plot coefficients for the **(C)** non-SARS-CoV-2 and **(D)** SARS-CoV-2 humoral responses. See Figure 3 legend for details.

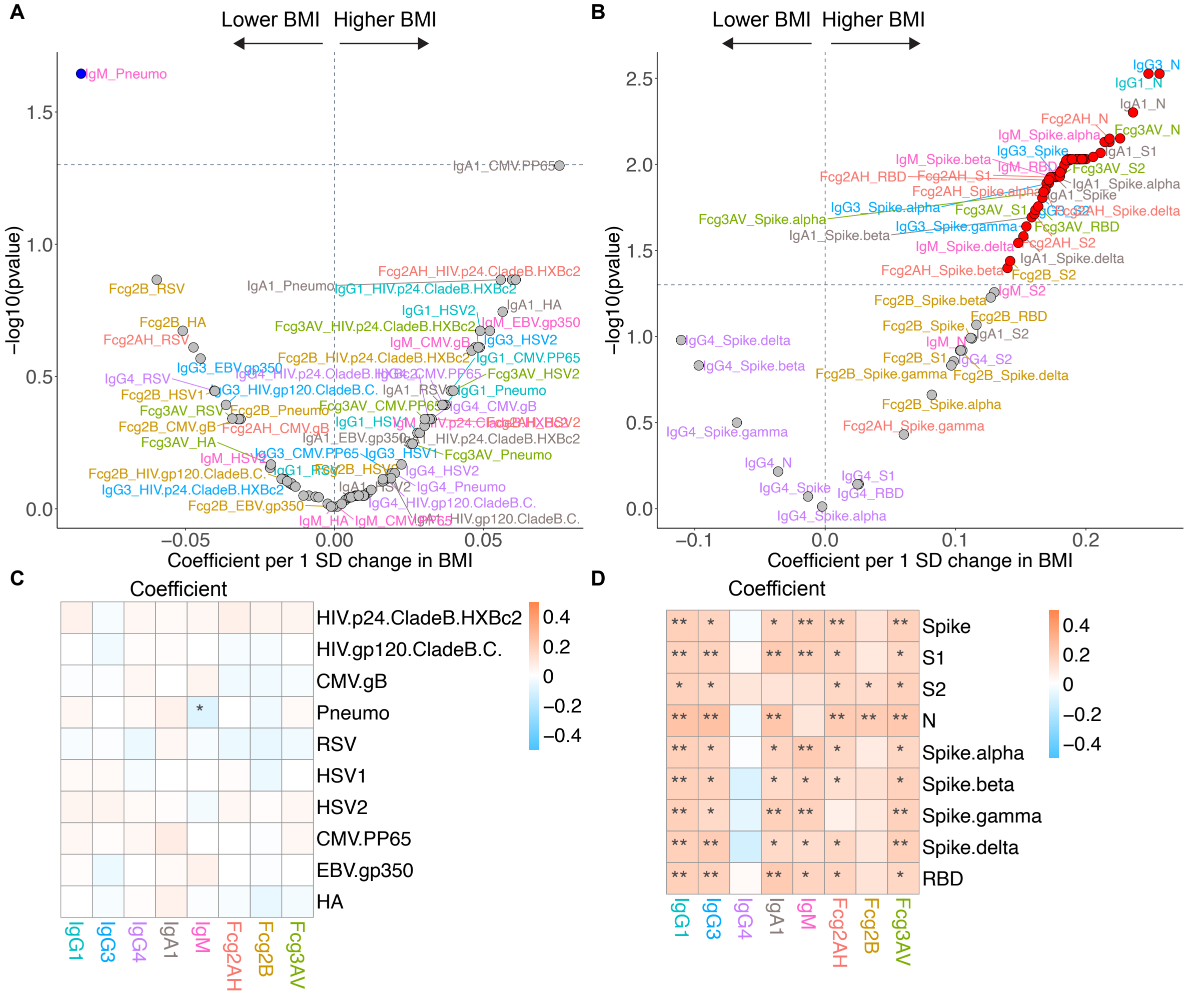

Figure S29. Volcano plots and heat maps of effect of BMI on the humoral immune repertoire. Sensitivity analysis of Figure 7. Participants with COVID symptoms and IgG/IgA- are removed from the group with RBD IgG/IgA-**.** Adjusted volcano plots of effect of BMI on the **(A)** non-SARS-CoV-2 humoral repertoire among the COVID-negative cohort and **(B)** SARS-CoV-2 humoral repertoire among COVID-positive cohort. Coefficients reflect the effect of a 1 SD increase in BMI, which was z-scored for each participant. Respective heat maps of the volcano plot coefficients for the **(C)** non-SARS-CoV-2 and **(D)** SARS-CoV-2 humoral responses. See Figure 3 legend for details.

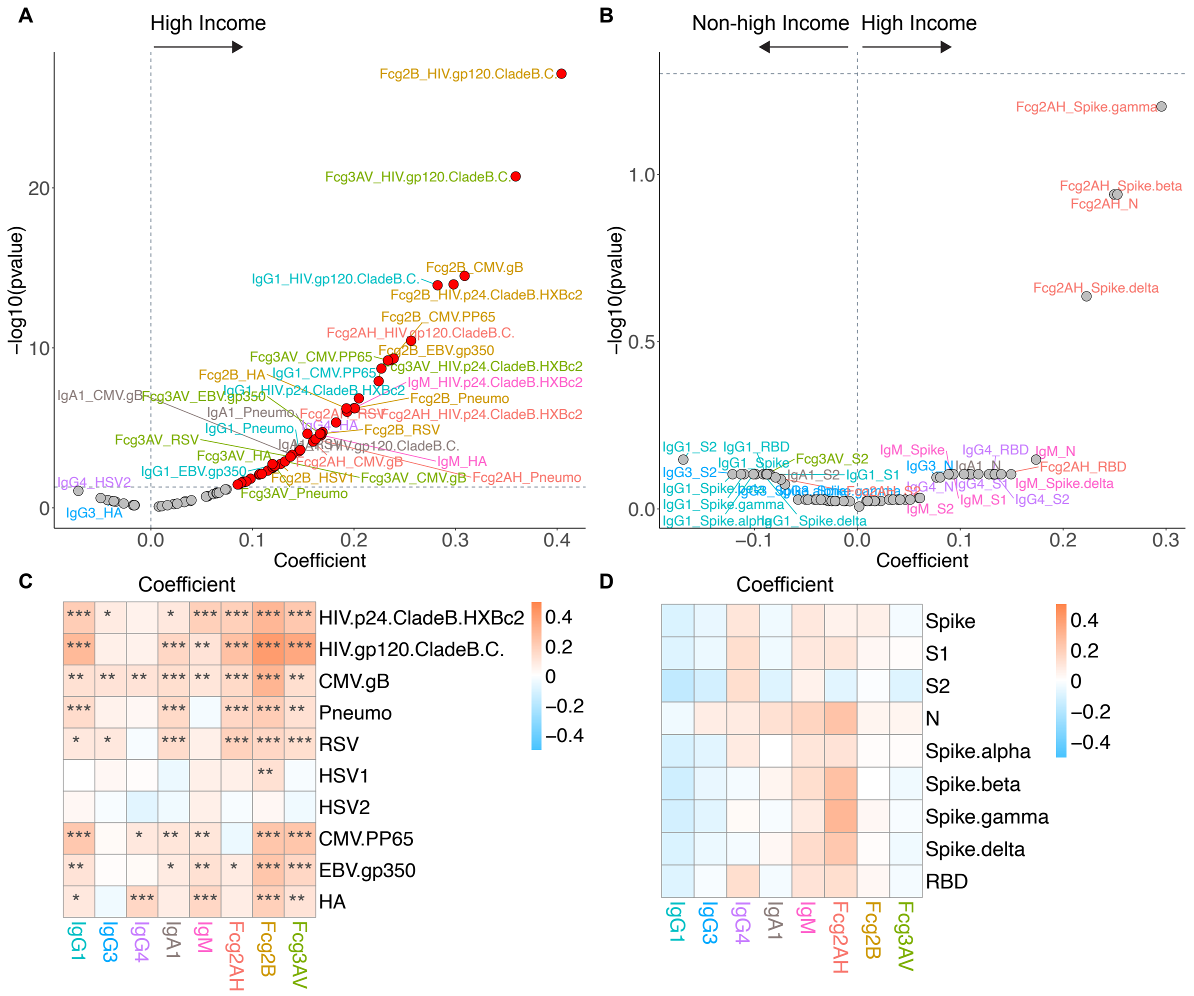

Figure S30. Volcano plots and heat maps of effect of GBD region on the humoral immune repertoire. Sensitivity analysis of Figure 8. Participants with COVID symptoms and IgG/IgA- are removed from the group with RBD IgG/IgA-. Adjusted volcano plots of effect of high-income GBD region (vs non-high-income) on the **(A)** non-SARS-CoV-2 humoral repertoire among the COVID-negative cohort and **(B)** SARS-CoV-2 humoral repertoire among the COVID-positive cohort. Respective heat maps of the volcano plot coefficients for the **(C)** non-SARS-CoV-2 and **(D)** SARS-CoV-2 humoral responses. See Figure 3 legend for details.

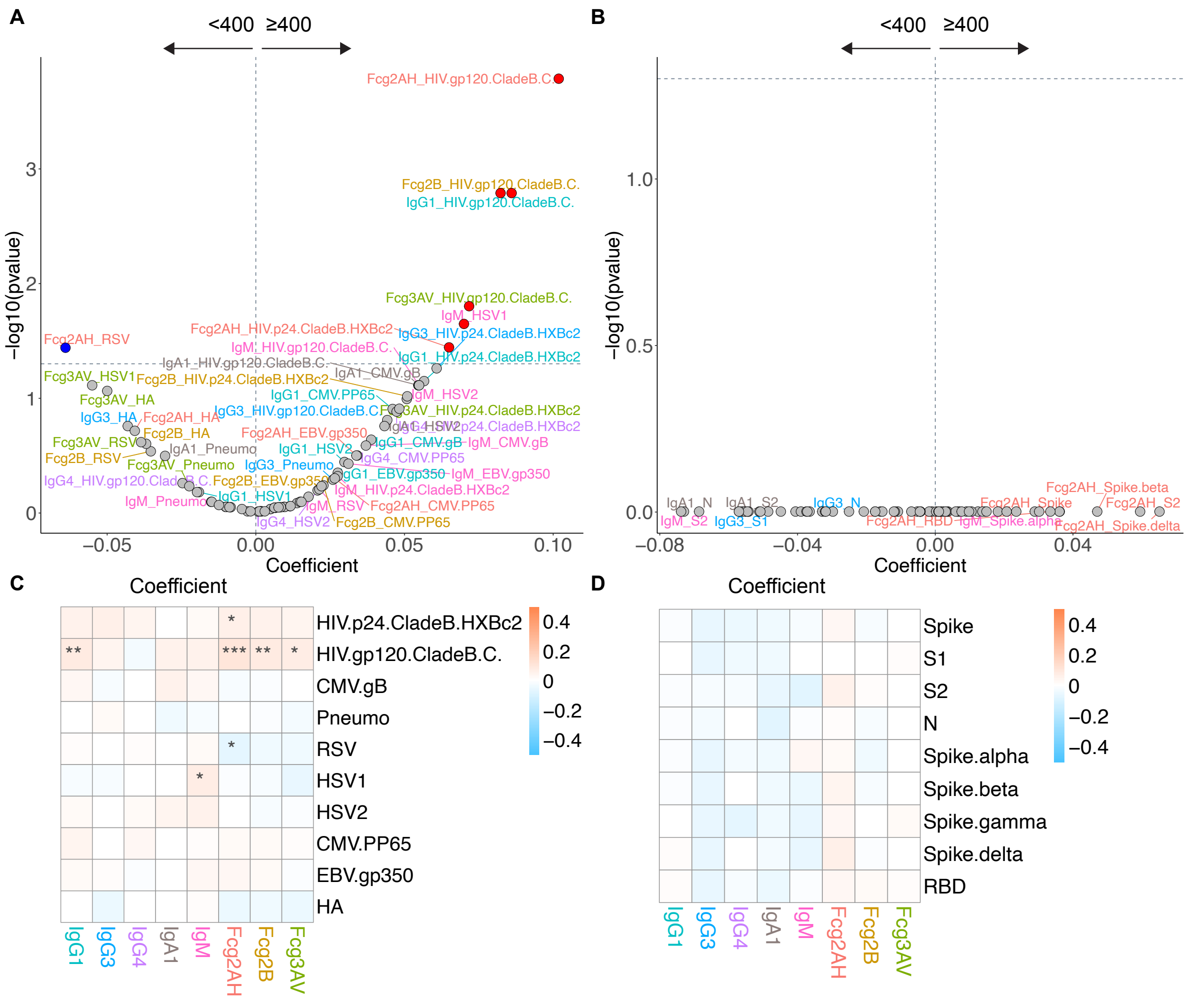

Figure S31. Volcano plots and heat maps of effect of HIV viremia on the humoral immune repertoire. Sensitivity analysis of Figure 9. Participants with COVID symptoms and IgG/IgA- are removed from the group with RBD IgG/IgA-. Adjusted volcano plots of effect of HIV viremia (≥400 vs <400 copies/ml) on the **(A)** non-SARS-CoV-2 humoral repertoire among the COVID-negative cohort and **(B)** SARS-CoV-2 humoral repertoire among the COVID-positive cohort. Respective heat maps of the volcano plot coefficients for the **(C)** non-SARS-CoV-2 and **(D)** SARS-CoV-2 humoral responses. See Figure 3 legend for details.

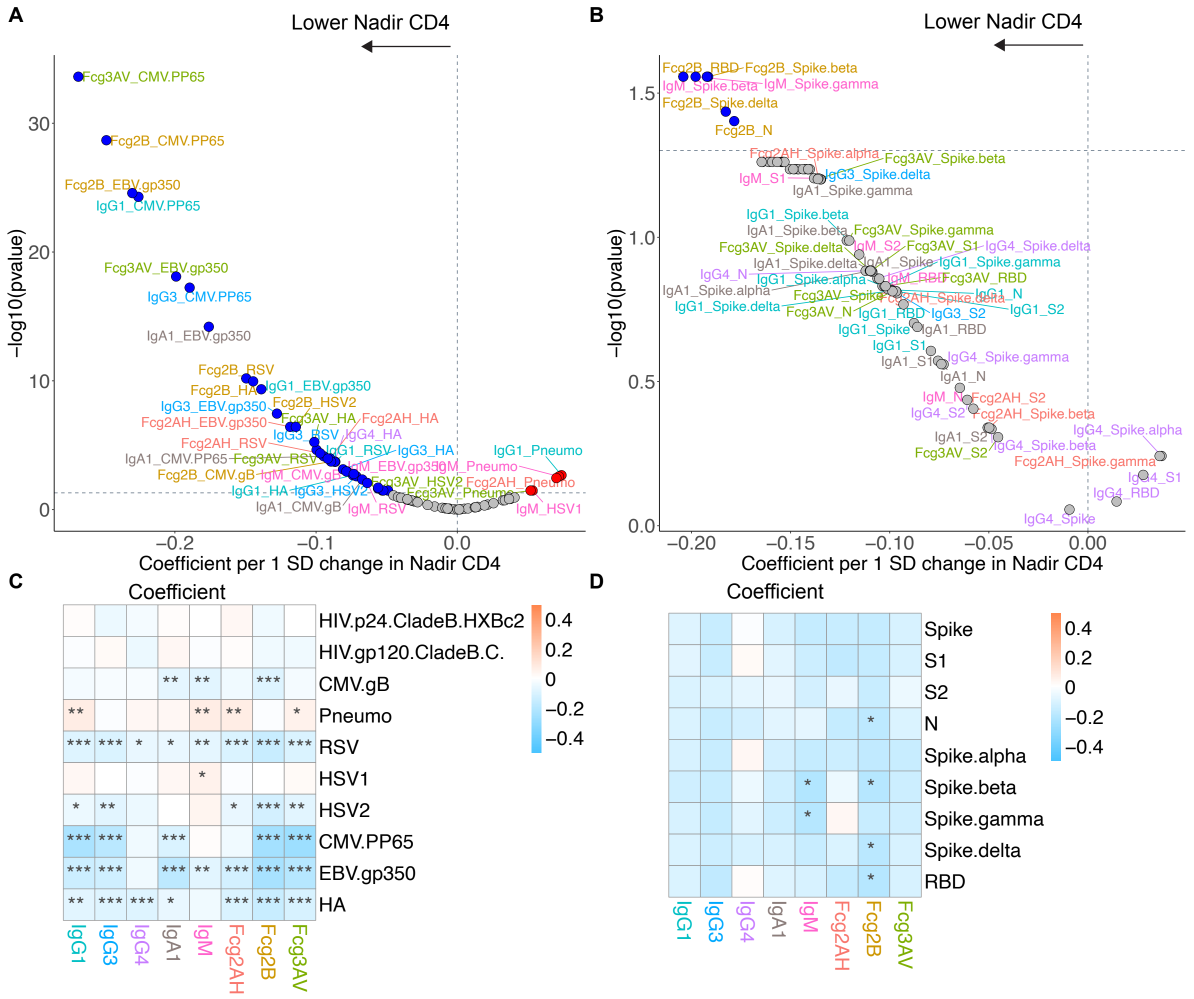

Figure S32. Volcano plots and heat maps of effect of nadir CD4 on the humoral immune repertoire. Sensitivity analysis of Figure 10. Participants with COVID symptoms and IgG/IgA- are removed from the group with RBD IgG/IgA-**.** Adjusted volcano plots of effect of nadir CD4 on the **(A)** non-SARS-CoV-2 humoral repertoire among the COVID-negative cohort and **(B)** SARS-CoV-2 humoral repertoire among the COVID-positive cohort. Coefficients reflect the effect of a 1 SD increase in nadir CD4, which was z-scored for each participant from the ordinal scale of <50, 50-199, 200-349, or ≥350 cell/mm^3. Respective heat maps of the volcano plot coefficients for the **(C)** non-SARS-CoV-2 and **(D)** SARS-CoV-2 humoral responses. See Figure 3 legend for details.

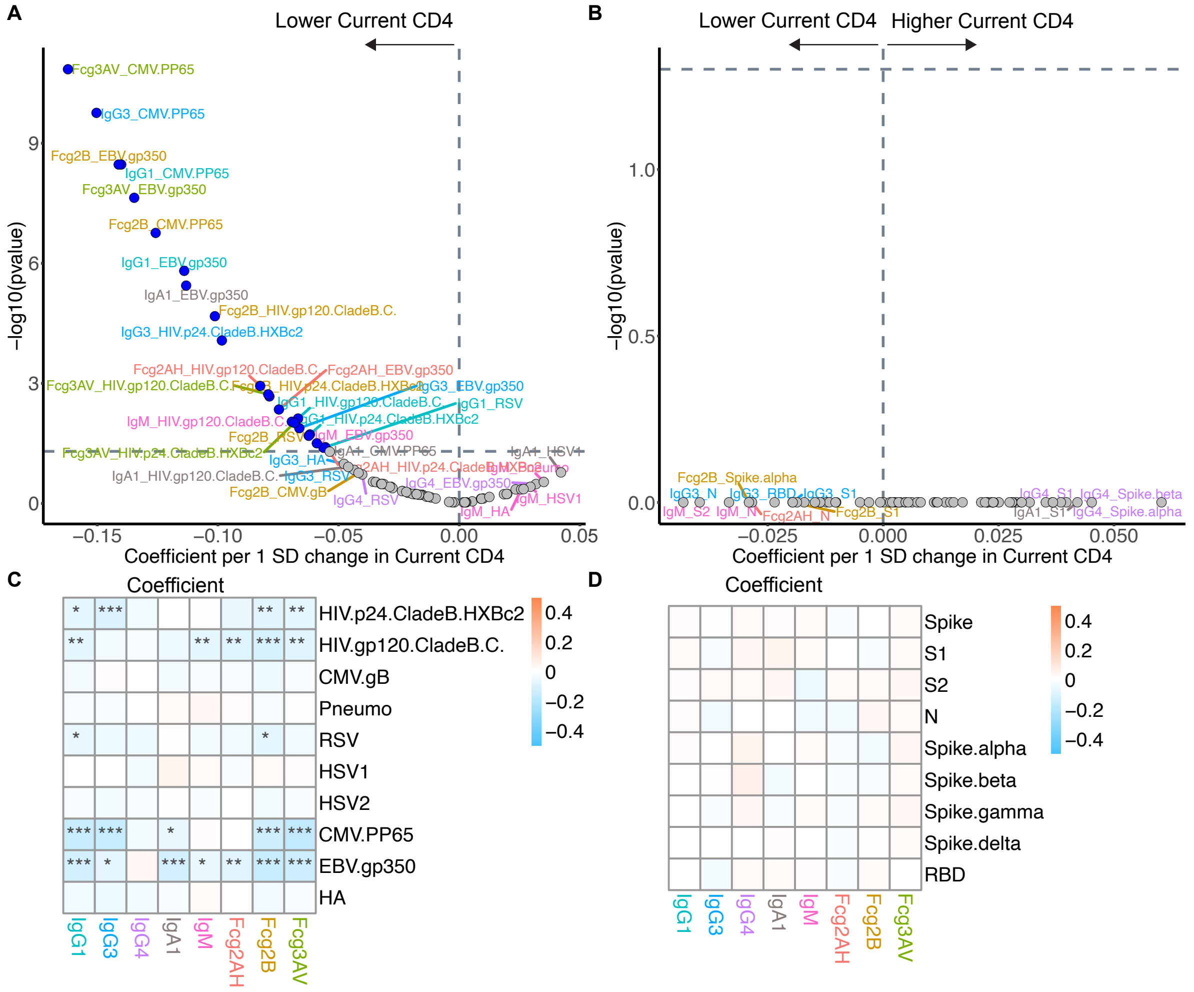

Figure S33. Volcano plots and heat maps of effect of current CD4 on the humoral immune repertoire. Sensitivity analysis of Figure 11. Participants with COVID symptoms and IgG/IgA- are removed from the group with RBD IgG/IgA-**.** Adjusted volcano plots of effect of current CD4 (without nadir CD4 adjustment) on the **(A)** non-SARS-CoV-2 humoral repertoire among the COVID-negative cohort and **(B)** SARS-CoV-2 humoral repertoire among COVID-positive cohort. Coefficients reflect the effect of a 1 SD increase in current CD4 (cells/mm^3), which was z-scored for each participant. Respective heat maps of the volcano plot coefficients for the **(C)** non-SARS-CoV-2 and **(D)** SARS-CoV-2 humoral responses. See Figure 3 legend for details.

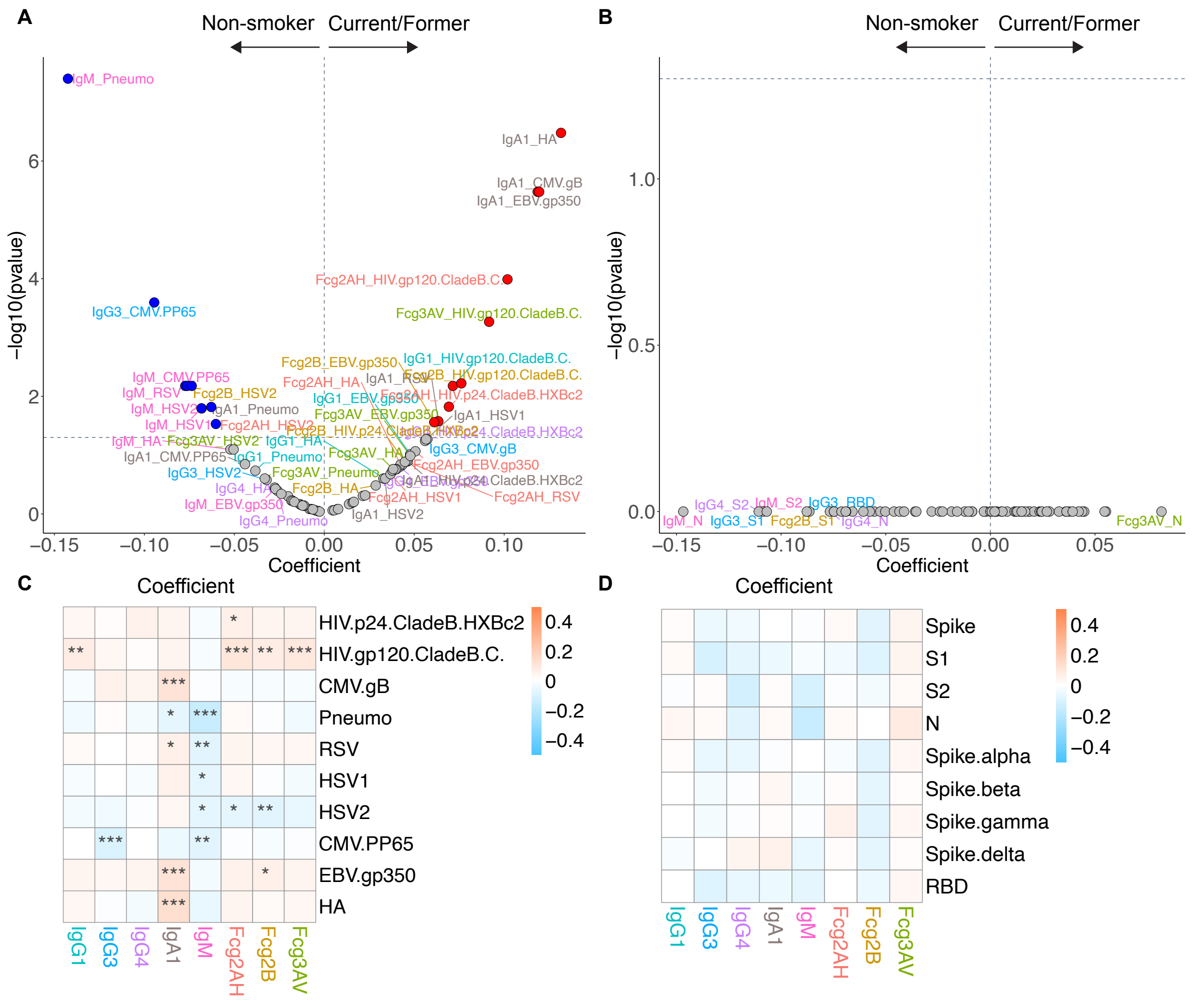

Figure S34. Volcano plots and heat maps of effect of smoking on the humoral immune repertoire. Sensitivity analysis of Figure S10. Participants with COVID symptoms and IgG/IgA- are removed from the group with RBD IgG/IgA-**.** Volcano plot of effect of smoking on the **(A)** non-SARS-CoV-2 humoral repertoire among the COVID-negative cohort and **(B)** SARS-CoV-2 humoral repertoire among the COVID-positive cohort. Volcano plots constructed from linear regression models, adjusted for age, sex, GBD region, nadir CD4, and HIV viral load, with horizontal dashed line of significance displayed for FDR-corrected P=0.05. Responses higher in current/former smoking fall toward the right of the vertical dashed line, while responses higher in never-smoking fall toward the left of the vertical dashed line. Respective heat maps of the volcano plot coefficients for the **(C)** non-SARS-CoV-2 and **(D)** SARS-CoV-2 humoral responses. Coefficients >0 are higher in current/former smoking, while coefficients <0 are higher in never-smoking. Significance in the heat maps is shown as FDR-corrected P<0.05 (*), P<0.01 (**), or P<0.001 (***). Specific antibody isotype, subclass, and Fc-receptor responses are color-coded between the volcano plots and heat maps.

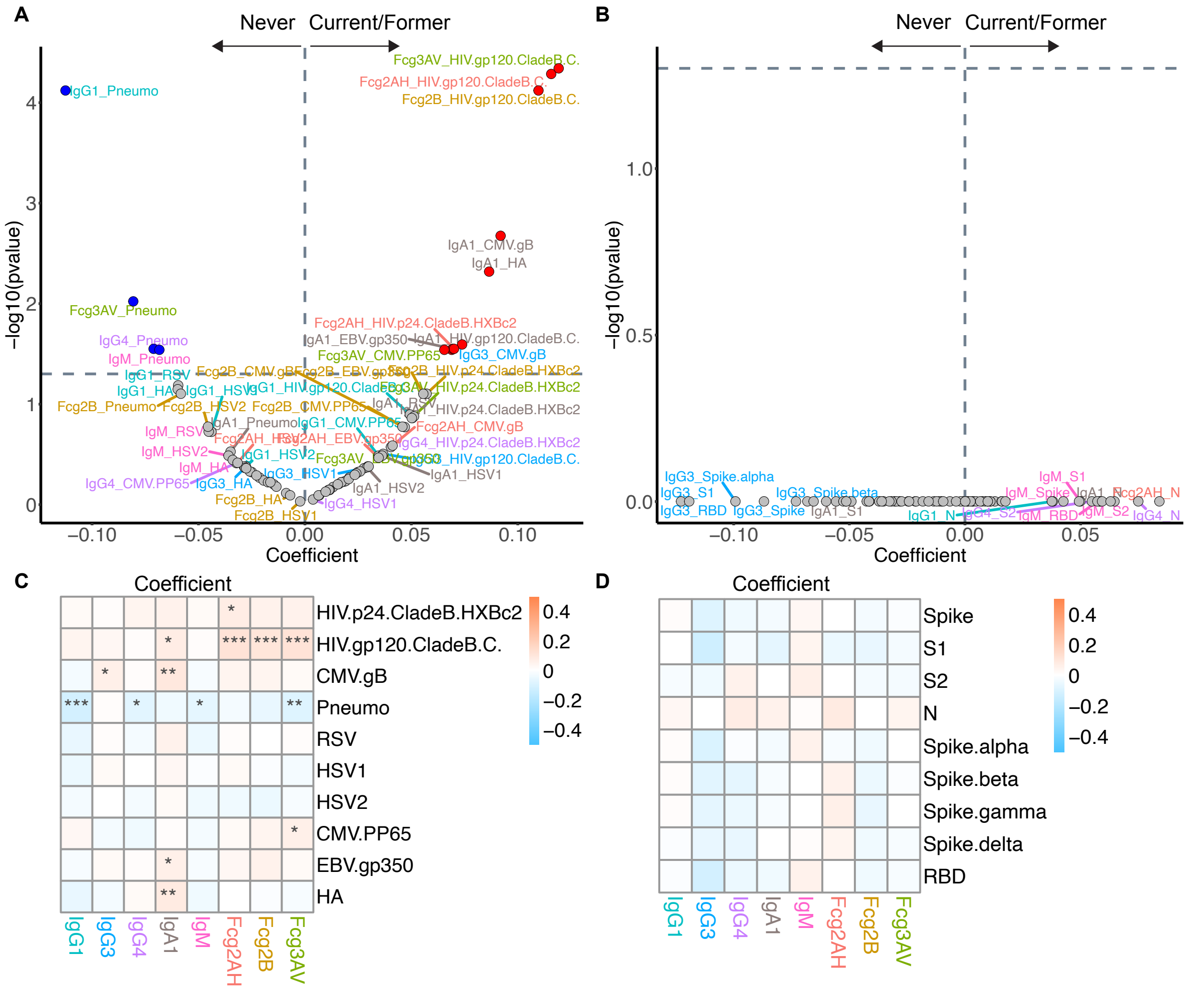

Figure S35. Volcano plots and heat maps of effect of substance use on the humoral immune repertoire. Sensitivity analysis of Figure S11. Participants with COVID symptoms and IgG/IgA- are removed from the group with RBD IgG/IgA-. Volcano plot of effect of substance use on the **(A)** non-SARS-CoV-2 humoral repertoire among the COVID-negative cohort and **(B)** SARS-CoV-2 humoral repertoire among the COVID-positive cohort. Volcano plots constructed from linear regression models, adjusted for age, sex, GBD region, nadir CD4, and HIV viral load, with horizontal dashed line of significance displayed for FDR-corrected P=0.05. Responses higher in current/former substance use fall toward the right of the vertical dashed line, while responses higher in never-substance use fall toward the left of the vertical dashed line. Respective heat maps of the volcano plot coefficients for the **(C)** non-SARS-CoV-2 and **(D)** SARS-CoV-2 humoral responses. Coefficients >0 are higher in current/former substance use, while coefficients <0 are higher in never-substance use. Significance in the heat maps is shown as FDR-corrected P<0.05 (*), P<0.01 (**), or P<0.001 (***). Specific antibody isotype, subclass, and Fc-receptor responses are color-coded between the volcano plots and heat maps.

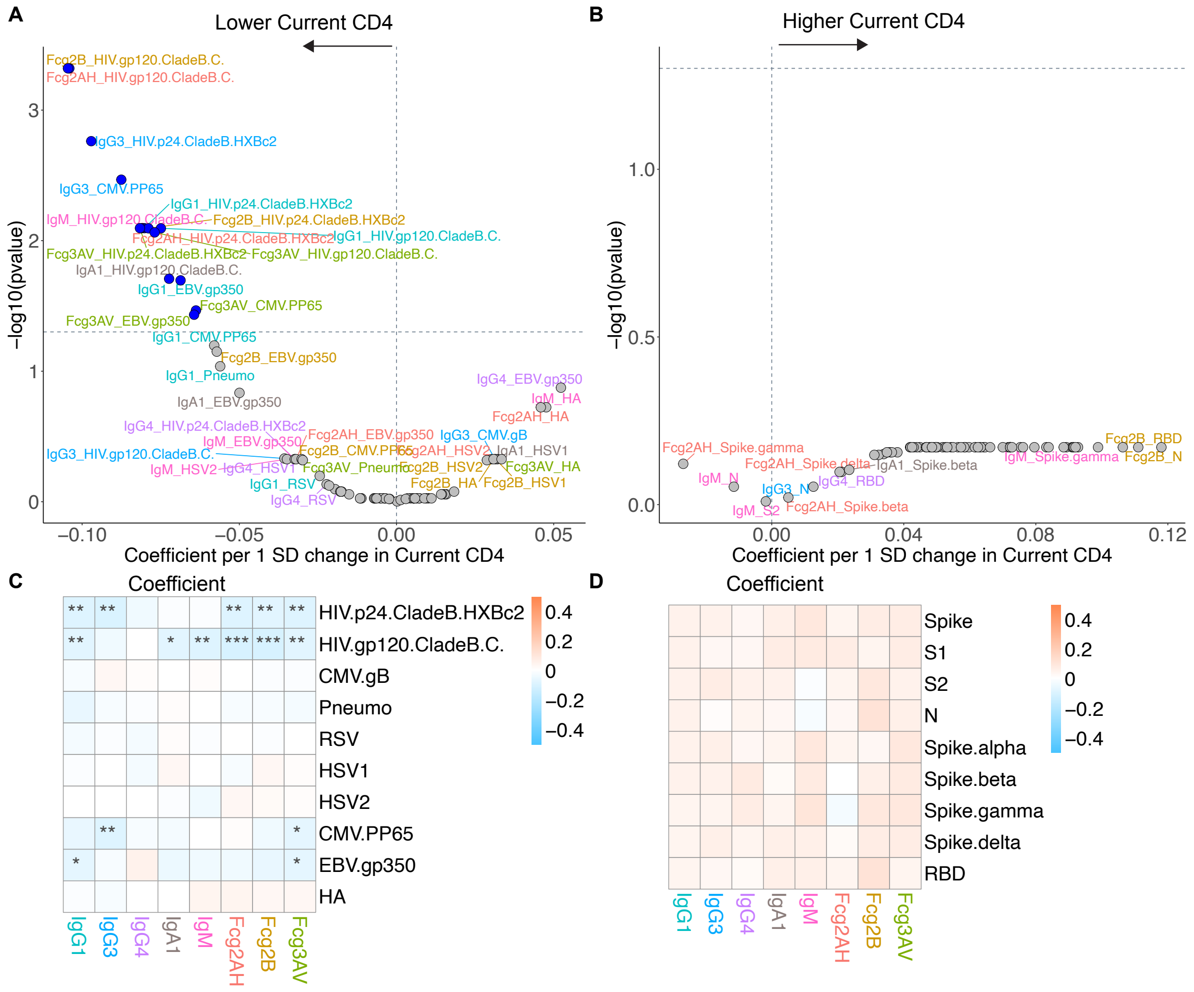

Figure S36. Volcano plots and heat maps of effect of current CD4 adjusted for nadir CD4 on the humoral immune repertoire. Sensitivity analysis of Figure S12. Participants with COVID symptoms and IgG/IgA- are removed from the group with RBD IgG/IgA-**.** Volcano plot of effect of current CD4 adjusted for nadir CD4 on the **(A)** non-SARS-CoV-2 humoral repertoire among the COVID-negative cohort and **(B)** SARS-CoV-2 humoral repertoire among COVID-positive cohort. Volcano plots constructed from linear regression models, adjusted for age, sex, GBD region, nadir CD4, and HIV viral load, with horizontal dashed line of significance displayed for FDR-corrected P=0.05. Coefficients reflect the effect of a 1 SD increase in current CD4 (cells/mm^3), which was z-scored for each participant. Responses higher with higher current CD4 fall toward the right of the vertical dashed line, while responses higher with lower current CD4 fall toward the left of the vertical dashed line. Respective heat maps of the volcano plot coefficients for the **(C)** non-SARS-CoV-2 and **(D)** SARS-CoV-2 humoral responses. Coefficients >0 are higher with higher current CD4, while coefficients <0 are higher with lower current CD4. Significance in the heat maps is shown as FDR-corrected P<0.05 (*), P<0.01 (**), or P<0.001 (***). Specific antibody isotype, subclass, and Fc-receptor responses are color-coded between the volcano plots and heat maps.
